# Structure-activity relationship of Cu(II) and Pd(II) thiosemicarbazone complexes with anticancer and antibacterial properties

**DOI:** 10.1080/14756366.2026.2742455

**Published:** 2026-10-15

**Authors:** Bartłomiej Rogalewicz, Magdalena Iwan, Marcin Świątkowski, Mariola Michalczuk, Joanna Kubik, Ewelina Humeniuk, Agnieszka Korga-Plewko, Monika Pitucha, Tomasz Boruta, Tomasz Sierański, Jakub Kubicki, Sylwia Ścieszka, Edyta Kordialik-Bogacka, Agnieszka Czylkowska

**Affiliations:** ^a^ Institute of General and Ecological Chemistry, Faculty of Chemistry, Lodz University of Technology, Łódź, Poland; ^b^ Department of Toxicology, Medical University of Lublin, Lublin, Poland; ^c^ Independent Medical Biology Unit, Medical University of Lublin, Lublin, Poland; ^d^ Independent Radiopharmacy Unit, Faculty of Pharmacy, Medical University of Lublin, Lublin, Poland; ^e^ Faculty of Process and Environmental Engineering, Department of Bioprocess Engineering, Lodz University of Technology, Łódź, Poland; ^f^ Institute of Fermentation Technology and Microbiology, Faculty of Biotechnology and Food Sciences, Lodz University of Technology, Łódź, Poland

**Keywords:** Anticancer activity, antibacterial activity, structure–activity relationship, thiosemicarbazones, copper(II) complexes, palladium(II) complexes

## Abstract

Twelve thiosemicarbazones and 24 corresponding Cu(II) and Pd(II) complexes were synthesised and evaluated for their physicochemical properties and biological activities. Single-crystal X-ray diffraction and mass spectrometry confirmed ligand deprotonation and the formation of neutral ML_2_-type complexes. Several complexes containing 3,4-dimethoxybenzylidene- or 4-bromobenzylidene-substituted ligands exhibited submicromolar cytotoxicity against melanoma cells. **Cu(b-TSC)_2_** and **Pd(dm-TSC-pCl)_2_** were selected for mechanistic studies and induced cell-cycle arrest, pro-apoptotic gene activation, DNA damage, and apoptosis. Notably, **Cu(b-TSC)_2_** retained activity against vemurafenib-resistant SK-MEL-28 and A-375 cells. Structure-activity analysis showed that additional chlorine substitution generally reduced anticancer activity. Calculated physicochemical descriptors suggested that increased chlorination enhanced lipophilicity and reduced hydrophilicity, which was associated with lower apparent solution stability. These findings highlight the importance of balancing lipophilicity, hydrophilicity, and solution stability in future design of potential drugs based on thiosemicarbazones. In contrast, antibacterial activity was more strain-dependent, with MIC values against *E. coli* as low as 3.125 mg/L.

## Introduction

In modern medicinal chemistry, metal-based compounds have emerged as some of the most promising candidates in the development of new therapeutics^1–3^. The ever-growing number of articles highlights their potential in addressing some of the most pressing global health problems, including cancer, bacterial infections, and increasing multidrug resistance. The International Agency for Research on Cancer predicts that the overall number of new cases will increase from 20.0 million in 2022 to 24.1 million in 2030, and to 29.9 million in 2040^4^. In parallel, it is estimated that in 2019, bacterial infections caused by antibiotic-resistant strains were directly responsible for 1.27 million deaths globally and were associated with a total of 4.95 million deaths^5^. Considering these statistics, the search for new, more active, and safer therapeutics remains one of the most pressing challenges. Simultaneously, metal complexes and organometallic compounds often exhibit notably enhanced biological activity compared to free organic ligands. In this context, the inclusion of metal ions is known to significantly influence key features such as bioavailability and toxicity of the potential drugs, enabling the fine-tuning of their properties, which is especially important given the evasive nature of cancer cells and resistant bacterial strains.

Among d-electron metals, Cu(II) complexes are some of the most extensively studied, both due to their notable anticancer and antimicrobial activities^6–8^. Their major advantage lies in their ability to act through multiple mechanisms, including ROS production *via* Cu(II)/Cu(I) redox cycling, DNA binding and cleavage, and enzyme inhibition^6^^,^^9^. Moreover, since Cu(II) is a biogenic metal, its complexes often exhibit a favourable pharmacokinetic profile and improved biocompatibility. So far, heteroligand Cu(II) complexes based on 2,2′-bipyridine and 1,10′-phenanthroline derivatives, in combination with different *N*,*O*-donor chelators, are undergoing clinical trials under the name of Casiopeinas^10^^,^^11^. Similarly, Pd(II) complexes, although less common, are increasingly recognised as promising metallodrugs, particularly as alternatives to often more toxic Pt(II)-based anticancer agents. Since both Pd(II) and Pt(II) belong to group 10 of the periodic table, their physicochemical characteristics are broadly similar. Their anticancer mechanisms of action are likewise comparable, primarily involving interaction with DNA, leading to single- and/or double-strand breaks, ultimately triggering apoptosis^12^. Both Cu(II) and Pd(II) ions readily form complexes with various *N*- and *S*-donor ligands. Thiosemicarbazones, in particular, offer a broad range of versatile ligands with diverse coordination properties and biological activities^13–15^. Moreover, their synthetic accessibility allows for fine-tuning of their physicochemical and biochemical characteristics, which has sustained considerable interest in these compounds. Up to this point, several compounds containing thiosemicarbazone scaffold have reached clinical phase trials as possible anticancer drugs, further underscoring their therapeutic potential. Some of the most common are depicted in Figure 1 and include: Triapine (3-AP), a potent ribonucleotide reductase inhibitor^16^; COTI-2, capable of reactivating mutant p53 to a wild-type form^17^; and DpC, exhibiting reduced overall systemic toxicity and improved bioavailability^18^.

**Figure 1.**
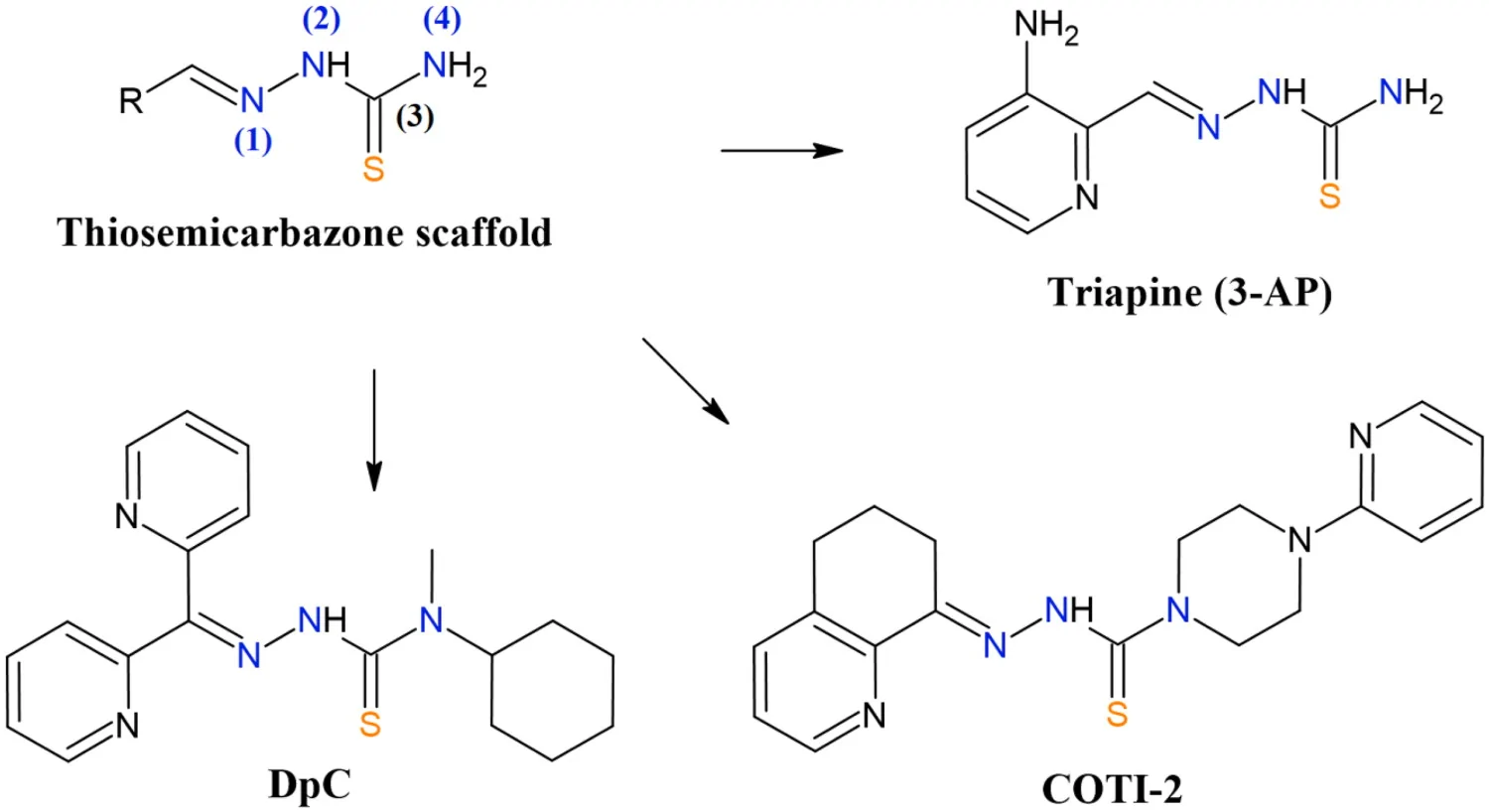
General structure of the thiosemicarbazone scaffold and representative thiosemicarbazone derivatives with significant anticancer activity. Blue labels in parentheses indicate the conventional numbering of the thiosemicarbazone chain used to describe substitution patterns.

So far, many d-electron metal complexes containing thiosemicarbazones have been reported, some of which exhibit significant anticancer activity and enzyme inhibitory effects, particularly towards Topoisomerase II^19^. According to the literature, most thiosemicarbazone-based complexes are tridentate, with *N*,*N,S*-coordinating modes, usually yielding M(HL)Cl_2_- or M(L)Cl-type complexes (where HL denotes the parent thiosemicarbazone ligand), or ML-type complexes bearing bis(thiosemicarbazone) ligands. In contrast, bidentate ML_2_-type complexes are considerably less common. While most of the existing literature on such complexes focuses on their physicochemical characterisation and spectral properties^20–24^, in recent years there has been a notable increase in reports covering their biological properties, including antitumor^25–28^, antibacterial^29^^,^^30^, antifungal^29^, anti-inflammatory^29^, antioxidant^31^, antimalarial^31^, and antituberculosis^29^.

Some of these studies report significant anticancer activity^28^^,^^32^^,^^33^, exceeding that of standard reference drugs^27^^,^^32^^,^^33^. Although several studies have led to the identification of important structure–activity trends in thiosemicarbazones^34^^,^^35^, corresponding studies on ML_2_-type complexes remain scarce. At the same time, the physicochemical and biological properties of such complexes can be modulated through the introduction of different substituents at the N1 and N4 positions of the thiosemicarbazone scaffold. Therefore, the relationships between ligand substitution, metal identity, solution behaviour, and biological activity in ML_2_-type complexes remain insufficiently understood. This study was designed to address this gap.

In one of our previous studies, we investigated the anticancer and antibacterial potential of *N*,*S*-coordinated complexes of Ni(II), Cu(II), Zn(II), Pd(II), and Ag(I) containing thiosemicarbazones substituted at both the N1 and N4 positions^36^. Among the tested compounds, the Cu(II) and Pd(II) complexes showed the most promising anticancer activity and were therefore selected for further investigation. In addition, these complexes appeared to exert their cytotoxic effects through distinct mechanisms of action, involving mainly ROS generation and DNA damage, respectively. In the present study, we synthesised analogous Cu(II) and Pd(II) ML_2_-type complexes containing different substituents at the N1 and N4 positions of the thiosemicarbazone scaffold. Given the high malignancy of melanoma and its pronounced tendency to develop drug resistance, the anticancer activity of the complexes was evaluated against parental and vemurafenib-resistant melanoma cells. Their antibacterial activity was additionally assessed against a panel of bacterial strains.

## Results

### Design and synthesis of the complexes

The parent thiosemicarbazone ligands (**HTSCs**) were divided into three groups, according to the substituents present at the N1 position of the thiosemicarbazone scaffold:

- 3,4-dimethoxybenzylidene-substituted derivatives (Figure 2(a)),
- 3,4-dichlorobenzylidene-substituted derivatives (Figure 2(b)),
- 4-bromobenzylidene-substituted derivatives (Figure 2(c)).

**Figure 2.**
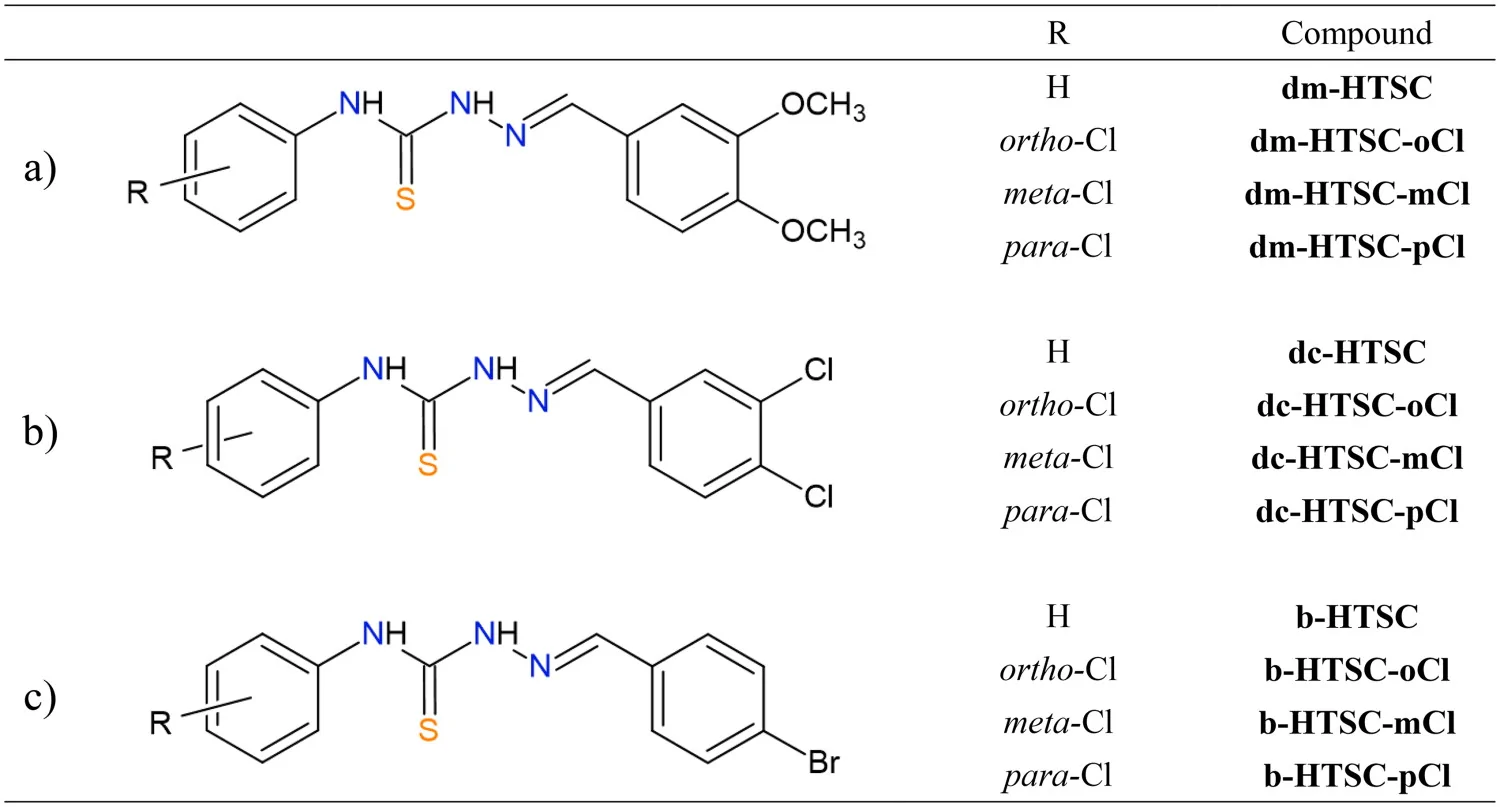
General structures of the parent thiosemicarbazone ligands: (a) 3,4-dimethoxybenzylidene derivatives; (b) 3,4-dichlorobenzylidene derivatives; (c) 4-bromobenzylidene derivatives.

Within each group, the phenyl substituent attached to the terminal N4 nitrogen was either unsubstituted or substituted with a chlorine atom at the *-ortho*, *-meta*, or *-para* position (R).

Subsequently, all ligands were converted into their corresponding Cu(II) and Pd(II) complexes in a single-step reaction (Figure 3), which resulted in the formation of ML_2_-type complexes, as confirmed by SC-XRD analysis, elemental analysis, and mass spectrometry.

**Figure 3.**
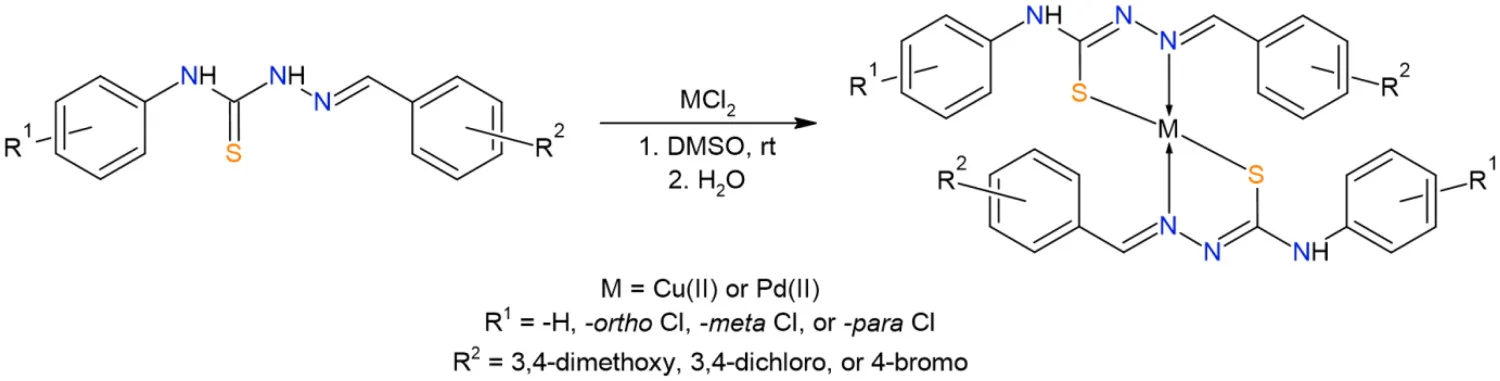
Synthesis route of the title Cu(II) and Pd(II) complexes.

#### SC-XRD analysis

The single-crystal X-ray diffraction analysis revealed the structures of five thiosemicarbazone derivatives (Figure 4). These compounds display structural features characteristic for the thiosemicarbazone family (Table S1). Specifically, the N3—C8 bond lengths align with typical imine double bond distances, while C7—S1 bond distances correspond to the thione form observed in thioureas [1.279(8) Å and 1.681(20) Å, respectively]^36^. Across five investigated structures, the C1—N1—C7—N2—N3—C8—C9 chain adopts an *antiperiplanar–synperiplanar–antiperiplanar–antiperiplanar* (*ap–sp–ap–ap*) conformational sequence corresponding to the successive torsion angles (Table S2), which is stabilised by intramolecular N1—H•••N3 hydrogen bond with S(5) motif (Table S3). The nearly planar arrangement of the hydrogen atoms in the N1 and N2 amino groups indicates sp^2^ hybridisation of these nitrogen atoms, confirming the conjugation of their lone electron pairs with the π-electron systems of the imine and thione bonds. Variations in overall molecular planarity were observed among the studied thiosemicarbazones. The **dm-HTSC-oCl** molecule, along with two of the four independent molecules found in the asymmetric unit of **dm-HTSC**, exhibits the highest degree of planarity. In the remaining structures, significant deviations from planarity occur, primarily attributed to the rotation of the aromatic ring attached to the N1 nitrogen atom relative to the thiosemicarbazone core. The interplanar angles between the mean planes of the two aromatic rings in these less planar molecules span a considerable range, from approximately 35° to 76° (Table S2). The supramolecular assembly of these thiosemicarbazones is predominantly stabilised by R^22(8)^ dimeric hydrogen bonding synthons (Figure S1), which are formed between the N2 amino group of one molecule and the S1 thione sulphur atom of a neighbouring molecule (Table S3).

**Figure 4.**
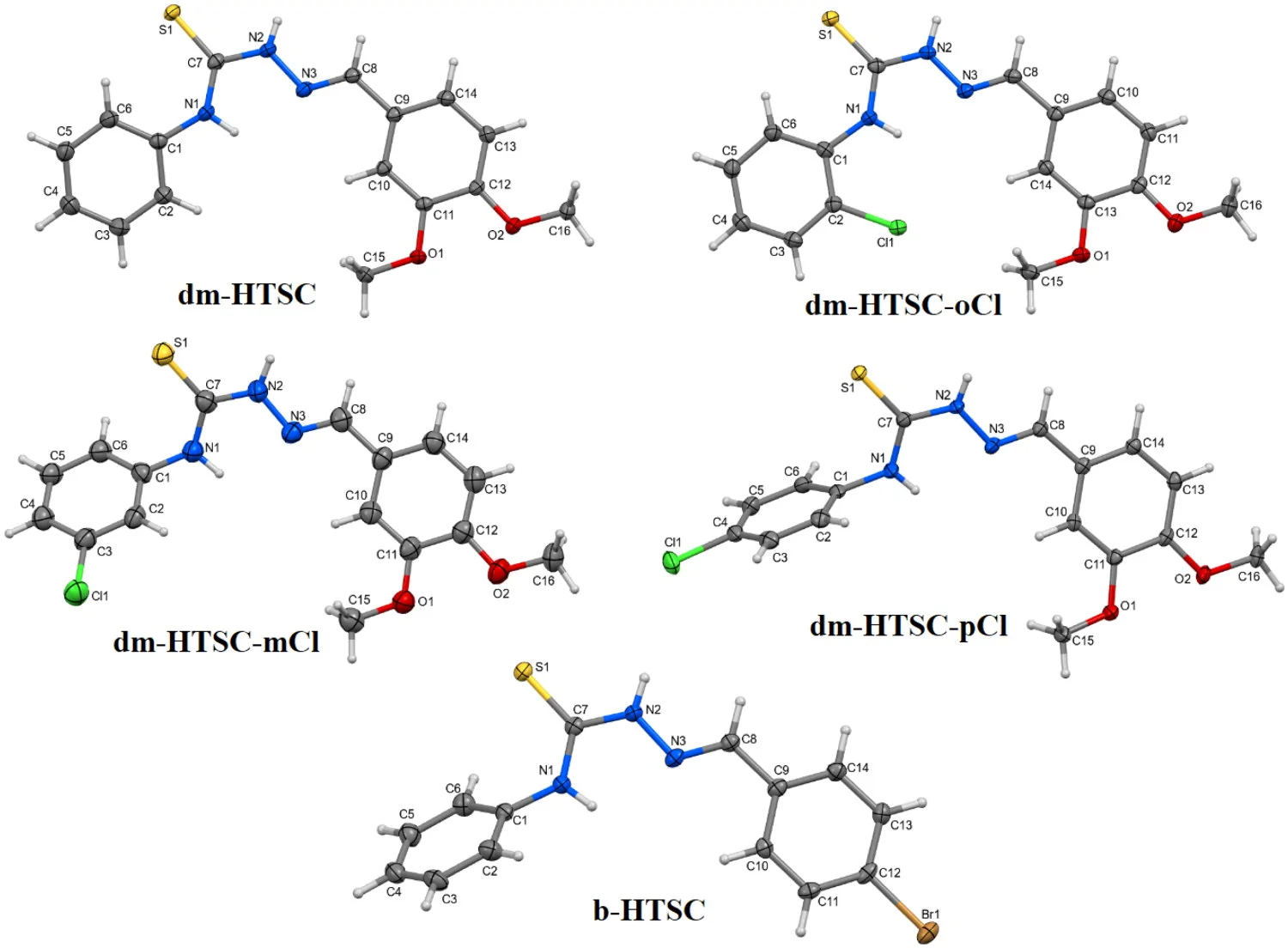
Molecular structures of thiosemicarbazone derivatives: **dm-HTSC**, **dm-HTSC-oCl**, **dm-HTSC-mCl**, **dm-HTSC-pCl**, **b-HTSC**, with atom labelling and displacement ellipsoids drawn at the 50% probability level.

From the series of synthesised coordination compounds, crystallographic analysis was successfully carried out on three copper compounds and six palladium compounds (Figures 5 and 6). Despite differences in crystal symmetry and packing across the series, all complexes are mononuclear and share the same molecular coordination motif: each metal ion is chelated by two monoanionic thiosemicarbazonate ligands through the imine N3 and thiolate S1 donor atoms. Formation of the anionic ligand formally corresponds to thione–thiol tautomerisation, involving transfer of the N2-bound proton to S1, followed by deprotonation of the resulting thiol group and formation of an M–S(thiolate) bond. This transformation is reflected in the elongation of C7–S1 and N2–N3 bonds, accompanied by shortening of C7–N2 (Table S1). These changes indicate a redistribution of π-electron density within the chelating S1–C7–N2–N3 fragment, involving a decrease in the double-bond character of C7–S1 and a corresponding increase in that of C7–N2. Coordination is also accompanied by a change in the conformational sequence of the successive torsion angles along the C1—N1—C7—N2—N3—C8—C9 chain, from *ap–sp–ap–ap* in the neutral ligands to *sp–ap–ap–sp* in the complexes (Table S2). The coordination polyhedra consistently exhibit a square planar (*SP*-4) geometry (Table S5), which was confirmed with usage of SHAPE program^37^ utilising the Continuous Shape Measures (CShM) concept^38^. Crystallographically, the central atoms in all characterised structures are positioned at special positions of their respective space groups with site symmetry −1 (an inversion centre), resulting in half of the coordination entity in the asymmetric unit. Notably, two isomorphous subsets were identified on the basis of the same space-group setting, closely similar unit-cell parameters, and corresponding atomic arrangements (Table S6). The first isomorphous group comprises **Cu(dm-TSC)_2_**, **Pd(dm-TSC)_2_**, and **Pd(dm-TSC-pCl)_2_**, while the second group consists of **Cu(dm-TSC-oCl)_2_** and **Pd(dm-TSC-oCl)_2_**. This propensity for isomorphism can be rationalised by the similar ionic radii of the Cu^2+^ and Pd^2+^ cations within the observed four-coordinate, square planar (*SP*-4) geometry (Cu^2+^: 0.57 Å; Pd^2+^: 0.64 Å)^39^, which allows for comparable packing arrangements in the crystal lattice. The conformational transformation of the thiosemicarbazone chain and N2 deprotonation upon coordination substantially alter the hydrogen bonding patterns compared to the free ligands. N1 nitrogen remains the sole hydrogen bond donor in these coordination compounds. In complexes containing dimethoxybenzene rings, bifurcated hydrogen bonds form between the N1 amino group and two oxygen atoms of the methoxy groups (O1 and O2) serving as acceptors (Table S3), creating chain motifs C(10) and C^11^, respectively (Figure S2). In remaining compounds, the amino group forms hydrogen bonds with solvent molecules, such as DMSO or DMF, incorporated during the crystallisation process (Table S3). The absence of available classical hydrogen bond acceptors in the structure of coordinated thiosemicarbazones appears to be a primary factor favouring the formation of solvates for these compounds, as the inclusion of solvent molecules capable of accepting hydrogen bonds contributes significantly to the stabilisation of crystal lattices.

**Figure 5.**
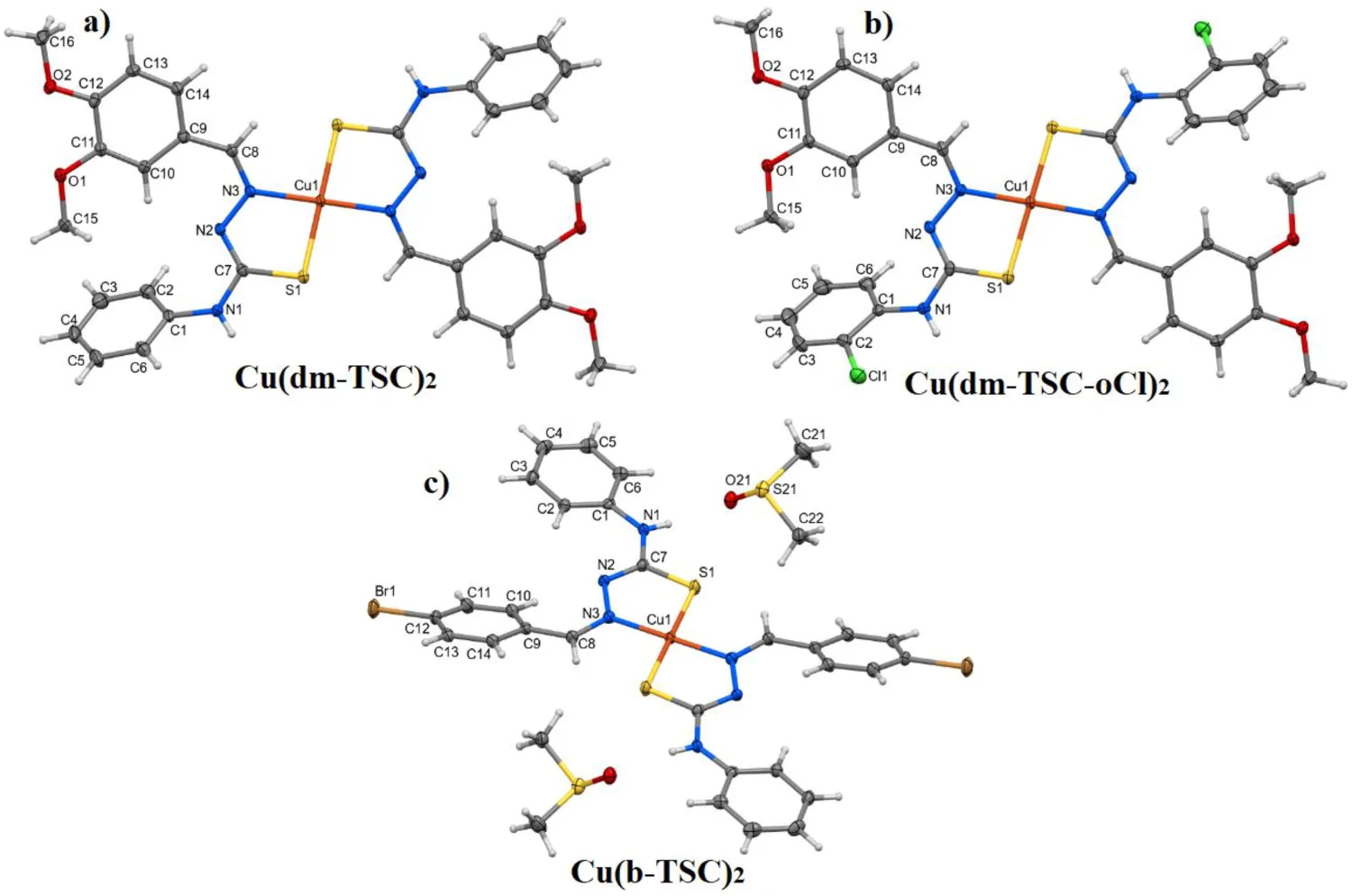
Molecular structures of copper coordination compounds: **Cu(dm-TSC)_2_**, **Cu(dm-TSC-oCl)_2_**, **Cu(b-TSC)_2_**·2DMSO, with atom labelling and displacement ellipsoids drawn at the 50% probability level. The equivalent atoms (without labels) were generated according to the following transformation: (a) -x,1-y,-z, (b) 1-x,1-y,-z, (c) 1-x,1-y,1-z.

**Figure 6.**
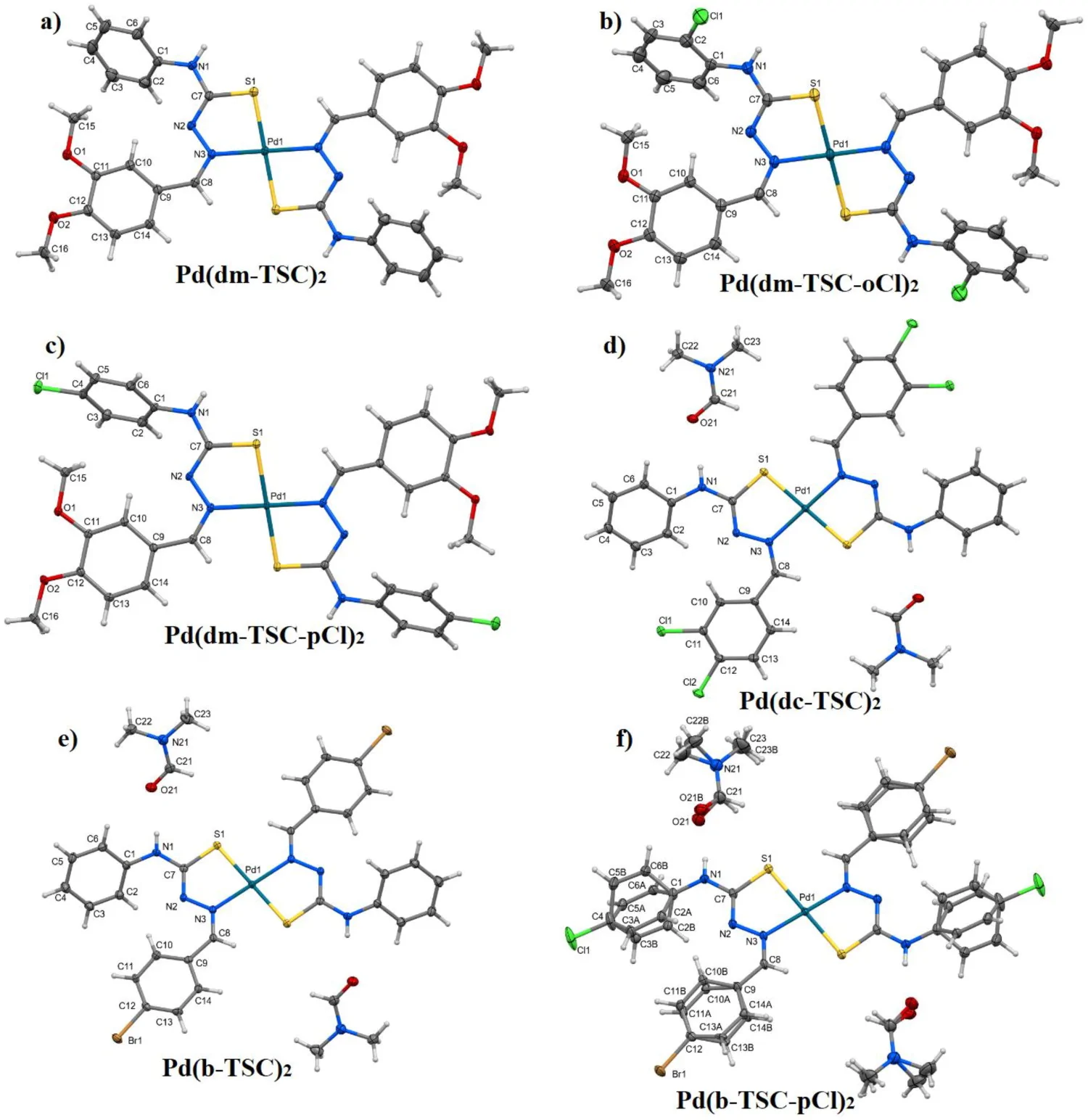
Molecular structures of palladium coordination compounds: **Pd(dm-TSC)_2_**, **Pd(dm-TSC-oCl)_2_**, **Pd(dm-TSC-pCl)_2_**, **Pd(dc-TSC)_2_**·2DMF, **Pd(b-TSC)_2_**·2DMF, **Pd(b-TSC-pCl)_2_**·2DMF, with atom labelling and displacement ellipsoids drawn at the 50% probability level. The equivalent atoms (without labels) were generated according to the following transformation: a) -x,1-y,-z, b) 1-x,1-y,-z, c) 1-x,1-y,2-z, d) 2-x,-y,1-z, e) 1-x,1-y,1-z, f) 1-x,1-y,1-z.

#### Mass spectrometry, FTIR spectra, and UV-vis-monitored stability

The structures of all complexes were confirmed by mass spectrometry, with the expected [M + H]^+^ species observed in all cases (where M denotes the neutral complex molecule) (Figures S3–S26). Additionally, the mass spectra of the Cu(II) complexes indicate partial reduction of Cu(II) to Cu(I), a known phenomenon under ESI-MS conditions^40^^,^^41^. As described by Płonka et al., this reduction leads to a decrease in positive charge, which is offset by the attachment of an additional proton^42^. Consequently, the most intense peak corresponds to an *m/z* value of +1, while the [M + H]^+^ peak appears with lower intensity.

The FTIR spectra of the Cu(II) and Pd(II) complexes are significantly altered compared to the spectra of the parent ligands (Figures S27–S35). The observed changes are analogous to those previously reported for structurally similar complexes based on thiosemicarbazones^36^. Briefly: in the complexes’ spectra, the *v*(NH) bands in the 3400–3250 cm^−1^ region are significantly reduced or shifted due to complexation and ligand deprotonation. Below 3250 cm^−1^, multiple bands are ascribed to aliphatic and aromatic *v*(CH) modes. Similarly, in the region between 1600 and 1250 cm^−1^, several bands with varying intensities correspond to *v*(C = N), *v*(CC), *δ*(CH), and *δ*(NH). Lower-intensity bands below 1250 cm^−1^ are typical for *v*(CS), *v*(C-N), and *v*(NN) modes.

The stability of all complexes was monitored using UV-Vis spectroscopy after 0, 24, and 48 h of stabilisation in a mixed 50:50 (v/v) H_2_O/DMSO medium (Figures S36–S41). In all cases, the Pd(II) complexes exhibited superior stability in solution over time, compared to their corresponding Cu(II) analogues. All spectra contain a prominent maximum between 300 and 400 nm, typical for the ^1^π→π^∗^ intraligand transitions^23^. An overlapping band with significantly lower intensity around 400 nm, particularly well visible in the case of complexes containing 3,4-dichlorobenzylidene- and 4-bromobenzylidene-substituted ligands, is most likely connected with metal-ligand or ligand-metal charge-transfers (MLCT/LMCT) ^20^. Similarly, the stability of all complexes was assessed in a 90:10 (v/v) DCBM/DMSO medium (Figures S42–S47). While the overall spectral profiles were largely retained throughout the experiment, the complexes exhibited limited apparent stability, particularly those containing 3,4-dichlorobenzylidene ligands, as evidenced by a pronounced decrease in absorbance over time. The observed changes are consistent with partial aggregation and/or precipitation of the dissolved complexes, in agreement with previous literature reports^36^.

### In silico analysis of physicochemical and ADME-related descriptors

An *in silico* analysis of physicochemical and ADME-related descriptors was performed for all parent ligands. Figure 7 presents the bioavailability radars based on six key molecular properties: lipophilicity, size, polarity, solubility, unsaturation, and flexibility. From a medicinal chemistry perspective, these parameters may influence a compound’s ADME profile – absorption, distribution, metabolism, and excretion – and consequently its bioavailability, systemic exposure, membrane permeability, and metabolic stability. DpC was included as a benchmark because it is a structurally related thiosemicarbazone with a well-established pharmacokinetic profile. Since the presence of the chloro substituent had a greater effect on the evaluated parameters than its position, the bioavailability radars for the remaining *meta*- and *para*-chloro-substituted ligands are provided in the (Supplementary Materials Figure S48).

**Figure 7.**
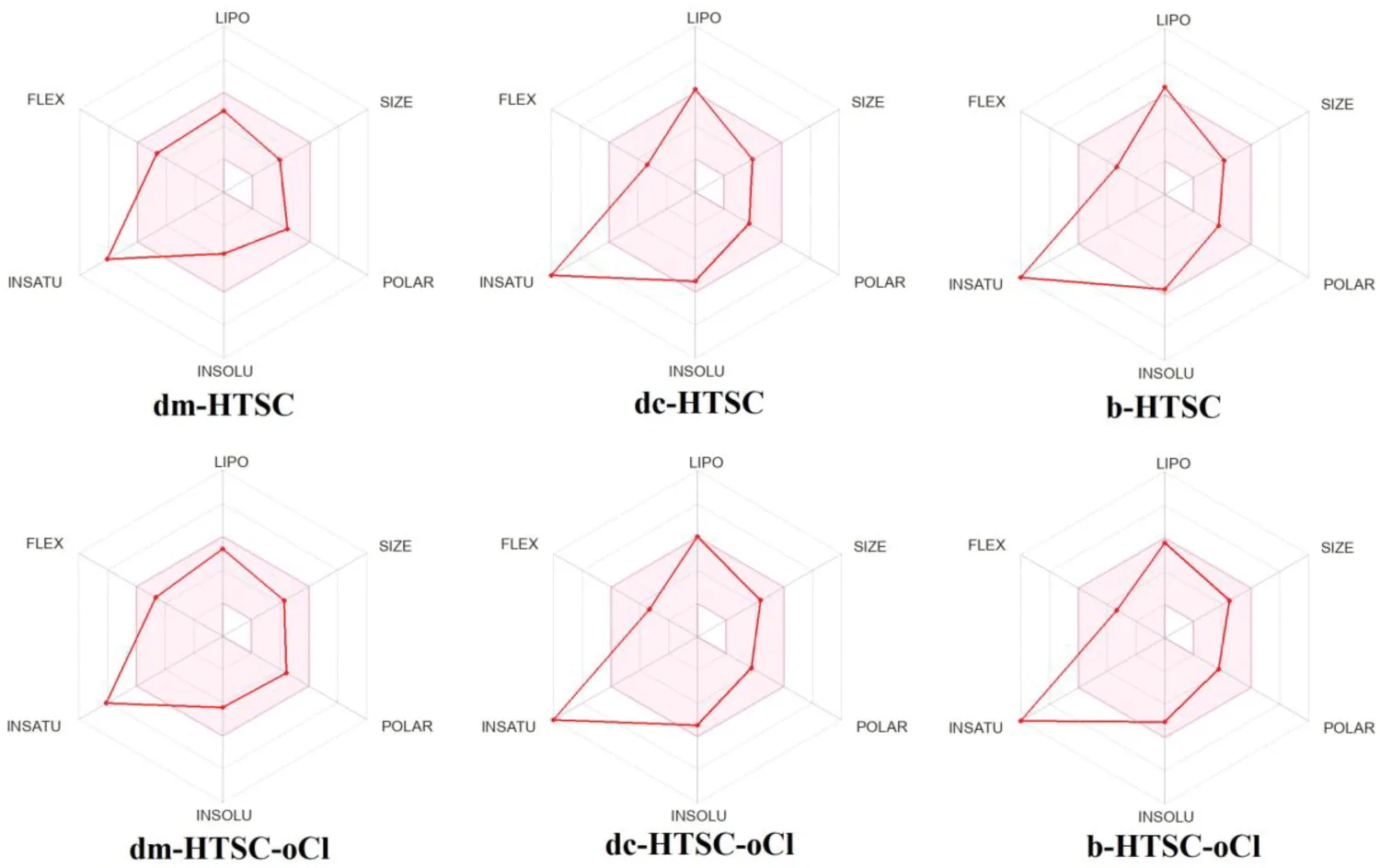
Bioavailability radars generated for ligands containing 3,4-dimethoxybenzylidene substituents (**dm-HTSC**, **dm-HTSC-oCl**), 3,4-dichlorobenzylidene substituents (**dc-HTSC**, **dc-HTSC-oCl**), and 4-bromobenzylidene substituents (**b-HTSC**, **b-HTSC-oCl**). The shaded region represents the optimal physicochemical range for oral bioavailability according to the SwissADME model. LIPO – lipophilicity (−0.7 *<* XlogP3 *<* +5.0); SIZE – molecular weight (150 g/mol *<* MW *<* 500 g/mol); POLAR – polarity (20 Å^2^ *<* TPSA *<* 130 Å^2^); INSOLU – insolubility (−6 *<* logS *<* 0); INSATU – fraction of sp^3^-hybridized carbon atoms (0.25 *<* fraction Csp^3^ *<* 1); FLEX – flexibility (0 *<* num. of rotatable bonds *<* 9).

According to the calculations, all ligands are predicted to satisfy the Lipinski^43^, Ghose^44^, Veber^45^, and Egan^46^ drug-likeness criteria. Most of the analysed parameters fall within the physicochemical range considered favourable for oral bioavailability, represented by the shaded region of the bioavailability radar plots. Among the three groups of investigated ligands, the most favourable overall profiles were observed for the 3,4-dimethoxybenzylidene derivatives. Compared with the DpC benchmark (Figure S49), the investigated ligands showed slightly less favourable radar profiles, mainly because their lower predicted fraction of sp^3^-hybridized carbon atoms corresponds to greater molecular unsaturation. To obtain preliminary predictions concerning the absorption and distribution properties of the ligands, a BOILED-Egg diagram was generated and analysed (Figure 8). All ligands were located within the regions associated with a high probability of gastrointestinal absorption. The 3,4-dichlorobenzylidene- and 4-bromobenzylidene-substituted ligands were additionally located within the region associated with predicted blood-brain barrier permeation. These results should be regarded as computational hypotheses requiring experimental validation rather than as evidence of *in vivo* absorption or brain penetration. According to the model, none of the investigated ligands was classified as a P-glycoprotein substrate.

**Figure 8.**
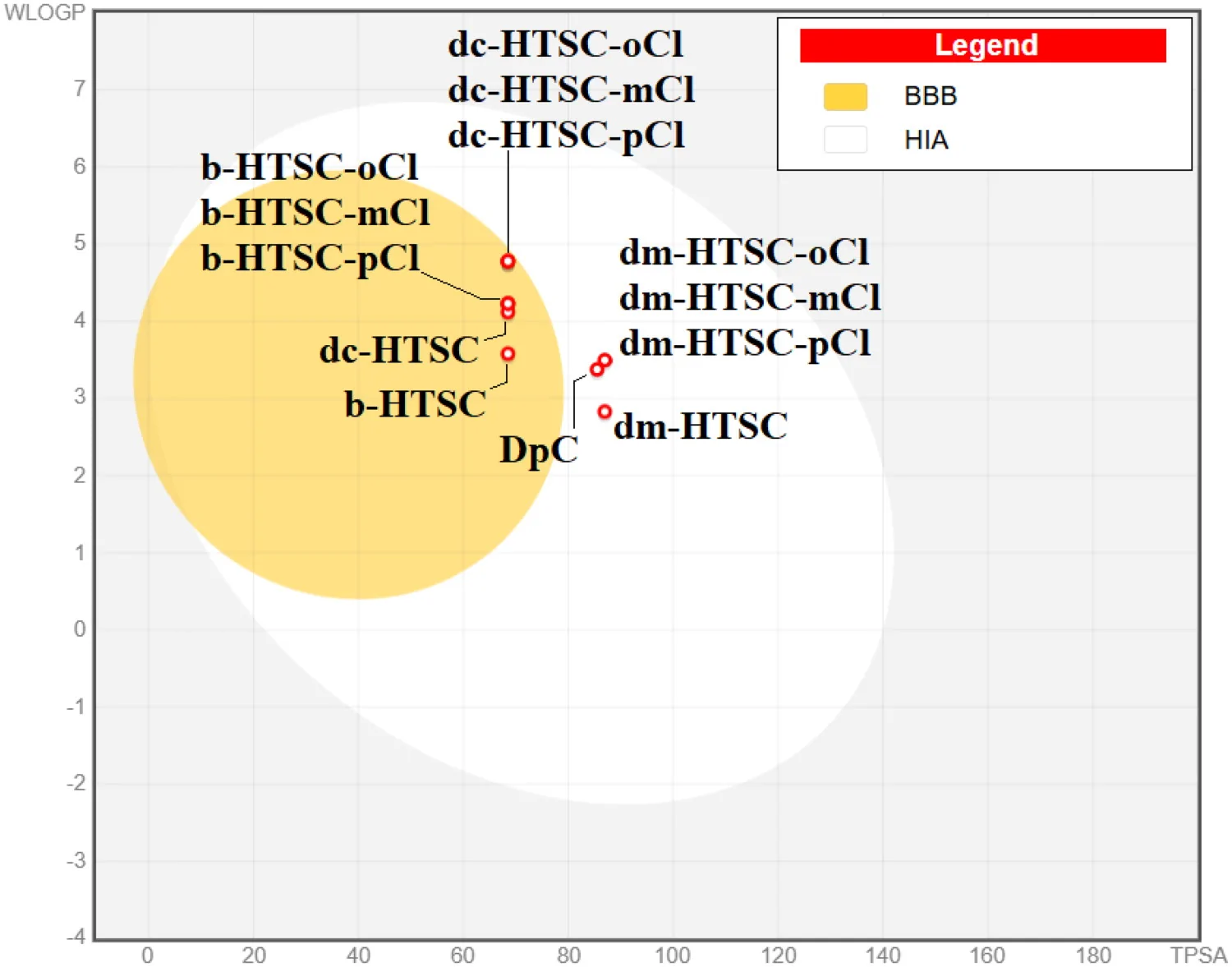
BOILED-Egg diagram generated for the investigated ligands and the DpC benchmark. The white region indicates a high probability of gastrointestinal absorption, whereas the yellow region indicates a high probability of blood–brain barrier permeation according to the SwissADME model.

### Anticancer activity

#### Cytotoxicity evaluation

All twelve ligands and their corresponding Cu(II) and Pd(II) complexes were screened for anticancer activity at 10 µM on SK-MEL-28, A-375, and G-361 cells. For clarity, the initial screening results are depicted in the form of bar graphs (Figures 9–11), while the numerical data can be found in the (Supplementary Materials Table S7). In most cases, free organic ligands exhibited weak or moderate effect at the tested concentration (10 µM). In contrast, several complexes significantly inhibited cell proliferation, especially most of the Cu(II) and Pd(II) complexes bearing 3,4-dimethoxybenzylidene substituents. Additionally, the presence and the position of the chloro- substituent also had a substantial impact on the activity (e.g. in the case of **Cu(dm-TSC)_2_**, **Cu(dm-TSC-oCl)_2_**, **Cu(dm-TSC-mCl)_2_**, and **Cu(dm-TSC-pCl)_2_** on A-375 cells).

**Figure 9.**
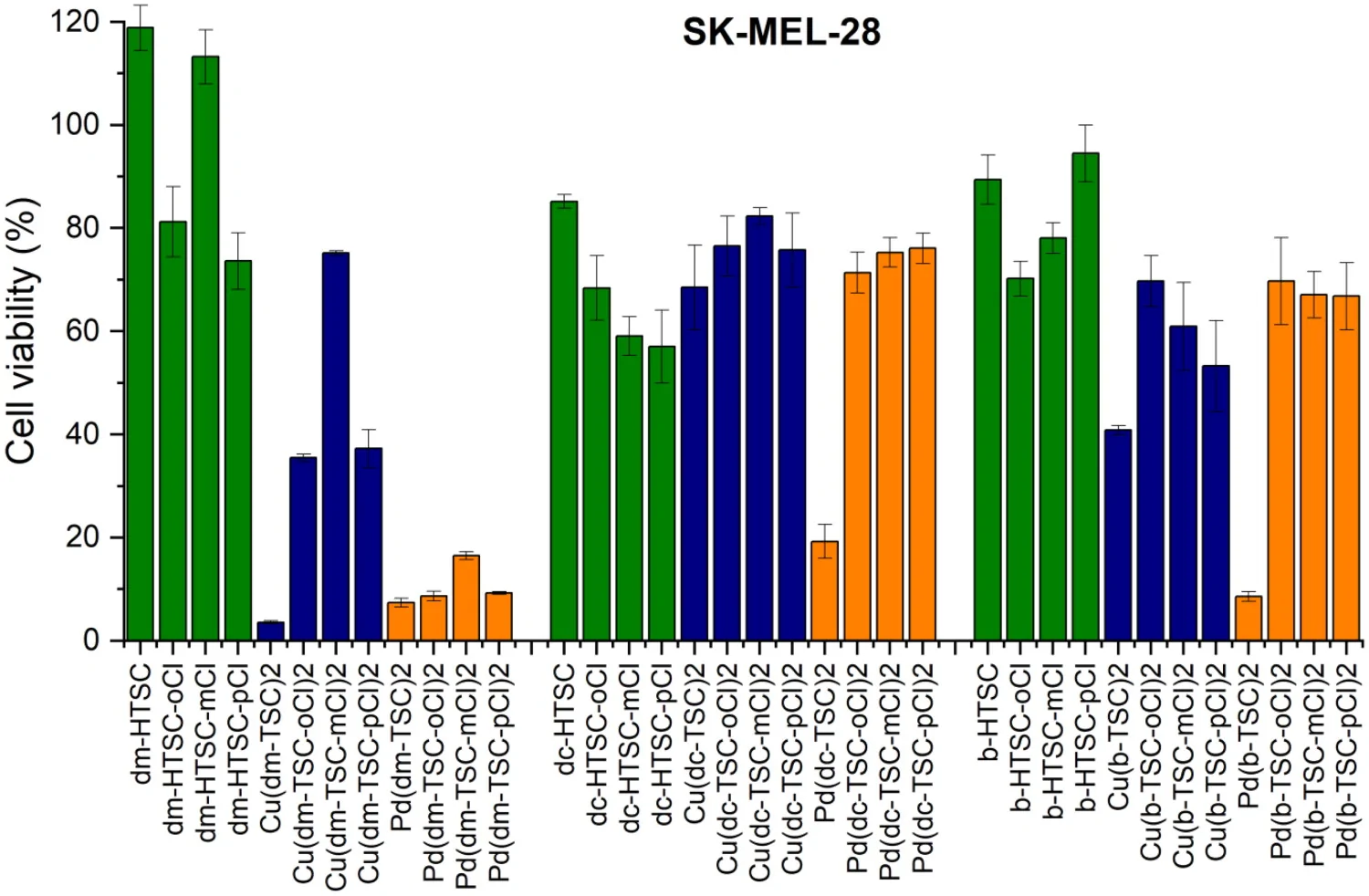
Cell viability of SK-MEL-28 cancer cells after 48 h of incubation with the studied compounds at 10 µM. The three sections represent 3,4-dimethoxybenzylidene-, 3,4-dichlorobenzylidene-, and 4-bromobenzylidene- derivatives, respectively. Green, blue, and orange bars represent free organic ligands, Cu(II) complexes, and Pd(II) complexes, respectively.

**Figure 10.**
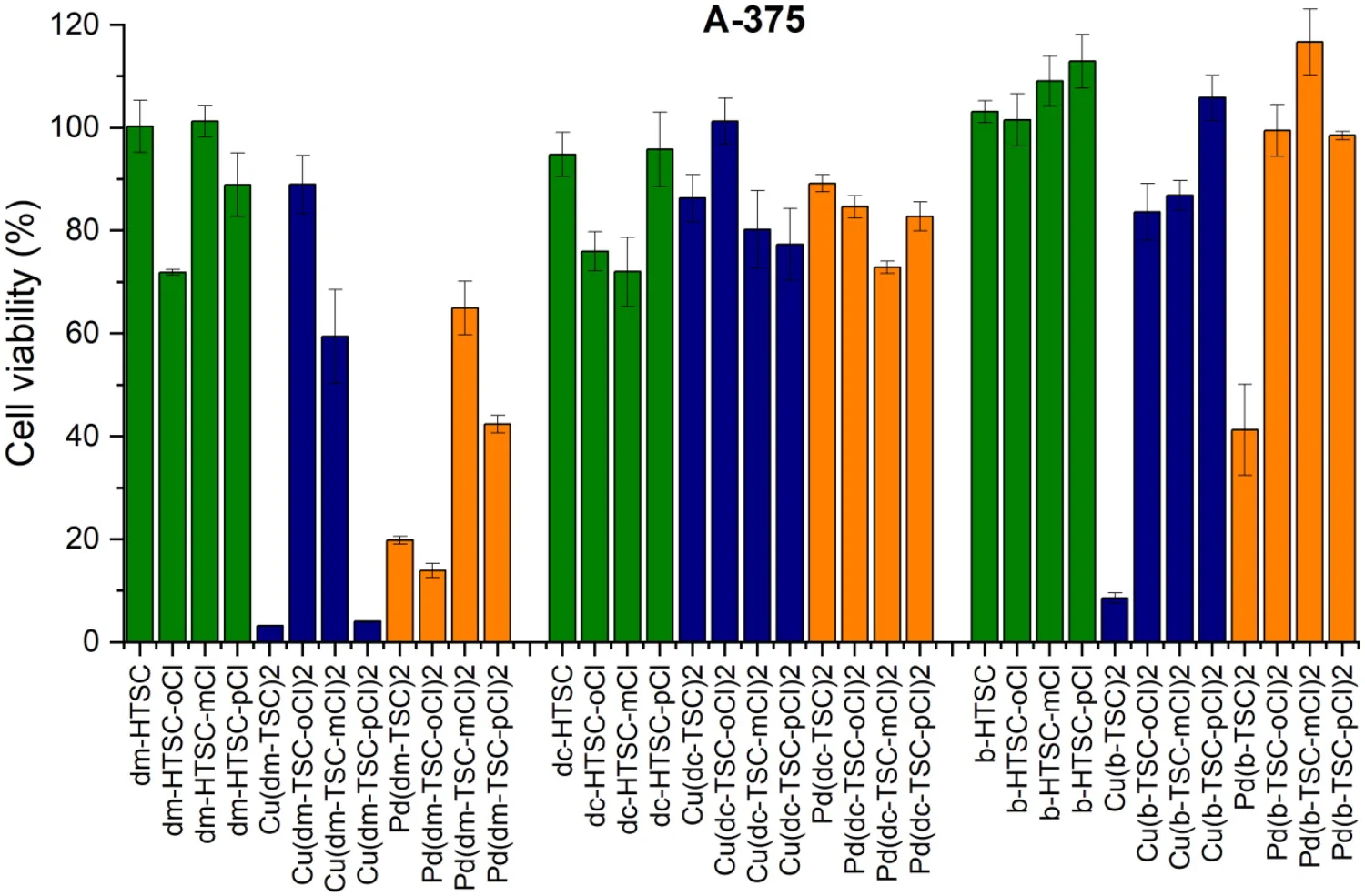
Cell viability of A-375 cancer cells after 48 h of incubation with the studied compounds at 10 µM. The three sections represent 3,4-dimethoxybenzylidene-, 3,4-dichlorobenzylidene-, and 4-bromobenzylidene- derivatives, respectively. Green, blue, and orange bars represent free organic ligands, Cu(II) complexes, and Pd(II) complexes, respectively.

**Figure 11.**
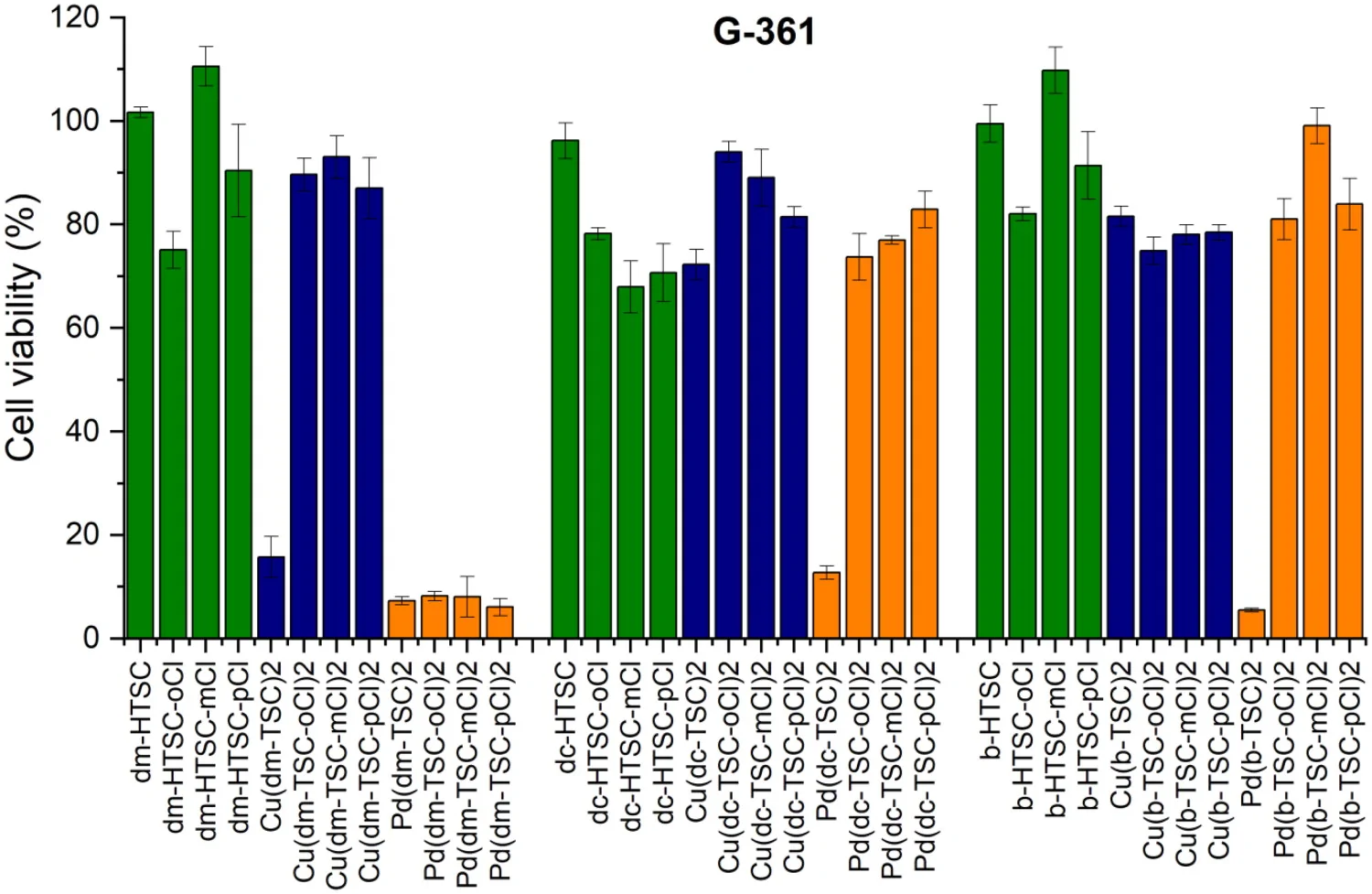
Cell viability of G-361 cancer cells after 48 h of incubation with the studied compounds at 10 µM. The three sections represent 3,4-dimethoxybenzylidene-, 3,4-dichlorobenzylidene-, and 4-bromobenzylidene- derivatives, respectively. Green, blue, and orange bars represent free organic ligands, Cu(II) complexes, and Pd(II) complexes, respectively.

Based on the initial screening results, the most active complexes were selected for IC_50_ determination and for cytotoxicity evaluation on healthy BJ cells:

- among 3,4-dimethoxybenzylidene-substituted complexes: **Cu(dm-TSC)_2_**, **Cu(dm-TSC-oCl)_2_**, **Cu(dm-TSC-pCl)_2_**, **Pd(dm-TSC)_2_**, **Pd(dm-TSC-oCl)_2_**, **Pd(dm-TSC-mCl)_2_**, **Pd(dm-TSC-pCl)_2_**;
- among 3,4-dichlorobenzylidene-substituted complexes: **Pd(dc-TSC)_2_**;
- among 4-bromobenzylidene-substituted complexes: **Cu(b-TSC)_2_**, **Pd(b-TSC)_2_**.

Although **Cu(dm-TSC-mCl)_2_** showed limited activity, it was included for comparison with other 3,4-dimethoxybenzylidene-substituted analogues. The IC_50_ values are presented in Table 1, while Figure S50 presents the cell viability graphs. Several complexes displayed significant activity, reaching sub-micromolar IC_50_ values: **Pd(dm-TSC)_2_** against SK-MEL-28 and G-361 cells, and **Pd(dm-TSC-pCl)_2_** against G-361 cells. While most compounds showed similar cytotoxicity towards cancer cells, their selectivity indexes varied considerably. On this basis, the two most promising complexes were selected for further biological evaluation: **Cu(b-TSC)_2_** on SK-MEL-28 and A-375 (SI = 2.28 and 2.94, respectively); and **Pd(dm-TSC-pCl)_2_** on G-361 (SI = 3.19).

**Table 1.** IC_50_ values determined for the selected complexes.

Compound	IC_50_ ± SD [µM]						
	SK-MEL-28	SI	A-375	SI	G-361	SI	BJ
**Cu(dm-TSC)_2_**	3.58 ± 0.05	1.41	7.48 ± 0.42	0.68	5.04 ± 0.07	1.00	5.06 ± 0.01
**Cu(dm-TSC-oCl)_2_**	5.19 ± 0.19	1.72	>10	nd	>10	nd	8.92 ± 0.94
**Cu(dm-TSC-mCl)_2_**	>10	nd	>10	nd	>10	nd	10.17 ± 0.19
**Cu(dm-TSC-pCl)_2_**	6.74 ± 0.20	1.26	6.94 ± 0.27	1.22	>10	nd	8.46 ± 0.01
**Pd(dm-TSC)_2_**	0.90 ± 0.01	0.98	2.81 ± 0.07	0.31	0.90 ± 0.02	0.98	0.88 ± 0.02
**Pd(dm-TSC-oCl)_2_**	2.05 ± 0.03	1.15	5.54 ± 0.13	0.42	3.77 ± 0.10	0.62	2.35 ± 0.09
**Pd(dm-TSC-mCl)_2_**	2.40 ± 0.09	1.59	>10	nd	1.98 ± 0.04	1.92	3.81 ± 0.11
**Pd(dm-TSC-pCl)_2_**	1.97 ± 0.05	1.04	5.47 ± 0.06	0.37	**0.64 ± 0.00**	**3.19**	2.04 ± 0.04
**Pd(dc-TSC)_2_**	5.47 ± 0.17	0.41	>10	nd	3.49 ± 0.23	0.65	2.27 ± 0.08
**Cu(b-TSC)_2_**	**4.66 ± 0.06**	**2.28**	**4.28 ± 0.12**	**2.94**	>10	nd	10.64 ± 0.29
**Pd(b-TSC)_2_**	1.89 ± 0.09	1.12	9.45 ± 0.05	0.22	8.85 ± 0.44	0.24	2.12 ± 0.05
Vemurafenib	0.51 ± 0.07	nd	4.69 ± 0.29	nd	>10	nd	>10

Selectivity indexes (SI) were calculated as IC_50_(BJ)/IC_50_(cancer cell line). Values for complexes selected for further mechanism of action studies are shown in bold.

*nd – not determined*.

Our previous study on structurally analogous Cu(II) and Pd(II) thiosemicarbazone complexes provided mechanistic insight into their modes of action, indicating that Cu(II) complexes predominantly induce oxidative stress, mitochondrial dysfunction, and glutathione imbalance, whereas Pd(II) complexes exert a more pronounced genotoxic effect, evidenced by γ-H2AX staining and accumulation of AP sites^36^. Building upon these findings, the present study focuses on the downstream biological consequences of treatment, including changes in gene expression, cell-cycle distribution, and apoptosis/necrosis.

#### Gene expression analysis

The effects of the selected compounds - **Cu(b-TSC)_2_** (in SK-MEL-28 and A-375 cell lines) and **Pd(dm-TSC-pCl)_2_** (in the G-361 cell line) - on the expression levels of genes involved in the cell cycle, apoptosis, oxidative stress response, and DNA damage repair were analysed. The results of gene expression analysis are presented in Figure 12. The raw data can be accessed in the (Supplementary Materials Tables S8–S10).

**Figure 12.**
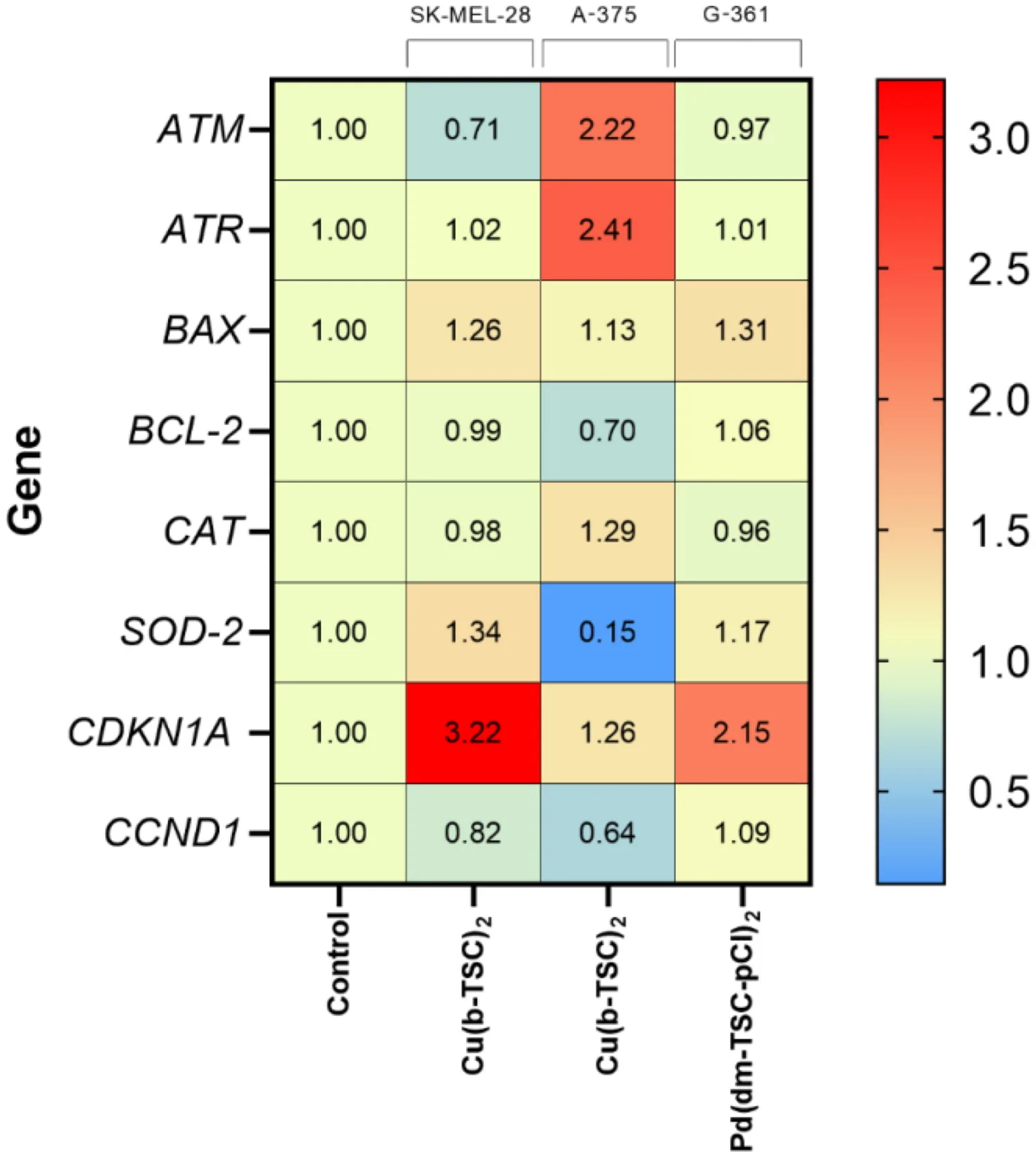
The results of mRNA gene expression analysis in SK-MEL-28 and A-375 cells treated with **Cu(b-TSC)_2_**, and in G-361 cells treated with **Pd(dm-TSC-pCl)_2_** for 48 h at concentrations corresponding to the IC_50_ value. The results are expressed as relative quantification (RQ) values and presented as means. The mean RQ values are illustrated using a colour scale. The control was considered the representative reference sample for relative gene expression analysis. *B-ACT* and *RNA18SN5* were selected as reference genes.

In SK-MEL-28 cells, treatment with **Cu(b-TSC)_2_** led to a significant increase in the expression of the *CDKN1A* gene, which is responsible for cell cycle arrest, and a decrease in the expression of *CCND1*. A similar effect, although slightly weaker, was observed in A-375 cells treated with the same compound, and in the G-361 cells exposed to **Pd(dm-TSC-pCl)_2_**. As discussed in the following section, an increase in the G2/M phase was observed following treatment with the respective compounds in all three melanoma cell lines (Figure 13), confirming these initial findings. In all cell lines, an increase in the expression of the *BAX* gene, which promotes apoptosis, and a decrease in the expression of *BCL-2* were observed after treatment with the compound. Gene expression analysis of *ATM*, a kinase that initiates the response to double-strand DNA breaks, and *ATR*, a kinase that responds to single-strand DNA damage, showed different changes in the tested cell lines exposed to the studied compounds. In SK-MEL-28 cells, *ATM* expression decreased after treatment with **Cu(b-TSC)_2_**, while *ATR* expression remained unchanged compared to the control. In A-375 cells, both *ATM* and *ATR* expression significantly increased after **Cu(b-TSC)_2_** treatment, suggesting activation of cellular pathways involved in the DNA damage response. In G-361 cells, exposure to **Pd(dm-TSC-pCl)_2_** resulted in *ATM* and *ATR* expression remaining at levels similar to the control. A quantitative analysis of the gene expression patterns of *CAT*, an antioxidant enzyme that neutralises hydrogen peroxide, indicated minimal alterations in gene expression in all three melanoma cell lines following exposure to the test compounds. However, a modest tendency towards increased expression was observed in the A-375 cell line following treatment with **Cu(b-TSC)_2_**. Furthermore, the expression of the *SOD-2* gene was not significantly altered, although a slight increase was observed in SK-MEL-28 cells after treatment with **Cu(b-TSC)_2_**, while a significant decrease was observed in A-375 cells after the addition of the same compound. The tested compounds therefore showed limited effects on the expression of important genes related to antioxidant response in the melanoma cell lines tested.

**Figure 13.**
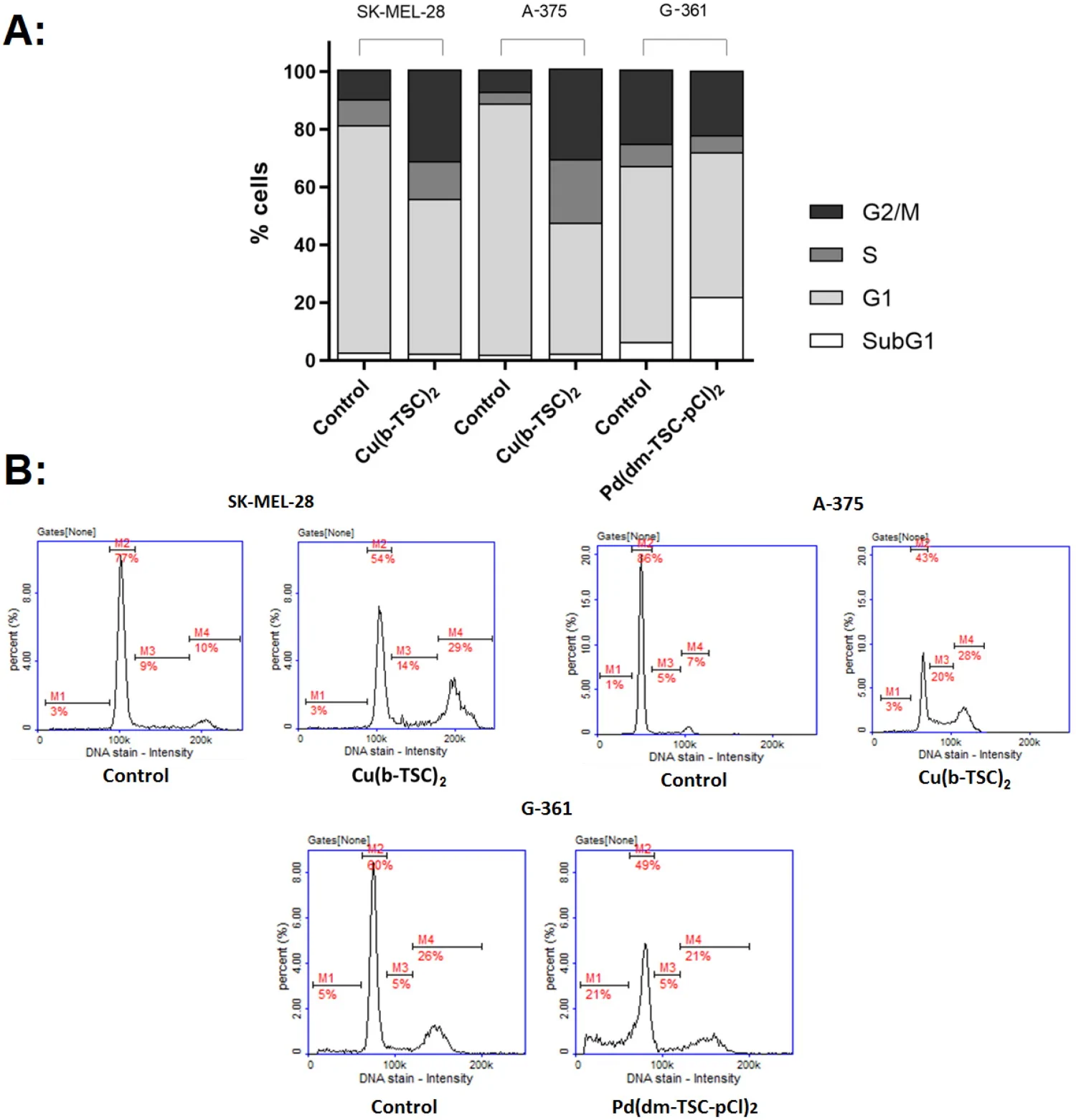
Cell cycle analysis of melanoma cells following treatment with selected complexes, compared to untreated controls. (**A**) Quantitative distribution of cell cycle phases (SubG1, G1, S, and G2/M) in A-375 and SK-MEL-28 cells treated with **Cu(b-TSC)_2_**, and G-361 cells treated with **Pd(dm-TSC-pCl)_2_** at their respective IC_50_ concentrations. (**B**) Representative DNA content histograms for each condition, illustrating the distribution of cell populations in SubG1 (M1), G1 (M2), S (M3), and G2/M (M4) phases; the SubG1 fraction reflects DNA fragmentation associated with apoptosis.

#### Cell cycle analysis

To confirm the differences in the mechanism of action for both complexes suggested by the gene expression results, a cell cycle analysis was performed. In A-375 and SK-MEL-28 melanoma cells treated with **Cu(b-TSC)_2_** at IC_50_ concentrations for 48 h, marked cytostatic changes in the cell cycle were observed compared to untreated control cells (Figure 13). In both cell lines, **Cu(b-TSC)_2_** induced arrest in the G2/M phase. In A-375 cells, a significant increase in the proportion of cells in the S phase (from 5% to 20%) suggests an alteration in cell-cycle progression, potentially involving processes associated with DNA replication. In SK-MEL-28, the effect on S-phase is less pronounced (increase from 9% to 14%), suggesting lower sensitivity. A significant decrease in G1 population in both lines indicates inhibition of proliferation, a desirable effect in the context of anticancer therapy.

In contrast, in the case of G-361 melanoma cells treated with **Pd(dm-TSC-pCl)_2_**, marked cytotoxic changes in the cell cycle were observed compared to untreated cells (Figure 13). A significant increase in sub-G1 (from 5% to 21%) is consistent with substantial apoptotic cell death. The lack of a marked change in the S-phase population suggests that **Pd(dm-TSC-pCl)_2_** does not produce the same pattern of S-phase accumulation observed with **Cu(b-TSC)_2_**. Together with the strong apoptotic response, these findings indicate that **Pd(dm-TSC-pCl)_2_** predominantly promotes cell death rather than pronounced cell-cycle arrest under the conditions tested.

#### Apoptosis detection

Similarly, SK-MEL-28, A-375, and G-361 cells treated with both of the tested compounds at their respective IC_50_ concentrations for 48 h were stained with Annexin-V for apoptosis/necrosis detection. In both cell lines, **Cu(b-TSC)_2_** significantly reduces the percentage of healthy cells (A-375: from 88% to 50%; SK-MEL-28: from 90% to 54%), confirming the compound’s toxicity and impact on cell viability (Figure 14). Both lines show a marked increase in early apoptosis (A-375: from 8% to 33%; SK-MEL-28: from 5% to 31%), indicating that **Cu(b-TSC)_2_** induces programmed cell death. An increase in late apoptosis is observed in both lines, but it is more pronounced in A-375 (from 3% to 16%) than in SK-MEL-28 (from 2% to 8%), suggesting that A-375 progress to apoptosis more rapidly or to a greater extent.

**Figure 14.**
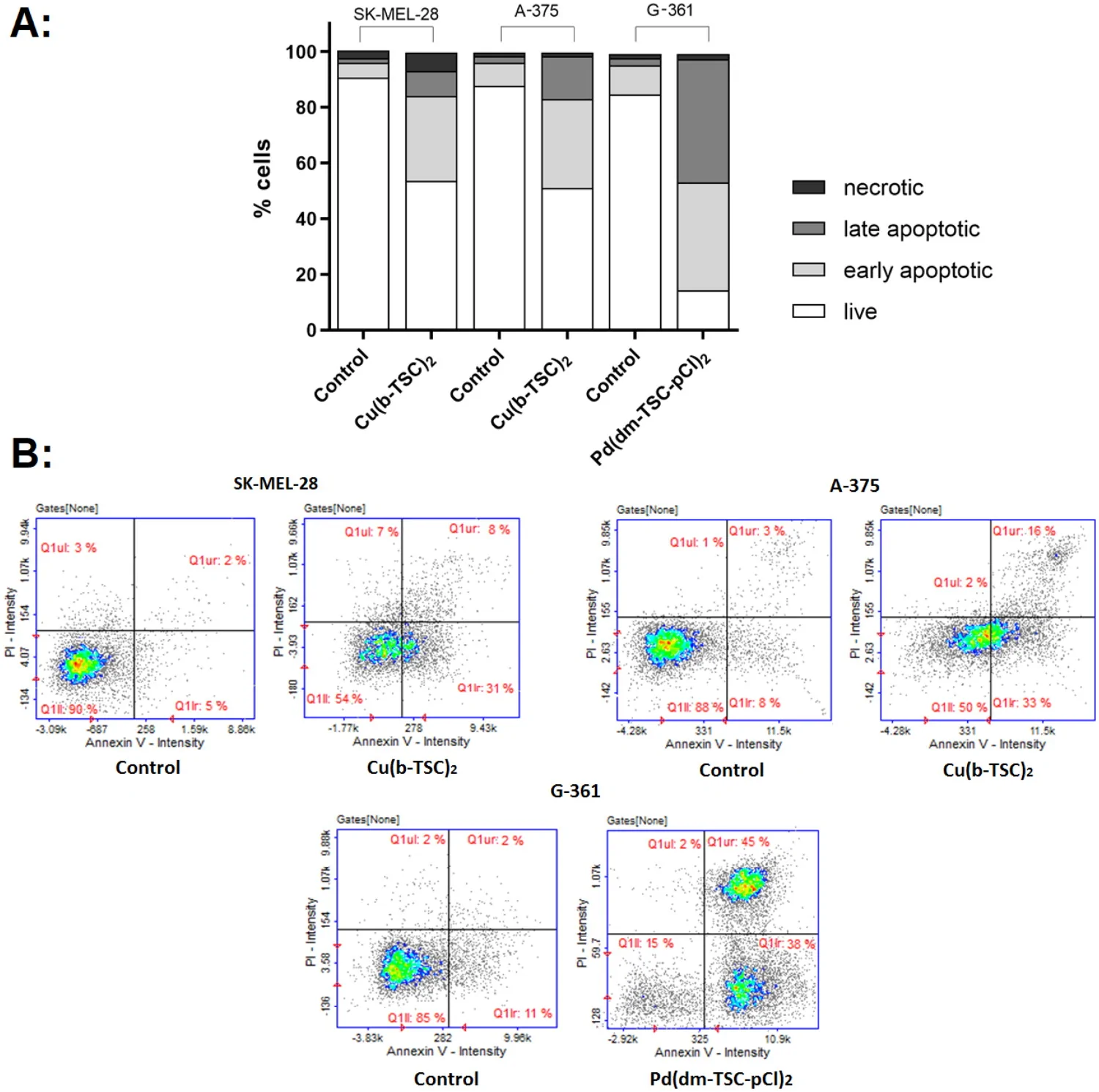
Apoptosis analysis of melanoma cells following treatment with selected complexes, compared to untreated controls. (**A**) Quantitative assessment of A-375 and SK-MEL-28 cells treated with **Cu(b-TSC)_2_**, and G-361 cells treated with **Pd(dm-TSC-pCl)_2_** at their respective IC_50_ concentrations. Cells were stained with Annexin V-CF488A, Hoechst 33342, and propidium iodide (PI) and analysed using the NucleoCounter^®^ NC-3000^™^ image cytometer. The bar graph represents the percentage of live (Annexin V-negative/PI-negative), early apoptotic (Annexin V-positive/PI-negative), late apoptotic (Annexin V-positive/PI-positive), and necrotic cells. **(B)** Representative scatter plots for each condition, illustrating the distribution of cell populations divided into four quadrants: lower left (Q1ll), viable cells; lower right (Q1lr), early apoptotic cells; upper right (Q1ur), late apoptotic cells; and upper left (Q1ul), necrotic cells.

G-361 melanoma cells treated with **Pd(dm-TSC-pCl)_2_** show very high increases in early (38%) and late apoptosis (45%), along with a drastic reduction in healthy cells (from 85% to 15%), confirming the strong apoptotic action of **Pd(dm-TSC-pCl)_2_**(Figure 14). The low proportion of necrotic cells (2%), together with the marked increase in early and late apoptotic populations, is consistent with apoptosis being the predominant mode of cell death under the conditions tested.

#### Activity against vemurafenib-resistant cells

In the last step, the activity of **Cu(b-TSC)_2_** was evaluated in vemurafenib-resistant SK-MEL-28 and A-375 cells, and compared with that of the reference drug (Table 2). As expected, the activity of vemurafenib in cells with induced resistance decreased significantly: nearly 100-fold in SK-MEL-28 and over 5-fold in A-375. At the same time, **Cu(b-TSC)_2_** exhibited comparable activity in both parental and vemurafenib-resistant SK-MEL-28 and A-375 cells. The obtained IC_50_ values suggest that **Cu(b-TSC)_2_** may be a promising candidate for the treatment of vemurafenib-resistant melanomas.

**Table 2.** IC_50_ values determined for **Cu(b-TSC)_2_** and vemurafenib on parental and vemurafenib-resistant SK-MEL-28 and A-375 cells.

Compound	IC_50_ ± SD [µM]			
	SK-MEL-28		A-375	
	parental	vemurafenib-resistant	parental	vemurafenib-resistant
**Cu(b-TSC)_2_**	4.66 ± 0.06	8.41 ± 0.04	4.28 ± 0.12	4.35 ± 0.13
Vemurafenib	0.51 ± 0.07	50.91 ± 0.87	4.69 ± 0.29	24.72 ± 1.79

#### Anticancer activity summary

Based on the biochemical and cellular assays performed in the present study, the observed biological responses show similarities to those previously reported for structurally analogous thiosemicarbazone-based Cu(II) and Pd(II) complexes studied by our group^36^.

The **Cu(b-TSC)_2_** complex exhibited both high anticancer activity and selectivity, particularly against A-375 and SK-MEL-28 cells, which are characterised by high oxidative metabolism and the presence of the BRAF V600E mutation. This mutation leads to constitutive activation of the MAPK pathway, thereby enhancing proliferation and increasing susceptibility to mitochondrial-mediated apoptosis^47^^,^^48^. In the present study, **Cu(b-TSC)_2_** induced G2/M cell-cycle arrest and apoptosis and was associated with changes indicative of a cellular DNA damage response, particularly in A-375 cells. Literature data indicate that Cu^2+^ ions undergo Cu(II)/Cu(I) redox cycling, catalysing Fenton-type reactions, that generate highly reactive hydroxyl radicals (•OH). Cells with elevated oxidative metabolism, such as A-375 and SK-MEL-28, display increased mitochondrial respiratory chain activity, which enhances intracellular H_2_O_2_ availability - a key substrate for Fenton chemistry. In the context of our prior findings demonstrating ROS-mediated cytotoxicity for related Cu(II)-thiosemicarbazone complexes, the increased sensitivity of these melanoma cell lines to **Cu(b-TSC)_2_** is consistent with a contribution of oxidative stress to cellular damage, potentially affecting DNA, proteins, and membrane lipids^36^^,^^49–51^. Cell cycle analysis revealed an increased S-phase population in A-375 cells, and gene expression analysis showed significant upregulation of *ATM* and *ATR*, both of which are key regulators of the DNA damage response. Melanoma cell lines with high oxidative metabolism often show overloaded antioxidant systems (e.g., catalase, SOD). Simultaneously, the study results indicate increased *CAT* and *SOD-2* expression after **Cu(b-TSC)_2_** treatment. Apoptosis analysis in A-375 and SK-MEL-28 cells treated with **Cu(b-TSC)_2_** confirmed activation of the intrinsic apoptotic pathway. Increased *BAX* expression accompanied by decreased *BCL-2* expression indicates a shift towards a pro-apoptotic cellular state and may reflect involvement of mitochondrial apoptotic signalling. Notably, **Cu(b-TSC)_2_** also retained significant activity in vemurafenib-resistant melanoma cell lines, particularly A-375. This finding suggests that the cytotoxic effect of **Cu(b-TSC)_2_** is largely independent of the MAPK pathway and BRAF mutational status, consistent with a mechanism potentially involving mitochondrial dysfunction and/or oxidative stress-related cellular damage rather than targeted kinase inhibition. Consequently, **Cu(b-TSC)_2_** may represent a promising candidate for overcoming resistance to BRAF-targeted therapies.

Interpretation of the biological activity of **Pd(dm-TSC-pCl)_2_** is likewise guided by our earlier mechanistic investigations on closely related Pd(II)-thiosemicarbazone complexes, which demonstrated a predominantly genotoxic mode of action^36^. The **Pd(dm-TSC-pCl)_2_** complex exhibited exceptional activity against G-361 cells (IC_50_ = 0.64 µM) and high selectivity (SI = 3.19). The G-361 melanoma cell line harbours mutations in genes such as *TP53* and *CDKN2A*, resulting in impaired DNA damage sensing and repair capacity^52^. The absence of significant changes in *ATM* and *ATR* expression following **Pd(dm-TSC-pCl)_2_** treatment indicates that these DNA damage response-related transcripts were not markedly altered under the conditions tested. G-361 cells exhibit moderate oxidative metabolism compared to A-375 and SK-MEL-28 cells, which may reduce susceptibility to oxidative stress while increasing vulnerability to DNA-targeting agents, particularly palladium-based complexes^53^. Similar to platinum complexes, Pd(II) complexes may interact with DNA and potentially form covalent DNA adducts, which can interfere with DNA replication and transcription and contribute to apoptotic signalling. The lack of S-phase accumulation and the absence of *ATM* and *ATR* induction in G-361 cells suggest that DNA damage induced by **Pd(dm-TSC-pCl)_2_** may reflect rapid commitment to apoptosis following DNA damage without prolonged cell cycle arrest or activation of repair mechanisms. This interpretation is supported by the strong apoptotic response observed (38% early and 45% late apoptosis), indicating that apoptosis represents a predominant mode of cell death under the conditions tested. These differences in the cellular response to the investigated Pd(II) and Cu(II) complexes are further supported by the distinct proportions of apoptotic and necrotic cells, as well as by differences in the distribution of cells across the Sub-G1 and S phases following treatment with the respective IC_50_ concentrations.

#### Anticancer structure–activity relationship

The observed biological response patterns are presented in Figure 15. While the parent ligands exhibited a limited measure of cytotoxicity, their metal complexes were generally significantly more active. Among complexes, 3,4-diMeO derivatives clearly stand out as the most active, with most of the Pd(II) complexes exhibiting substantial activity, especially against SK-MEL-28 and G-361 cells. A comparable activity was observed for some of the Pd(II) complexes containing 3,4-diCl and 4-Br-substituted ligands, but, interestingly, only in the case of **Pd(dc-TSC)_2_** and **Pd(b-TSC)_2_**, where the chlorine atom was not introduced (**R^1^** = H). A somewhat similar trend was observed for Cu(II) complexes: **Cu(dc-TSC)_2_** and **Cu(b-TSC)_2_**, although they were less active than their Pd(II) analogues. Interestingly, while the presence and the position of the chlorine atom substituent visibly impacted the activity of both Cu(II) and Pd(II) 3,4-diMeO complexes in SK-MEL-28 and A-375, its presence did not significantly influence the activity of Pd(II) 3,4-diMeO complexes in G-361, and even deactivated Cu(II) 3,4-diMeO complexes in G-361. The remaining Cu(II) and Pd(II) complexes, containing 3,4-diCl and 4-Br-substituted ligands and chlorine substituent at **R^1^** exhibited negligible activity.

**Figure 15.**
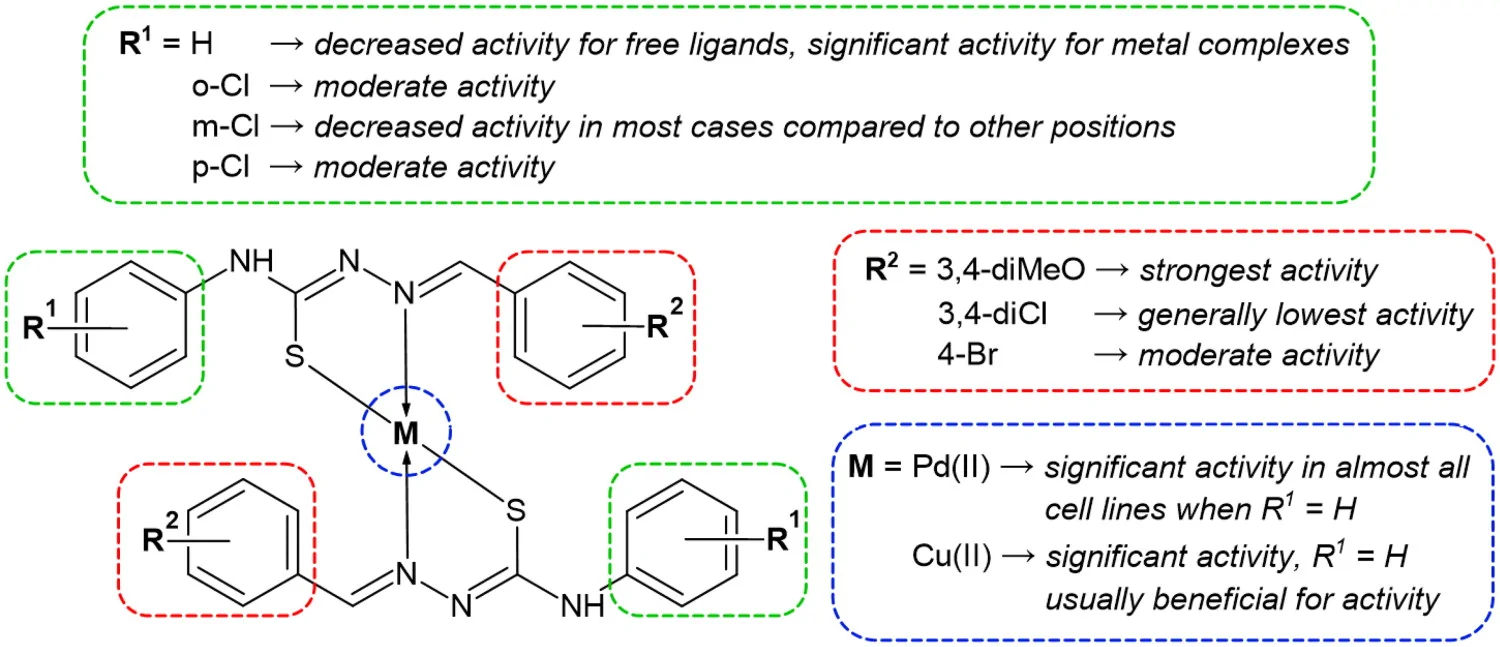
Anticancer structure-activity relationship of the tested compounds.

There are several physicochemical, structural, and biochemical factors governing the observed activity of the investigated compounds. The presence or absence of different substituents directly affects a compound’s key physicochemical properties. Properties such as solubility and lipophilicity are known to be crucial parameters for potential drug candidates. In particular, an appropriate balance between these properties is required for a compound to cross lipid cell membranes while remaining sufficiently soluble. Simultaneously, molecular geometry, protonation/deprotonation equilibria, and electronic and steric effects may influence membrane crossing and interactions with biological targets. In comparison with typical organic molecules, for metal complexes the situation is usually more complex due to additional factors related to coordination geometry, complex stability, speciation, and possible ligand-exchange processes in solution.

Within the studied series, both the metal type and the presence of specific substituents were associated with differences in the observed activity. The Pd(II) complexes generally exhibited lower IC_50_ values than the corresponding Cu(II) complexes, indicating that metal-dependent structural and electronic properties may contribute to the differences in their biological activity. At the same time, cytotoxicity towards non-malignant BJ fibroblasts varied considerably across the series, resulting in substantial differences in selectivity and indicating that higher anticancer potency did not necessarily correspond to improved selectivity. Importantly, the UV-Vis spectra recorded after 24 and 48 h did not show the appearance of new absorption maxima indicative of extensive decomposition or pronounced changes in speciation under the studied conditions. The observed minor shifts in the positions of the absorption maxima may be associated with solvation and/or conformational changes of the ML_2_-type thiosemicarbazone complexes. Nevertheless, subtle changes in speciation or metal-ligand exchange cannot be excluded. In several cases, predominantly for highly halogenated complexes, the spectra recorded after prolonged incubation were characterised by substantially lower absorbance compared with the initial spectra. The character of these changes suggests possible aggregation and/or precipitation.

The general anticancer activity and apparent solution-stability trends within the studied series suggest that the observed activity is strongly influenced by the hydrophilic-lipophilic character of the substituents, although contributions from steric, electronic, and metal-dependent structural effects cannot be excluded. In particular, complexes containing methoxy groups exhibited the highest activity, whereas the introduction of an additional chlorine substituent was associated with decreased activity in almost all cases. This trend suggests that complexes containing more highly halogenated ligands may be excessively hydrophobic under the studied conditions, thereby promoting partial aggregation and/or precipitation.

To further investigate these observations, an exploratory analysis of the relationships between the determined IC_50_ values and four physicochemical descriptors reflecting the hydrophilic-lipophilic balance was performed. These descriptors were calculated *in silico* for all complexes (Table S11):

- logP_o/w_, where P_o/w_ denotes the octanol/water partition coefficient;
- qPSA_NOS_/SASA, representing the fraction of the solvent-accessible surface associated with N, O, and S atoms, where qPSA denotes the quantum-mechanically calculated polar surface area and SASA denotes the total solvent-accessible surface area;
- logS, where S denotes the predicted molar solubility;
- E_solv_, the calculated aqueous solvation energy.

Given the limited validation of such computational descriptors for transition-metal complexes, particularly with regard to their absolute logP_o/w_ and logS values, they were used primarily for relative comparisons within the investigated series. The log(IC_50_) values obtained for **Cu(dm-TSC)_2_**, **Cu(dm-TSC-oCl)_2_**, **Cu(dm-TSC-pCl)_2_**, **Pd(dm-TSC)_2_**, **Pd(dm-TSC-oCl)_2_**, **Pd(dm-TSC-mCl)_2_**, **Pd(dm-TSC-pCl)_2_**, **Pd(dc-TSC)_2_**, **Cu(b-TSC)_2_**, **Pd(b-TSC)_2_** were plotted against the four calculated descriptors (Figures 16 and 17). Accordingly, the resulting trends and R^2^ values were interpreted descriptively rather than as statistically validated correlations.

**Figure 16.**
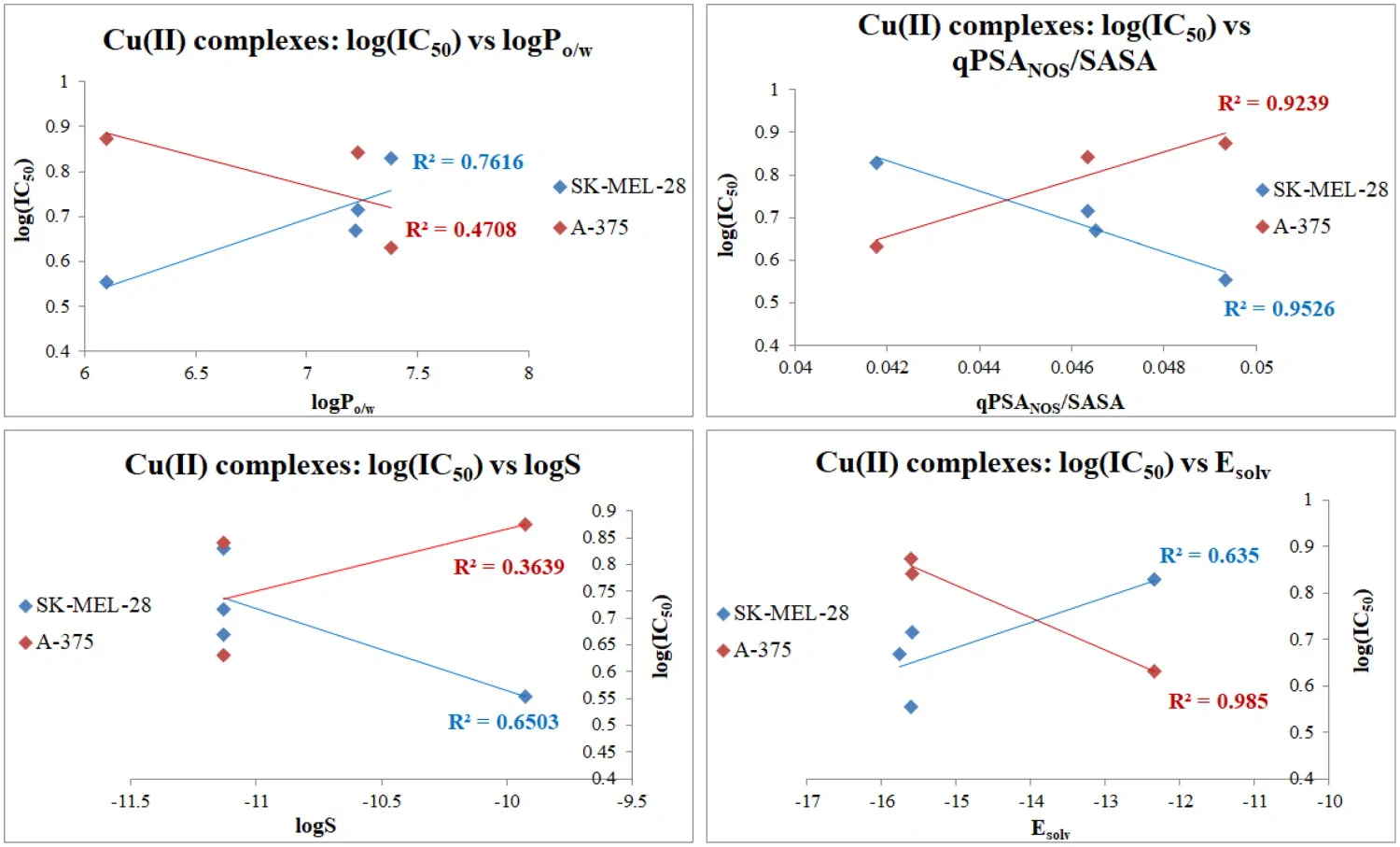
Exploratory plots of log(IC_50_) against the calculated logP_o/w_, qPSA_NOS_/SASA, logS, and E_solv_ descriptors for the investigated Cu(II) complexes. Linear regression lines and the corresponding coefficients of determination (R^2^) are shown.

**Figure 17.**
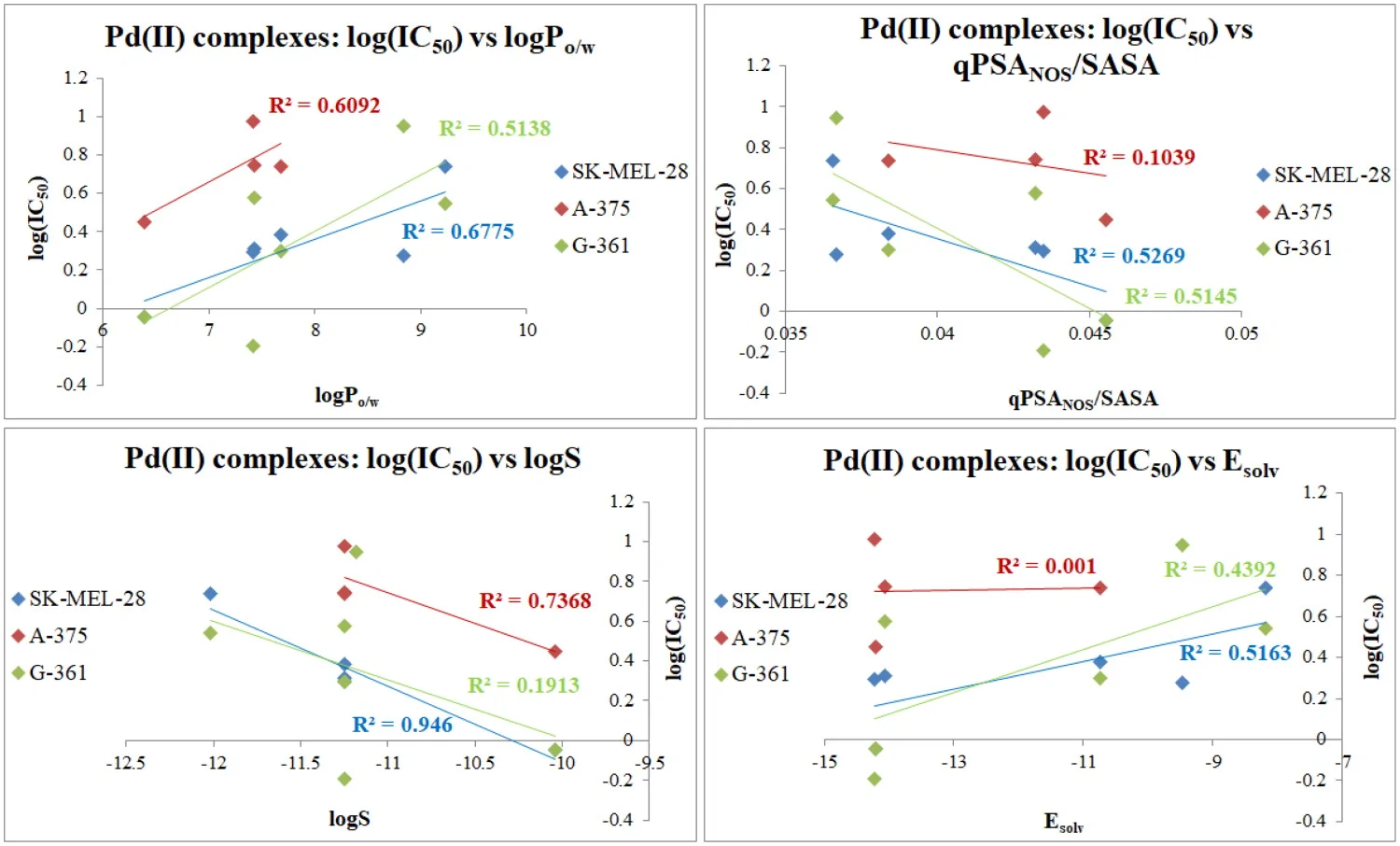
Exploratory plots of log(IC_50_) against the calculated logP_o/w_, qPSA_NOS_/SASA, logS, and E_solv_ descriptors for the investigated Pd(II) complexes. Linear regression lines and the corresponding coefficients of determination (R^2^) are shown.

For the Cu(II) complexes, the exploratory trends appeared to be strongly cell-line-dependent. In the case of activity against SK-MEL-28 cells, greater hydrophilicity and lower lipophilicity were associated with higher activity. The highest R^2^ values were obtained for the qPSA_NOS_/SASA descriptor in both SK-MEL-28 and A-375 cells (R^2^ = 0.9526 and 0.9239, respectively), as well as for E_solv_ in A-375 cells (R^2^ = 0.9850). However, because each regression was based on only three compounds, these high R^2^ values should be interpreted with caution and regarded as preliminary, exploratory trends rather than statistically validated correlations.

In the case of activity against A-375 cells, generally opposite trends were observed. This may emphasise that the relationships identified in this exploratory analysis are cell-line-dependent and should not be generalised across biological models. Insufficient data were available to evaluate possible trends in G-361 cells.

In general, all three tested cell lines showed the same overall pattern for the Pd(II) complexes as that observed for the Cu(II) complexes in SK-MEL-28 cells. Among the analysed compounds, increasing lipophilicity was associated with decreasing activity, with moderate coefficients of determination for logP_o/w_ (R^2^ = 0.5138–0.6775). Conversely, greater hydrophilicity was generally associated with increased activity.

However, weak or negligible relationships were observed for three specific descriptor-cell-line combinations: qPSA_NOS_/SASA in A-375 cells (R^2^ = 0.1039), E_solv_ in A-375 cells (R^2^ = 0.0010), and logS in G-361 cells (R^2^ = 0.1913). Thus, although the Pd(II) complexes showed a more consistent overall pattern than the Cu(II) complexes, the exploratory relationships varied considerably depending on both the descriptor and the cell line.

Taken together, these exploratory observations suggest that the investigated complexes may already possess sufficient lipophilicity for cellular activity, whereas further increases in lipophilicity may be associated with reduced activity, potentially in connection with lower solubility and apparent solution stability. This interpretation is further supported by the categorical distribution shown in Figure 18: compounds exhibiting an IC_50_ below 10 μM in at least one tested cancer cell line were concentrated predominantly in the lower-logP region, whereas most complexes with higher calculated logP values showed no IC_50_ below 10 μM. Although this exploratory pattern does not establish a statistically validated lipophilicity threshold, it highlights the importance of maintaining an appropriate balance between lipophilicity and hydrophilicity. The differences in the trends observed for the Cu(II) and Pd(II) complexes further suggest that the identity of the central metal ion may influence the relationship between physicochemical properties and cellular activity. Overall, these findings highlight the importance of physicochemical factors such as solubility, lipophilicity, and apparent solution stability, which should be considered in the future design of potential thiosemicarbazone-based anticancer agents.

**Figure 18.**
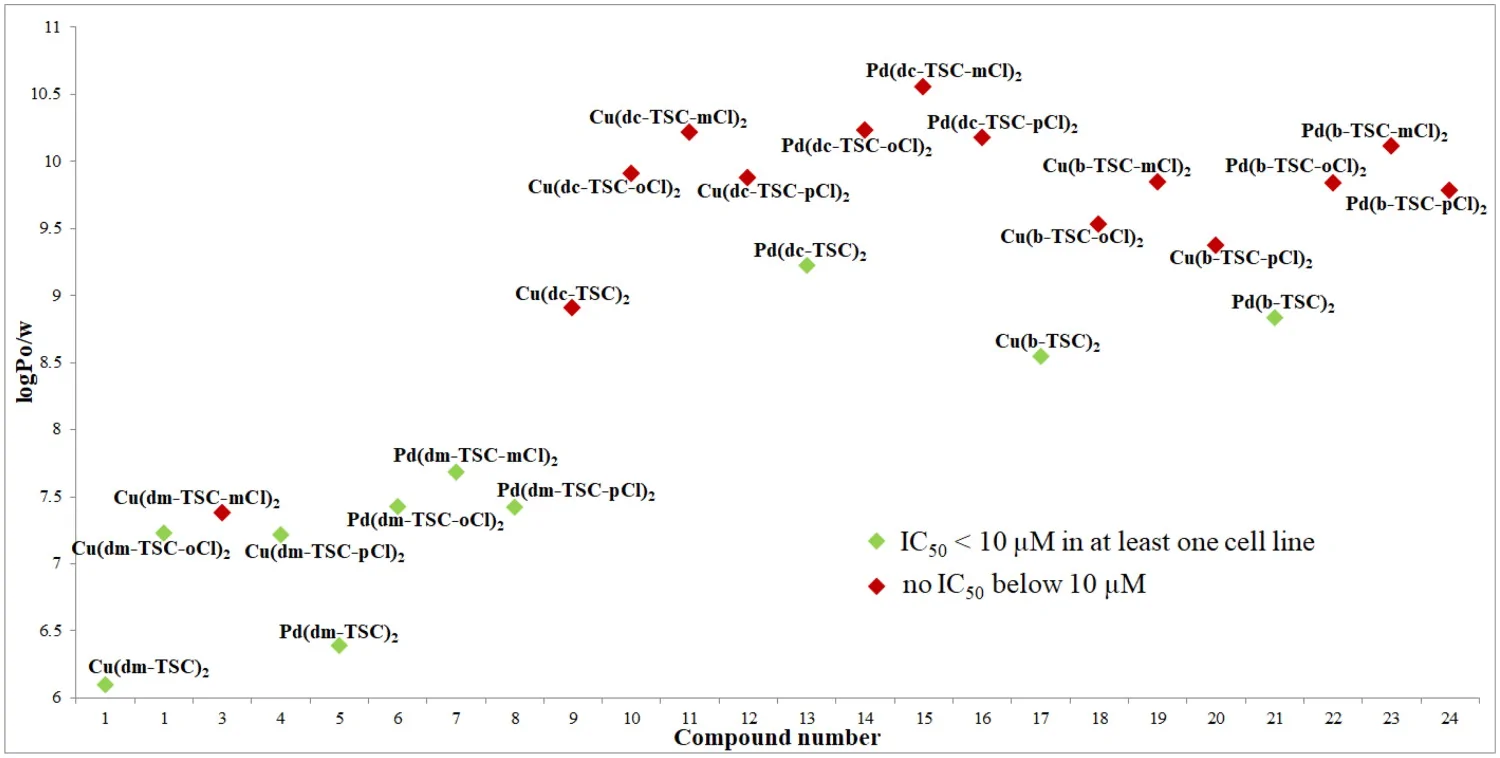
Calculated logP_o/w_ values of the investigated Cu(II) and Pd(II) complexes. Compounds are classified according to whether an IC_50_ value below 10 μM was observed in at least one of the tested cancer cell lines.

### Antibacterial activity

The antibacterial activity of all tested complexes and their parent ligands was evaluated against a panel of bacterial strains using the well-diffusion assay. MIC values (Table 3) were determined using a series of compound dilutions in the agar well diffusion assay. The average inhibition zone diameters obtained for each concentration are presented in Table S12. Among the tested bacteria strains, *E. coli* ATCC 35218 was found to be particularly susceptible, with MIC values as low as 3.125 mg/L. In contrast, *L. monocytogenes* ATCC 15313 and ATCC 35152 were the least sensitive, while *S. aureus* ATCC 25923 and *S. epidermidis* ATCC 12228 exhibited moderate susceptibility.

**Table 3.** Minimum inhibitory concentrations (MICs) of the investigated compounds. Bolded values indicate the lowest MIC observed for each strain.

Compound	MIC [mg/L]				
	*Escherichia* *coli* ATCC 35218	*Listeria* *monocytogenes* ATCC 15313	*Listeria* *monocytogenes* ATCC 35152	*Staphylococcus aureus* ATCC 25923	*Staphylococcus epidermidis* ATCC 12228
**dm-HTSC**	6.25	>100.00	50.00	25.00	25.00
**dm-HTSC-oCl**	12.50	>100.00	>100.00	12.50	100.00
**dm-HTSC-mCl**	6.25	>100.00	>100.00	25.00	50.00
**dm-HTSC-pCl**	**3.125**	>100.00	>100.00	12.50	50.00
**Cu(dm-TSC)_2_**	6.25	>100.00	100.00	12.50	12.50
**Cu(dm-TSC-oCl)_2_**	12.50	>100.00	>100.00	12.50	25.00
**Cu(dm-TSC-mCl)_2_**	6.25	>100.00	100.00	**6.25**	50.00
**Cu(dm-TSC-pCl)_2_**	**3.125**	>100.00	100.00	**6.25**	12.50
**Pd(dm-TSC)_2_**	6.25	>100.00	100.00	25.00	12.50
**Pd(dm-TSC-oCl)_2_**	6.25	>100.00	50.00	12.50	25.00
**Pd(dm-TSC-mCl)_2_**	**3.125**	>100.00	>100.00	25.00	50.00
**Pd(dm-TSC-pCl)_2_**	**3.125**	>100.00	>100.00	12.50	12.50
**dc-HTSC**	6.25	>100.00	>100.00	50.00	50.00
**dc-HTSC-oCl**	**3.125**	>100.00	100.00	50.00	50.00
**dc-HTSC-mCl**	6.25	>100.00	>100.00	50.00	12.50
**dc-HTSC-pCl**	12.50	>100.00	>100.00	50.00	12.50
**Cu(dc-TSC)_2_**	**3.125**	>100.00	>100.00	12.50	25.00
**Cu(dc-TSC-oCl)_2_**	**3.125**	>100.00	50.00	50.00	25.00
**Cu(dc-TSC-mCl)_2_**	**3.125**	>100.00	25.00	25.00	12.50
**Cu(dc-TSC-pCl)_2_**	6.25	>100.00	100.00	25.00	12.50
**Pd(dc-TSC)_2_**	**3.125**	>100.00	100.00	12.50	25.00
**Pd(dc-TSC-oCl)_2_**	6.25	>100.00	>100.00	50.00	12.50
**Pd(dc-TSC-mCl)_2_**	**3.125**	>100.00	>100.00	50.00	12.50
**Pd(dc-TSC-pCl)_2_**	6.25	>100.00	100.00	25.00	25.00
**b-HTSC**	12.50	100.00	100.00	25.00	50.00
**b-HTSC-oCl**	12.50	>100.00	100.00	25.00	25.00
**b-HTSC-mCl**	6.25	>100.00	100.00	50.00	12.50
**b-HTSC-pCl**	12.50	>100.00	>100.00	50.00	25.00
**Cu(b-TSC)_2_**	6.25	**25.00**	**12.50**	**6.25**	12.50
**Cu(b-TSC-oCl)_2_**	25.00	>100.00	100.00	12.50	**6.25**
**Cu(b-TSC-mCl)_2_**	25.00	>100.00	100.00	25.00	12.50
**Cu(b-TSC-pCl)_2_**	12.50	>100.00	>100.00	12.50	12.50
**Pd(b-TSC)_2_**	12.50	100.00	100.00	25.00	12.50
**Pd(b-TSC-oCl)_2_**	25.00	>100.00	50.00	25.00	12.50
**Pd(b-TSC-mCl)_2_**	6.25	>100.00	50.00	50.00	100.00
**Pd(b-TSC-pCl)_2_**	25.00	>100.00	>100.00	50.00	12.50
Gen/Van*	1.00	1.00	1.00	1.00	1.00

* Reference antibacterial agents: Gentamicin (Gen) for *E. coli*; Vancomycin (Van) for *L. monocytogenes*, *S. aureus*, *S. epidermidis*.

In contrast to the anticancer activity, the antimicrobial activity of the tested compounds appeared to be significantly less influenced by structural modifications and metal complexation, and was more distinctly strain-dependent. *E. coli* was consistently the most susceptible microorganism, whereas both *L. monocytogenes* strains were markedly less sensitive. Differences in susceptibility among bacterial species may arise from variations in cell envelope composition, membrane permeability, and intracellular uptake or accumulation of the compounds. In this context, several 3,4-dimethoxybenzylidene- and 3,4-dichlorobenzylidene-substituted ligands, along with their Cu(II) or Pd(II) complexes, exhibited the lowest MIC values (3.125 mg/L on *E. coli*), compared to 1.00 mg/L for a reference antibiotic gentamicin. Conversely, the corresponding 4-bromobenzylidene-containing derivatives showed slightly reduced activity. A somewhat opposite trend was observed for *S. epidermidis*, which was most susceptible to complexes bearing 4-bromobenzylidene-subtituted ligands, particularly those containing Cu(II) ions. Overall, Cu(II) complexes exhibited superior antibacterial activity compared to both the free parent ligands and their Pd(II) analogues. Coordination to Cu(II) may alter the physicochemical properties of the ligands, including lipophilicity and membrane permeability, thereby affecting their interactions with bacterial cells. The higher activity of Cu(II) complexes may be related to differences in redox properties, coordination behaviour, and interactions with bacterial biomolecules compared to the more redox-inert Pd(II) analogues. Notably, **Cu(b-TSC)_2_** emerged as the most versatile compound, as it was the only compound to exhibit considerable activity against all five tested strains, including both *L. monocytogenes* strains, which in general were the most tolerant. Nevertheless, because antibacterial activity was assessed using an agar diffusion-based assay, diffusion behaviour may also contribute to the observed differences, and therefore the proposed structure–activity relationships should be interpreted with caution.

## Conclusions

Twenty-four new Cu(II) and Pd(II) complexes based on substituted thiosemicarbazone ligands were synthesised, characterised, and evaluated for their anticancer and antibacterial activities. Single-crystal X-ray diffraction analysis confirmed ligand deprotonation and the formation of neutral ML_2_-type complexes.

Although the parent ligands exhibited only weak cytotoxicity, several complexes, particularly those containing 3,4-dimethoxybenzylidene and 4-bromobenzylidene moieties, showed substantial activity against melanoma cells. **Cu(b-TSC)_2_** and **Pd(dm-TSC-pCl)_2_** emerged as the most promising compounds, displaying high potency and selectivity towards SK-MEL-28 and A-375 cells, and G-361 cells, respectively. Mechanistic studies showed that both complexes induced cell-cycle arrest and activated pro-apoptotic genes. Notably, **Cu(b-TSC)_2_** retained its activity against vemurafenib-resistant SK-MEL-28 and A-375 cells. Although the selectivity indices were moderate, they varied considerably depending on the metal centre and cell line, suggesting that cellular metabolism, redox status, and DNA-damage response may influence sensitivity to the complexes. The compounds selected for further investigation showed improved selectivity towards particular melanoma models, with SI values of 2.28 and 2.94 for **Cu(b-TSC)_2_** in SK-MEL-28 and A-375 cells, respectively, and 3.19 for **Pd(dm-TSC-pCl)_2_** in G-361 cells.

The SAR analysis revealed that additional chloro substitution on the N4-bound phenyl ring generally reduced anticancer activity, while complexes containing 3,4-dimethoxybenzylidene ligands displayed the most favourable overall activity profile. These trends were consistent with the calculated physicochemical descriptors and the apparent solution-stability results. More highly chlorinated complexes generally exhibited greater calculated lipophilicity and a more pronounced time-dependent decrease in UV-Vis absorbance, consistent with an increased tendency towards aggregation and/or precipitation. Thus, the results indicate that increasing lipophilicity beyond a certain range may become unfavourable by reducing aqueous solubility and the concentration of dissolved complex available for biological interactions. Although the descriptor-activity relationships should be regarded as exploratory, they emphasise that an appropriate balance among lipophilicity, solubility, and apparent solution stability is more important than maximising lipophilicity alone. This structure-property relationship provides a useful direction for the future optimisation of thiosemicarbazone-based anticancer metal complexes.

In contrast to the anticancer results, antibacterial activity was less dependent on structural modifications and more strongly influenced by the bacterial strain. Most complexes and their parent ligands exhibited MIC values of 3.125–25.00 mg/L against *E. coli*, whereas activity against *S. aureus* and *S. epidermidis* was generally moderate and was lowest against both *L. monocytogenes* strains. Interestingly, **Cu(b-TSC)_2_** was the only compound active against all tested strains, with MIC values ranging from 6.25 to 25.00 mg/L. Overall, several ligands and their metal complexes exhibited low-mg/L MIC values, identifying them as promising starting points for the development of compounds targeting selected bacterial strains.

## Experimental

### Synthesis of the ligands

The parent ligands were synthesised and characterised according to procedures previously reported by our group^54^ and by Serda et al. ^55^. Therefore, detailed spectral and physicochemical characterisation of the free ligands is not repeated here and can be found in the cited references. To our best knowledge, the crystal structures of **dm-HTSC**, **dm-HTSC-oCl**, **dm-HTSC-mCl**, **dm-HTSC-pCl**, and **b-HTSC** are reported here for the first time.

### Synthesis of the complexes

All twenty-four Cu(II) and Pd(II) complexes were obtained in very good or good yields, following the general procedure recently reported by our group^36^. 0.20 mmol of an appropriate organic ligand was dissolved in 3 ml of DMSO, and an equimolar amount (0.20 mmol) of an appropriate salt was added. For the synthesis of Cu(II) complexes, an aqueous solution of copper(II) chloride was used; for Pd(II) complexes, a solution of palladium(II) chloride in diluted HCl was used. After 1 h of stirring at room temperature, an excess of cold distilled water was added in each case. The resulting brown precipitates of Cu(II) complexes and orange precipitates of Pd(II) complexes were filtered, washed several times with distilled water, dried, and analysed. Later, several attempts were made to recrystallize the compounds using various methods described in the literature^56^. **Cu(dm-TSC)_2_** and **Cu(dm-TSC-oCl)_2_** were recrystallized from ACN; **Cu(b-TSC)_2_** was recrystallized from DMSO; **Pd(dm-TSC)_2_**, **Pd(dm-TSC-oCl)_2_**, **Pd(dm-TSC-pCl)_2_**, **Pd(dc-TSC)_2_**, **Pd(b-TSC)_2_**, and **Pd(b-TSC-pCl)_2_** were recrystallized from DMF/MeOH (*v/v* = 1:1) mixture. Despite repeated crystallisation attempts using the solvents and solvent mixtures described above, single crystals of the remaining complexes suitable for X-ray diffraction analysis could not be obtained.

#### Complexes based on 3,4-dimethoxybenzylidene-substituted ligands

**Cu(dm-TSC)_2_** (C_32_H_32_CuN_6_O_4_S_2_) (692.31 g/mol), yield (86%). Anal. calculated (%): Cu, 9.18; S, 9.26. Found (%): Cu, 9.77; S, 9.12. **HRMS** (ESI/Q-TOF) *m/z*: calculated for [C_32_H_32_CuN_6_O_4_S_2_ + H]^+^: 692.1295. Found: 692.1303. **FTIR** (KBr, cm^−1^): *ν*(NH): 3412; *ν*(CH): 3126–2835; *ν*(C = N), *ν*(CC), *δ*(CH), *δ*(NH): 1598, 1555, 1508, 1463, 1450, 1420, 1333, 1316, 1268; *ν*(CS), *ν*(C-N), *ν*(NN): 1243, 1200, 1164, 1141, 1074. **UV-Vis** (H_2_O/DMSO 50%/50%; λ_max_, nm): = 350.

**Cu(dm-TSC-oCl)_2_** (C_32_H_30_Cl_2_CuN_6_O_4_S_2_) (761.20 g/mol), yield (88%). Anal. calculated (%): Cu, 8.35; S, 8.42. Found (%): Cu, 7.86; S, 8.63. **HRMS** (ESI/Q-TOF) *m/z*: calculated for [C_32_H_30_Cl_2_CuN_6_O_4_S_2_ + H]^+^: 760.0516. Found: 760.0524. **FTIR** (KBr, cm^−1^): *ν*(NH): 3398; 3255; *ν*(CH): 3114–2833; *ν*(C = N), *ν*(CC), *δ*(CH), *δ*(NH): 1595, 1555, 1508, 1463, 1437, 1420, 1268; *ν*(CS), *ν*(C-N), *ν*(NN): 1242, 1198, 1162, 1139, 1075. **UV-Vis** (H_2_O/DMSO 50%/50%; λ_max_, nm): = 336.

**Cu(dm-TSC-mCl)_2_** (C_32_H_30_Cl_2_CuN_6_O_4_S_2_) (761.20 g/mol), yield (75%). Anal. calculated (%): Cu, 8.35; S, 8.42. Found (%): Cu, 9.05; S, 8.84. **HRMS** (ESI/Q-TOF) *m/z*: calculated for [C_32_H_30_Cl_2_CuN_6_O_4_S_2_ + H]^+^: 760.0516. Found: 760.0526. **FTIR** (KBr, cm^−1^): *ν*(NH): 3404, 3314; *ν*(CH): 3118–2835; *ν*(C = N), *ν*(CC), *δ*(CH), *δ*(NH): 1595, 1550, 1508, 1479, 1438, 1420, 1335, 1270; *ν*(CS), *ν*(C-N), *ν*(NN): 1243, 1198, 1164, 1148, 1098, 1074. **UV-Vis** (H_2_O/DMSO 50%/50%; λ_max_, nm): = 352.

**Cu(dm-TSC-pCl)_2_** (C_32_H_30_Cl_2_CuN_6_O_4_S_2_) (761.20 g/mol), yield (86%). Anal. calculated (%): Cu, 8.35; S, 8.42. Found (%): Cu, 9.07; S, 9.08. **HRMS** (ESI/Q-TOF) *m/z*: calculated for [C_32_H_30_Cl_2_CuN_6_O_4_S_2_ + H]^+^: 760.0516. Found: 760.0524. **FTIR** (KBr, cm^−1^): *ν*(NH): 3253; *ν*(CH): 3114–2835; *ν*(C = N), *ν*(CC), *δ*(CH), *δ*(NH): 1595, 1540, 1508, 1490, 1463, 1423, 1396, 1308, 1269; *ν*(CS), *ν*(C-N), *ν*(NN): 1243, 1198, 1164, 1148, 1090. **UV-Vis** (H_2_O/DMSO 50%/50%; λ_max_, nm): = 329.

**Pd(dm-TSC)_2_** (C_32_H_32_N_6_O_4_PdS_2_) (735.18 g/mol), yield (85%). Anal. calculated (%): Pd, 14.48; S, 8.72. Found (%): Pd, 14.87; S, 9.43. **HRMS** (ESI/Q-TOF) *m/z*: calculated for [C_32_H_32_N_6_O_4_PdS_2_ + H]^+^: 735.1034. Found: 735.1049. **FTIR** (KBr, cm^−1^): *ν*(NH): 3320; *ν*(CH): 3057–2835; *ν*(C = N), *ν*(CC), *δ*(CH), *δ*(NH): 1596, 1509, 1438, 1420, 1314, 1268; *ν*(CS), *ν*(C-N), *ν*(NN): 1204, 1164, 1139, 1080. **UV-Vis** (H_2_O/DMSO 50%/50%; λ_max_, nm): = 346.

**Pd(dm-TSC-oCl)_2_** (C_32_H_30_Cl_2_N_6_O_4_PdS_2_) (804.07 g/mol), yield (81%). Anal. calculated (%): Pd, 13.23; S, 7.98. Found (%): Pd, 13.43; S, 8.35. **HRMS** (ESI/Q-TOF) *m/z*: calculated for [C_32_H_30_Cl_2_N_6_O_4_PdS_2_ + H]^+^: 803.0255. Found: 803.0258. **FTIR** (KBr, cm^−1^): *ν*(NH): 3396, 3262; *ν*(CH): 3076–2834; *ν*(C = N), *ν*(CC), *δ*(CH), *δ*(NH): 1593, 1577, 1541, 1510, 1463, 1437, 1420, 1268; *ν*(CS), *ν*(C-N), *ν*(NN): 1242, 1202, 1163, 1138. **UV-Vis** (H_2_O/DMSO 50%/50%; λ_max_, nm): = 336.

**Pd(dm-TSC-mCl)_2_** (C_32_H_30_Cl_2_N_6_O_4_PdS_2_) (804.07 g/mol), yield (85%). Anal. calculated (%): Pd, 13.23; S, 7.98. Found (%): Pd, 13.22; S, 8.11. **HRMS** (ESI/Q-TOF) *m/z*: calculated for [C_32_H_30_Cl_2_N_6_O_4_PdS_2_ + H]^+^: 803.0255. Found: 803.0266. **FTIR** (KBr, cm^−1^): *ν*(NH): 3318; *ν*(CH): 3077–2835; *ν*(C = N), *ν*(CC), *δ*(CH), *δ*(NH): 1594, 1550, 1509, 1479, 1439, 1420, 1270; *ν*(CS), *ν*(C-N), *ν*(NN): 1243, 1203, 1163, 1138, 1099, 1076. **UV-Vis** (H_2_O/DMSO 50%/50%; λ_max_, nm): = 338.

**Pd(dm-TSC-pCl)_2_** (C_32_H_30_Cl_2_N_6_O_4_PdS_2_) (804.07 g/mol), yield (83%). Anal. calculated (%): Pd, 13.23; S, 7.98. Found (%): Pd, 13.81; S, 8.46. **HRMS** (ESI/Q-TOF) *m/z*: calculated for [C_32_H_30_Cl_2_N_6_O_4_PdS_2_ + H]^+^: 803.0255. Found: 803.0262. **FTIR** (KBr, cm^−1^): *ν*(NH): 3394, 3331; *ν*(CH): 2999–2835; *ν*(C = N), *ν*(CC), *δ*(CH), *δ*(NH): 1594, 1508, 1491, 1463, 1420, 1397, 1308, 1271; *ν*(CS), *ν*(C-N), *ν*(NN): 1245, 1162, 1139, 1091. **UV-Vis** (H_2_O/DMSO 50%/50%; λ_max_, nm): = 344.

#### Complexes based on 3,4-dichlorobenzylidene-substituted ligands

**Cu(dc-TSC)_2_** (C_28_H_20_Cl_4_CuN_6_S_2_) (709.99 g/mol), yield (79%). Anal. calculated (%): Cu, 8.95; S, 9.03. Found (%): Cu, 8.94; S, 9.17. **HRMS** (ESI/Q-TOF) *m/z*: calculated for [C_28_H_20_Cl_4_CuN_6_S_2_ + H]^+^: 707.9314. Found: 707.9304. **FTIR** (KBr, cm^−1^): *ν*(NH): 3325; *ν*(CH): 3117–2977; *ν*(C = N), *ν*(CC), *δ*(CH), *δ*(NH): 1596, 1551, 1508, 1497, 1474, 1449, 1317, 1274; *ν*(CS), *ν*(C-N), *ν*(NN): 1219, 1198, 1131, 1083. **UV-Vis** (H_2_O/DMSO 50%/50%; λ_max_, nm): = 341.

**Cu(dc-TSC-oCl)_2_** (C_28_H_18_Cl_6_CuN_6_S_2_) (778.88 g/mol), yield (90%). Anal. calculated (%): Cu, 8.16; S, 8.23. Found (%): Cu, 8.34; S, 8.91. **HRMS** (ESI/Q-TOF) *m/z*: calculated for [C_28_H_18_Cl_6_CuN_6_S_2_ + H]^+^: 775.8534. Found: 775.8536. **FTIR** (KBr, cm^−1^): *ν*(NH): 3311, 3259; *ν*(CH): 3096–2948; *ν*(C = N), *ν*(CC), *δ*(CH), *δ*(NH): 1593, 1551, 1513, 1472, 1444, 1400, 1326, 1275; *ν*(CS), *ν*(C-N), *ν*(NN): 1218, 1198, 1129, 1084. **UV-Vis** (H_2_O/DMSO 50%/50%; λ_max_, nm): = 335.

**Cu(dc-TSC-mCl)_2_** (C_28_H_18_Cl_6_CuN_6_S_2_) (778.88 g/mol), yield (91%). Anal. calculated (%): Cu, 8.16; S, 8.23. Found (%): Cu, 8.40; S, 8.24. **HRMS** (ESI/Q-TOF) *m/z*: calculated for [C_28_H_18_Cl_6_CuN_6_S_2_ + H]^+^: 775.8534. Found: 775.8524. **FTIR** (KBr, cm^−1^): *ν*(NH): 3390, 3267; *ν*(CH): 3098–2949; *ν*(C = N), *ν*(CC), *δ*(CH), *δ*(NH): 1592, 1550, 1490, 1473, 1444, 1433, 1399, 1310, 1277; *ν*(CS), *ν*(C-N), *ν*(NN): 1246, 1217, 1199, 1129, 1084. **UV-Vis** (H_2_O/DMSO 50%/50%; λ_max_, nm): = 349.

**Cu(dc-TSC-pCl)_2_** (C_28_H_18_Cl_6_CuN_6_S_2_) (778.88 g/mol), yield (87%). Anal. calculated (%): Cu, 8.16; S, 8.23. Found (%): Cu, 8.81; S, 8.92. **HRMS** (ESI/Q-TOF) *m/z*: calculated for [C_28_H_18_Cl_6_CuN_6_S_2_ + H]^+^: 775.8534. Found: 775.8528. **FTIR** (KBr, cm^−1^): *ν*(NH): 3391, 3267; *ν*(CH): 3099–2949; *ν*(C = N), *ν*(CC), *δ*(CH), *δ*(NH): 1592, 1549, 1490, 1473, 1444, 1433, 1398, 1310, 1277; *ν*(CS), *ν*(C-N), *ν*(NN): 1246, 1217, 1199, 1129, 1084. **UV-Vis** (H_2_O/DMSO 50%/50%; λ_max_, nm): = 336.

**Pd(dc-TSC)_2_** (C_28_H_20_Cl_4_N_6_PdS_2_) (752.86 g/mol), yield (88%). Anal. calculated (%): Pd, 14.14; S, 8.52. Found (%): Pd, 14.49; S, 8.92. **HRMS** (ESI/Q-TOF) *m/z*: calculated for [C_28_H_20_Cl_4_N_6_PdS_2_ + H]^+^: 750.9053. Found: 750.9064. **FTIR** (KBr, cm^−1^): *ν*(NH): 3406, 3321; *ν*(CH): 3184–2971; *ν*(C = N), *ν*(CC), *δ*(CH), *δ*(NH): 1595, 1551, 1506, 1497, 1472, 1437, 1398, 1315, 1275; *ν*(CS), *ν*(C-N), *ν*(NN): 1218, 1199, 1131, 1074. **UV-Vis** (H_2_O/DMSO 50%/50%; λ_max_, nm): = 338.

**Pd(dc-TSC-oCl)_2_** (C_28_H_18_Cl_6_N_6_PdS_2_) (821.75 g/mol), yield (84%). Anal. calculated (%): Pd, 12.95; S, 7.80. Found (%): Pd, 13.07; S, 8.25. **HRMS** (ESI/Q-TOF) *m/z*: calculated for [C_28_H_18_Cl_6_N_6_PdS_2_ + H]^+^: 818.8273. Found: 818.8256. **FTIR** (KBr, cm^−1^): *ν*(NH): 3393, 3266; *ν*(CH): 3129–2958; *ν*(C = N), *ν*(CC), *δ*(CH), *δ*(NH): 1591, 1551, 1472, 1444, 1435, 1397, 1309, 1282; *ν*(CS), *ν*(C-N), *ν*(NN): 1218, 1196, 1129. **UV-Vis** (H_2_O/DMSO 50%/50%; λ_max_, nm): = 338.

**Pd(dc-TSC-mCl)_2_** (C_28_H_18_Cl_6_N_6_PdS_2_) (821.75 g/mol), yield (83%). Anal. calculated (%): Pd, 12.95; S, 7.80. Found (%): Pd, 12.03; S, 8.27. **HRMS** (ESI/Q-TOF) *m/z*: calculated for [C_28_H_18_Cl_6_N_6_PdS_2_ + H]^+^: 818.8273. Found: 818.8273. **FTIR** (KBr, cm^−1^): *ν*(NH): 3391, 3273; *ν*(CH): 3121–2953; *ν*(C = N), *ν*(CC), *δ*(CH), *δ*(NH): 1592, 1539, 1508, 1472, 1435, 1396, 1309, 1275; *ν*(CS), *ν*(C-N), *ν*(NN): 1218, 1189, 1129. **UV-Vis** (H_2_O/DMSO 50%/50%; λ_max_, nm): = 327.

**Pd(dc-TSC-pCl)_2_** (C_28_H_18_Cl_6_N_6_PdS_2_) (821.75 g/mol), yield (79%). Anal. calculated (%): Pd, 12.95; S, 7.80. Found (%): Pd, 13.74; S, 7.52. **HRMS** (ESI/Q-TOF) *m/z*: calculated for [C_28_H_18_Cl_6_N_6_PdS_2_ + H]^+^: 818.8273. Found: 818.8277. **FTIR** (KBr, cm^−1^): *ν*(NH): 3393, 3275; *ν*(CH): 3065, 2952; *ν*(C = N), *ν*(CC), *δ*(CH), *δ*(NH): 1591, 1551, 1540, 1508, 1472, 1434, 1397, 1308; *ν*(CS), *ν*(C-N), *ν*(NN): 1217, 1198, 1129. **UV-Vis** (H_2_O/DMSO 50%/50%; λ_max_, nm): = 336.

#### Complexes based on 4-bromobenzylidene-substituted ligands

**Cu(b-TSC)_2_** (C_28_H_22_Br_2_CuN_6_S_2_) (730.00 g/mol), yield (87%). Anal. calculated (%): Cu, 8.70; S, 8.78. Found (%): Cu, 8.55; S, 8.80. **HRMS** (ESI/Q-TOF) *m/z*: calculated for [C_28_H_22_Br_2_CuN_6_S_2_ + H]^+^: 727.9083. Found: 727.9048. **FTIR** (KBr, cm^−1^): *ν*(NH): 3316; *ν*(CH): 3118–2978; *ν*(C = N), *ν*(CC), *δ*(CH), *δ*(NH): 1591, 1548, 1497, 1485, 1450, 1435, 1400, 1315, 1270; *ν*(CS), *ν*(C-N), *ν*(NN): 1199, 1070. **UV-Vis** (H_2_O/DMSO 50%/50%; λ_max_, nm): = 338.

**Cu(b-TSC-oCl)_2_** (C_28_H_20_Br_2_Cl_2_CuN_6_S_2_) (798.89 g/mol), yield (59%). Anal. calculated (%): Cu, 7.95; S, 8.03. Found (%): Cu, 7.54; S, 8.85. **HRMS** (ESI/Q-TOF) *m/z*: calculated for [C_28_H_20_Br_2_Cl_2_CuN_6_S_2_ + H]^+^: 795.8303. Found: 795.8298. **FTIR** (KBr, cm^−1^): *ν*(NH): 3390, 3265; *ν*(CH): 3096–2948; *ν*(C = N), *ν*(CC), *δ*(CH), *δ*(NH): 1591, 1549, 1514, 1486, 1444, 1432, 1400, 1307, 1275; *ν*(CS), *ν*(C-N), *ν*(NN): 1245, 1200, 1128, 1070. **UV-Vis** (H_2_O/DMSO 50%/50%; λ_max_, nm): = 333.

**Cu(b-TSC-mCl)_2_** (C_28_H_20_Br_2_Cl_2_CuN_6_S_2_) (798.89 g/mol), yield (82%). Anal. calculated (%): Cu, 7.95; S, 8.03. Found (%): Cu, 7.05; S, 8.94. **HRMS** (ESI/Q-TOF) *m/z*: calculated for [C_28_H_20_Br_2_Cl_2_CuN_6_S_2_ + H]^+^: 795.8303. Found: 795.8297. **FTIR** (KBr, cm^−1^): *ν*(NH): 3401, 3313; *ν*(CH): 3114–2975; *ν*(C = N), *ν*(CC), *δ*(CH), *δ*(NH): 1590, 1548, 1507, 1482, 1420, 1400, 1302, 1272; *ν*(CS), *ν*(C-N), *ν*(NN): 1195, 1097, 1070. **UV-Vis** (H_2_O/DMSO 50%/50%; λ_max_, nm): = 339.

**Cu(b-TSC-pCl)_2_** (C_28_H_20_Br_2_Cl_2_CuN_6_S_2_) (798.89 g/mol), yield (78%). Anal. calculated (%): Cu, 7.95; S, 8.03. Found (%): Cu, 7.83; S, 8.34. **HRMS** (ESI/Q-TOF) *m/z*: calculated for [C_28_H_20_Br_2_Cl_2_CuN_6_S_2_ + H]^+^: 795.8303. Found: 795.8289. **FTIR** (KBr, cm^−1^): *ν*(NH): 3403, 3316; *ν*(CH): 3113, 2973; *ν*(C = N), *ν*(CC), *δ*(CH), *δ*(NH): 1589, 1546, 1505, 1488, 1398, 1301, 1270; *ν*(CS), *ν*(C-N), *ν*(NN): 1196, 1090, 1070. **UV-Vis** (H_2_O/DMSO 50%/50%; λ_max_, nm): = 340.

**Pd(b-TSC)_2_** (C_28_H_22_Br_2_N_6_PdS_2_) (772.87 g/mol), yield (70%). Anal. calculated (%): Pd, 13.77; S, 8.30. Found (%): Pd, 13.91; S, 8.12. **HRMS** (ESI/Q-TOF) *m/z*: calculated for [C_28_H_22_Br_2_N_6_PdS_2_ + H]^+^: 770.8822. Found: 770.8833. **FTIR** (KBr, cm^−1^): *ν*(NH): 3313; *ν*(CH): 3057, 2971; *ν*(C = N), *ν*(CC), *δ*(CH), *δ*(NH): 1591, 1558, 1541, 1506, 1484, 1436, 1400, 1315, 1271; *ν*(CS), *ν*(C-N), *ν*(NN): 1194, 1069. **UV-Vis** (H_2_O/DMSO 50%/50%; λ_max_, nm): = 329.

**Pd(b-TSC-oCl)_2_** (C_28_H_20_Br_2_Cl_2_N_6_PdS_2_) (841.76 g/mol), yield (70%). Anal. calculated (%): Pd, 12.64; S, 7.62. Found (%): Pd, 12.99; S, 7.76. **HRMS** (ESI/Q-TOF) *m/z*: calculated for [C_28_H_20_Br_2_Cl_2_N_6_PdS_2_ + H]^+^: 838.8042. Found: 838.8055. **FTIR** (KBr, cm^−1^): *ν*(NH): 3391, 3272; *ν*(CH): 3111–2924; *ν*(C = N), *ν*(CC), *δ*(CH), *δ*(NH): 1590, 1540, 1508, 1485, 1434, 1400, 1307; *ν*(CS), *ν*(C-N), *ν*(NN): 1198, 1129, 1069. **UV-Vis** (H_2_O/DMSO 50%/50%; λ_max_, nm): = 338.

**Pd(b-TSC-mCl)_2_** (C_28_H_20_Br_2_Cl_2_N_6_PdS_2_) (841.76 g/mol), yield (71%). Anal. calculated (%): Pd, 12.64; S, 7.62. Found (%): Pd, 13.23; S, 8.16. **HRMS** (ESI/Q-TOF) *m/z*: calculated for [C_28_H_20_Br_2_Cl_2_N_6_PdS_2_ + H]^+^: 838.8042. Found: 838.8055. **FTIR** (KBr, cm^−1^): *ν*(NH): 3399, 3313; *ν*(CH): 3116–2924; *ν*(C = N), *ν*(CC), *δ*(CH), *δ*(NH): 1590, 1541, 1506, 1482, 1420, 1400, 1309, 1272, 1253; *ν*(CS), *ν*(C-N), *ν*(NN): 1187, 1096, 1070. **UV-Vis** (H_2_O/DMSO 50%/50%; λ_max_, nm): = 337.

**Pd(b-TSC-pCl)_2_** (C_28_H_20_Br_2_Cl_2_N_6_PdS_2_) (841.76 g/mol), yield (86%). Anal. calculated (%): Pd, 12.64; S, 7.62. Found (%): Pd, 13.07; S, 8.00. **HRMS** (ESI/Q-TOF) *m/z*: calculated for [C_28_H_20_Br_2_Cl_2_N_6_PdS_2_ + H]^+^: 838.8042. Found: 838.8044. **FTIR** (KBr, cm^−1^): *ν*(NH): 3397, 3312; *ν*(CH): 3173–2970; *ν*(C = N), *ν*(CC), *δ*(CH), *δ*(NH): 1589, 1558, 1539, 1489, 1398, 1307, 1273; *ν*(CS), *ν*(C-N), *ν*(NN): 1195, 1089, 1069. **UV-Vis** (H_2_O/DMSO 50%/50%; λ_max_, nm): = 337.

### Chemistry

All chemicals and solvents used for synthesis and analysis were purchased from Sigma-Aldrich, Avantor Performance Materials, and Pol-Aura, and used without further purification. The metal and sulphur contents were determined by ICP spectroscopy. Standard solutions from Merck (1000 mg⸱L ^− 1^) were used for the preparation of the diluted solutions used for calibration. For analysis, distilled water obtained with the Polwater system was used. Solid samples were decomposed using the Anton Paar Multiwave 3000 closed-system instrument. Mineralisation was carried out for 45 min at 240 °C under a pressure of 60 bar. The mass spectroscopy (HRMS) analysis was performed with the use of SYNAPT G2 instrument (Waters, USA) under the conditions of electrospray ionisation in positive mode (desolvation temperature 200 °C, source temperature 120 °C, extraction cone voltage 4 V, capillary voltage 3 kV, sampling cone voltage 40 V, desolvation gas 500 L/h). FTIR spectra were recorded with an IRTracer-100 Shimadzu spectrometer (4000–400 cm^−1^) with accuracy of recording equal to 1 cm^−1^, using KBr pellets. UV-Vis spectra were recorded with an UV-1700 PharmaSpec Shimadzu spectrophotometer (260–800 nm) with accuracy of recording equal to 0.5 nm. Stability measurements were performed after 0, 24, and 48 h of stabilisation in a 50:50 (v/v) H_2_O/DMSO or a 90:10 (v/v) DCBM/DMSO medium. The final concentration of each compound was 10 μM. Corresponding 50:50 (v/v) H_2_O/DMSO and 90:10 (v/v) DCBM/DMSO mixtures were used as blanks. Both FTIR and UV-Vis spectra were processed and analysed using Spectragryph software^57^.

### SC-XRD analysis

The single crystal X-ray diffraction (SC-XRD) data were collected on the XtaLAB Synergy Dualflex Pilatus 300K diffractometer. Data collection, cell refinement, data reduction, and absorption correction were carried out using CrysAlisPro software^58^ The structures were solved by intrinsic phasing using SHELXT^59^ and refined by full-matrix least-squares minimisation using SHELXL^60^. All non-hydrogen atoms were refined anisotropically. The carbon-bonded hydrogen atoms were positioned geometrically and refined using the riding model, whereas the nitrogen-bonded hydrogen atoms, involved in the hydrogen bonding, were located from the Fourier difference map and refined freely. Structural visualisations were made using the Mercury 2024.2.0^61^. Geometrical parameters were calculated using the Platon PWT 2021.2^62^. Details concerning crystal data and refinement are given in Table S6. The conventional numbering of the thiosemicarbazone chain used to describe substitution patterns should not be confused with the atom-labelling scheme applied in the crystallographic analysis.

In the structure of **Pd(b-TSC-pCl)_2_**·2DMF, both benzene rings of thiosemicarbazone as well as solvent molecule of DMF exhibit disorder over two positions with ca. 50% participation of domains (0.496:0.504 for benzene containing C1 atom, 0.480:0.520 for benzene containing C9 atom, and 0.515:0.485 for DMF). The solvent mask function was also used in the refinement of **Pd(b-TSC-pCl)_2_**·2DMF, due to the presence of disordered solvent molecules (methanol), which turn out to be impossible to refine. It revealed that there are 16 electrons per unit cell, which is nearly consistent with 1 methanol molecule.

CCDC 2446226–2446239 contain the supplementary crystallographic data for this paper. These data can be obtained free of charge at www.ccdc.cam.ac.uk/conts/retrieving.html [or from the Cambridge Crystallographic Data Centre (CCDC), 12 Union Road, Cambridge CB2 1EZ, UK; fax: +44(0) 1223 336 033; email: deposit@ccdc.cam.ac.uk].

### In silico analysis of physicochemical and ADME-related descriptors

The physicochemical and ADME-related properties of the ligands were predicted using the freely available SwissADME web server developed by the Swiss Institute of Bioinformatics^63^^,^^64^. The two-dimensional structure of each ligand was drawn using the molecular sketcher implemented in SwissADME and converted by the server into the corresponding SMILES representation. The automatically generated reports were used to assess drug-likeness and predict P-glycoprotein substrate status. Bioavailability radar plots and the BOILED-Egg diagram were generated automatically by the server.

### Anticancer activity

#### Cell culturing

Three human malignant melanoma cell lines, A-375 (CRL-1619^™^, American Type Culture Collection, Manassas, VA, USA), SK-MEL-28 (HTB-71^™^, American Type Culture Collection, Manassas, VA, USA), and G-361 (CRL-1424^™^, American Type Culture Collection, Manassas, VA, USA), were used in this study. Additionally, vemurafenib-resistant A-375 and SK-MEL-28 cell lines, established by selecting monoclones using the limiting dilution method after treatment with gradually increasing concentrations of vemurafenib, were included. Normal human skin fibroblast BJ (CRL-2522^™^, American Type Culture Collection, Manassas, VA, USA) cell lines were also used to assess the potential toxicity of the tested compounds. The cell lines were cultured in media optimised for their growth and maintenance:

- A-375 cells were maintained in Dulbecco’s Modified Eagle’s Medium (DMEM) (Corning, Tewksbury, MA, USA).
- G-361 cells were grown in McCoy’s 5A Medium (Corning, Tewksbury, MA, USA).
- SK-MEL-28 and BJ cells were cultured in Eagle’s Minimum Essential Medium (EMEM) (Corning, Tewksbury, MA, USA).

All media were supplemented with 10% foetal bovine serum (FBS), 100 U/mL penicillin, and 100 μg/mL streptomycin (all from Corning, Corning, NY, USA). Cells were incubated at 37 °C under 5% CO_2_ in a humidified incubator. The number of cells to be plated for each experimental approach was determined using a Countess^®^ Automated Cell Counter (Invitrogen, Carlsbad, CA, USA).

#### MTT tests

To evaluate anticancer activity, cells were seeded in 96-well plates at a density of 2 × 10^4^ cells per well and incubated overnight to allow attachment. The cells were then treated with the tested compounds at 10 µM (initial activity screening of all compounds) or at concentrations ranging from 1 to 10 µM (for compounds selected based on the initial screening). The cells were incubated with the tested compounds for 48 h, with DMSO (<0.5% maximum concentration) used as a vehicle in control cultures. The cytotoxic effect was evaluated using the MTT (3–(4,5-dimethylthiazol-2-yl)-2,5-diphenyltetrazolium bromide) assay. MTT solution (4.0 mg/mL) was added to the culture after 48 h of incubation. After 4 h, the medium containing MTT was removed, and the formed crystals were dissolved in DMSO (200 µL/well). The absorbance of the solution was measured at 570 nm using a PowerWave microplate spectrophotometer (BioTek Instruments, Winooski, VT, USA). The experiment was performed in two independent runs, with three replicates for each concentration of the tested compounds. IC_50_ values were determined using the AAT Bioquest IC_50_ calculator^65^.

#### Quantitative real-time PCR analysis (qRT-PCR)

A quantitative real-time polymerase chain reaction (qRT-PCR) was utilised to assess the levels of gene expression. The cells were seeded into 25 cm³ culture flasks at a density of 4 × 10^5^ cells/mL. When the cultures achieved 70–80% confluence, compounds at IC_50_ concentrations were introduced. Following a 48-h incubation period, the cells were detached using a solution of trypsin-EDTA (Corning, Corning, USA) and collected by centrifugation at 400 × g for 5 min at room temperature. The RNA isolation procedure was conducted by the method described by Chomczynski and Sacchi^66^. The lysis of cells was induced through the addition of 1 ml of TRIzol^™^ reagent (Invitrogen, Carlsbad, CA, USA). The lysates were then placed in a centrifuge at 12,000 × g for 5 min at 4 °C. The resulting upper layer was then processed further. Total RNA was dissolved in RNase-free water, and the concentration and purity were determined using a Pico200 Microliter UV/Vis Spectrophotometer. An OD260/280 ratio close to 2.0 was considered acceptable. The synthesis of complementary DNA (cDNA) was conducted using the NG dART RT-PCR kit (EURx, Gdańsk, Poland) and a Mastercycler gradient thermal cycler (Eppendorf, Hamburg, Germany). The reverse transcription process was conducted by the protocol. The temperature was maintained at 25 °C for 10 min, followed by 50 min at 50 °C, and culminating in a final step of enzyme inactivation at 85 °C for 5 min. Quantitative PCR was performed using SG/ROX qPCR Master Mix (2x) reagents (EURx, Gdańsk, Poland) on a 7500 Fast Real-Time PCR System (Applied Biosystems, USA), according to the manufacturer’s instructions. The thermal profile of the reaction consisted of an initial denaturation step at 95 °C for 20 s, followed by 40 cycles of 3 s at 95 °C and 30 s at 60 °C. The analysis employed reference genes, namely RNA18SN5 and ACTB, and the primer sequences are listed in Table S13. Gene expression levels were calculated using the ΔΔCt method, and the results were expressed as relative quantification (RQ = 2^-ΔΔCt).^ Each sample was analysed in triplicate (three technical replicates, Tables S8–S10).

#### Cell cycle analysis

Cell cycle analysis was performed using a NucleoCounter NC-3000 (ChemoMetec, Allerød, Denmark). The procedure was performed according to a two-step protocol provided by the manufacturer. Cells were seeded into 6-well plates at a density of 2 × 10^5^ cells per well, and test compounds were added at concentrations corresponding to their IC_50_ values when the cultures reached approximately 70–80% confluence. In control wells, DMSO was used as a vehicle. Following a 48-h incubation with the compounds, the cells were harvested using a trypsin-EDTA solution (Corning, Corning, USA), washed with PBS (Corning, New York, NY, USA), and resuspended in 250 μL of lysis buffer (Solution 10) containing 10 μg/mL of DAPI (4′,6-diamidino-2-phenylindole dihydrochloride).

The samples were then subjected to an incubation process at a temperature of 37 °C for 5 min. Following the addition of stabilisation buffer (Solution 11), the cell suspensions were loaded into an 8-chamber NC slide and analysed using the NucleoCounter NC-3000 system (ChemoMetec USA Inc., Lillerød, Denmark).

#### Apoptosis detection

The analysis of apoptosis was conducted employing the Annexin V/Propidium Iodide (PI) staining method in conjunction with the NucleoCounter^®^ NC-3000 system (ChemoMetec, Allerød, Denmark). The cells were then seeded into 6-well plates at a density of 2 × 10^5^ cells per well. Once cultures had reached 70–80% confluence, compounds were added at concentrations equivalent to their IC_50_ values. Control samples were treated with dimethyl sulfoxide (DMSO).

Following a 48-h incubation period, the cells were detached using a solution of trypsin-EDTA (Corning, USA). These cells were then washed once with phosphate-buffered saline (PBS) and the cell pellet was obtained by centrifugation at 400 × g for 5 min at room temperature. After the removal of the upper layer, the cell pellet was resuspended in 100 μL of Annexin V binding buffer containing Annexin V–FITC. Next, 2 μL of Annexin V-CF488A conjugate and 4 μL of Hoechst 33342 (10 μg/mL) were added to each sample, followed by gentle mixing. The samples were then incubated for 15 min at 37 °C in the absence of light.

Following the incubation period, the cells were placed into a centrifuge at 400 × g for 5 min. The resultant supernatant was then removed, and the cell pellet was resuspended in 100 μL of binding buffer, which had been supplemented with 10 μg/mL PI. This was prepared by the addition of 2 μL of Solution 16 to 100 μL of buffer. 30 μL of the cell suspension was placed on an NC-slide and analysed using the NC-3000 image cytometer.

#### Activity against vemurafenib-resistant A-375 and SK-MEL-28 cells

Resistance induction was carried out by culturing the cells in the presence of gradually increasing concentrations of vemurafenib (0.5–10 µM). The compound concentration was doubled every two weeks. Every six weeks, the sensitivity of the cell lines to vemurafenib was assessed in comparison to the parental line using the MTT assay after 48 h of incubation with the selected compound over a wide range of concentrations.

#### Anticancer structure–activity relationship descriptors

The descriptors included in the anticancer structure-activity relationship were calculated using Gaussian 09, Revision E0.01^67^. Geometry optimizations and electronic-energy calculations were carried out with the B3LYP hybrid exchange-correlation functional^68^^,^^69^ in conjunction with the def2-SVP basis set^70^. London-dispersion interactions were included using Grimme’s atom-pairwise D3 correction with Becke-Johnson damping, D3(BJ), throughout both geometry optimisation and energy evaluation^71^^,^^72^. All electrons were treated explicitly for elements up to Kr, including Cu; for Pd, the associated 28-electron scalar-relativistic def2 effective-core potential was used^73^. Conventional B3LYP lacks a physically correct asymptotic description of London dispersion. The D3(BJ) correction substantially improves the description of noncovalent interactions and molecular structures, and dispersion-corrected B3LYP has shown good agreement with high-level reference data, including CCSD(T)-based benchmarks, for numerous noncovalent test systems^71^^,^^72^^,^^74^^,^^75^. In the present complexes, dispersion can influence intramolecular contacts, ligand conformations, metal-ligand environments, and relative energies.

Solvent effects were treated using the SMD implicit-solvation model^76^. The geometries of the complexes, anionic ligands, hydrated metal ions, and water molecules were optimised in water. Neutral Cu(II) complexes were treated as doublets, neutral Pd(II) complexes as singlets, and isolated *L*^-^ ligands as singlets. The hydrated ions [Cu(H_2_O)_6_]^2+^ and [Pd(H_2_O)_4_]^2+^ were treated as a doublet and a singlet, respectively.

For each complex, an additional SMD single-point calculation in 1-octanol was performed at the geometry optimised in water, *R*_w_. The vertical electronic transfer energy from water to 1-octanol was calculated as:

$${\Delta E}_{\text{tr}}^{w\to \text{oct}}=E_{\text{oct}}(R_{w})-E_{w}(R_{w})$$

An energy-derived octanol/water partition descriptor was subsequently obtained at *T* = 298.15 K from:

$${\text{log}P}_{E}=-{\Delta E}_{\text{tr}}^{w\to \text{oct}}/(\text{RT ln }10)$$

To quantify the energetic effect of the aqueous environment without introducing structural relaxation, gas-phase single-point calculations were performed at the water-optimised geometries. The vertical electronic solvation energy was defined as:

$$\Delta E_{\text{solv}}^{\text{vert}}=E_{w}\left(R_{w}\right)-E_{\text{gas}}\left(R_{w}\right)$$

A negative value indicates stabilisation of the complex by the aqueous environment. This matched-geometry approach isolates the electronic and continuum-solvent contribution from geometry relaxation and is therefore well suited to comparative analysis within a homologous series. It should be emphasised, however, that the resulting vertical quantity is neither adiabatic solvation energy nor standard Gibbs free energy of solvation.

Solvent-accessible surface areas were calculated from the water-optimised geometries using the Shrake-Rupley algorithm^77^. Bondi van der Waals radii^78^, a water-probe radius of 1.40 Å, and 32,768 surface points per atom were employed. The total solvent-accessible surface area was calculated as:

$$\text{SASA}=\Sigma_{i}\;A_{i}$$

where *A_i_* is the solvent-accessible area assigned to atom *i*. The unweighted three-dimensional polar surface area was defined as:

$$3D-{\text{PSA}}_{\text{NOS}}=\Sigma_{i\in P}A_{i}$$

where the polar atom set *P* comprised N, O, S, and polar H atoms bonded to N, O, or S. Cl and Br atoms were deliberately excluded. The fractional polar surface area was calculated as:

$$f{\text{PSA}}_{\text{NOS}}=3D-{\text{PSA}}_{\text{NOS}}/\text{SASA}.$$

Hirshfeld population analyses were performed at the B3LYP-D3(BJ)/def2-SVP/SMD(water) level using the Pop = Hirshfeld keyword. Charge Model 5 (CM5) charges derived from the Hirshfeld populations were used instead of the unmodified Hirshfeld charges^79^. The charge-weighted polar surface descriptor was defined as:

$${\text{qPSA}}_{\text{NOS}}=\Sigma_{i\in P}\;A_{i}\left|q_{i}^{\text{CM}5}\right|$$

where *q_i_*^CM5^ is the CM5 charge of atom *i*. Absolute charge values were used to prevent cancellation between positively and negatively charged polar atoms. The normalised charge-weighted descriptor was calculated as:

$${\text{qPSA}}_{\text{NOS}}^{\text{norm}}=q{\text{PSA}}_{\text{NOS}}/\text{SASA}.$$

Thus, 3D-PSA(NOS) describes the geometrically accessible polar surface, whereas qPSA(NOS) additionally weights each atomic surface contribution by the magnitude of its local CM5 charge. The normalised descriptor expresses charge-weighted surface polarity relative to the total accessible surface of the complex. SASA and 3D-PSA(NOS) are reported in Å^2^, qPSA(NOS) in e·Å^2^, and qPSA(NOS)/SASA in e. These are custom three-dimensional descriptors and should not be confused with conventional fragment-based topological polar surface area (TPSA) ^80^.

Total dipole moments were extracted directly from the corresponding Gaussian calculations. CM5 charges on the metal centres and, for the open-shell Cu(II) systems, atomic spin densities were obtained from the same population analyses. All electronic-energy differences were converted using 1 Eₕ = 2625.4996 kJ/mol.

LogS was calculated as an average of three independent calculations performed by the earlier mentioned SwissADME platform^63^.

### Antibacterial activity

The antibacterial activity of the tested compounds was evaluated against both Gram-negative bacteria (*Escherichia coli* ATCC 35218) and Gram-positive bacteria (*Listeria monocytogenes* ATCC 15313 and ATCC 35152, *Staphylococcus aureus* ATCC 25923, *Staphylococcus epidermidis* ATCC 12228) using the well-diffusion method. 24 h cultures of microorganisms were centrifuged, and suspensions with a density of 1.5 × 10^8^ CFU/mL (corresponding to a McFarland standard of 0.5) were prepared in a physiological saline solution. The microorganisms were then plated onto Mueller–Hinton Agar (Merck, Darmstadt, Germany). Wells with a diameter of 8 mm were cut from the agar plates and filled with 80 μL of the tested compounds at concentrations ranging from 100 mg/L to 1.563 mg/L, which were prepared as a series of dilutions in DMSO. A negative control consisting of DMSO alone was included. After an 18-h incubation at 37 °C, the inhibition zones around the wells were measured and reported in millimetres. All measurements were performed in triplicate and expressed as mean inhibition zone diameter ± SD. The minimum inhibitory concentrations (MICs) were defined as the lowest tested concentration producing a visible inhibition zone and were expressed in mg/L. Gentamicin (Sigma Aldrich, Saint Louis, MO, USA) was used as the reference antimicrobial agent against Gram-negative bacteria, while Vancomycin (Sigma Aldrich, Saint Louis, MO, USA) was used as the reference antimicrobial agent against Gram-positive bacteria.

## Supplementary Material

Revised_Supplementary_Materials_Clean.docx

## Data Availability

The authors confirm that the data supporting the findings of this study are available within the article and its supplementary materials.

## Generative artificial intelligence (AI) statement

ChatGPT (OpenAI) was used during manuscript preparation to assist with English-language editing, grammar correction, and stylistic refinement. The authors critically reviewed all generated suggestions and remain fully responsible for the accuracy, integrity, and scientific content of the manuscript.
