# Supplementary material for: Structure-activity relationship of Cu(II) and Pd(II) thiosemicarbazone complexes with anticancer and antibacterial properties
Source: J Enzyme Inhib Med Chem. 2026 Oct 15;41(1):2742455. doi: 10.1080/14756366.2026.2742455 (PMC13674658; doi:10.1080/14756366.2026.2742455)
Supplement: Supplementary file 1 [file IENZ_A_2742455_SM7601.docx]

Supplementary Materials

**Structure-activity relationship of Cu(II) and Pd(II) thiosemicarbazone complexes with anticancer and antibacterial properties**

Table S1. Selected bond lengths (Å) of organic ligand molecules in the studied compounds.

| Compound | C1—N1 | N1—C7 | C7—S1 | C7—N2 | N2—N3 | N3—C8 | C8—C9 |
| --- | --- | --- | --- | --- | --- | --- | --- |
| **dm-HTSC*** 1^st^ mol. | 1.418(2) | 1.343(2) | 1.6824(17) | 1.358(2) | 1.3741(19) | 1.289(2) | 1.463(2) |
| **dm-HTSC*** 2^nd^ mol. | 1.423(2) | 1.344(2) | 1.6926(16) | 1.349(2) | 1.3775(19) | 1.282(2) | 1.461(2) |
| **dm-HTSC*** 3^rd^ mol. | 1.422(2) | 1.341(2) | 1.6864(17) | 1.354(2) | 1.3789(19) | 1.279(2) | 1.460(2) |
| **dm-HTSC*** 4^th^ mol. | 1.414(2) | 1.343(2) | 1.6867(17) | 1.354(2) | 1.376(2) | 1.282(2) | 1.458(2) |
| **dm-HTSC-oCl** | 1.4001(18) | 1.3494(18) | 1.6868(14) | 1.3520(18) | 1.3702(16) | 1.2822(18) | 1.4543(19) |
| **dm-HTSC-mCl** | 1.422(5) | 1.349(5) | 1.689(4) | 1.345(5) | 1.380(5) | 1.287(5) | 1.449(6) |
| **dm-HTSC-pCl***  1^st^ mol. | 1.425(2) | 1.343(2) | 1.6877(17) | 1.343(2) | 1.3799(19) | 1.283(2) | 1.461(2) |
| **dm-HTSC-pCl***  2^nd^ mol. | 1.409(2) | 1.354(2) | 1.6823(17) | 1.349(2) | 1.379(2) | 1.283(2) | 1.457(2) |
| **b-HTSC** | 1.420(3) | 1.349(3) | 1.683(2) | 1.352(3) | 1.368(3) | 1.284(3) | 1.458(3) |
| **Cu(dm-TSC)_2_** | 1.4145(19) | 1.354(2) | 1.7485(15) | 1.3077(19) | 1.3885(17) | 1.2959(19) | 1.458(2) |
| **Cu(dm-TSC-oCl)_2_** | 1.411(2) | 1.363(2) | 1.7476(18) | 1.308(2) | 1.389(2) | 1.295(2) | 1.461(2) |
| **Cu(b-TSC)_2_**∙2DMSO | 1.402(2) | 1.363(2) | 1.7416(18) | 1.309(2) | 1.390(2) | 1.293(2) | 1.469(2) |
| **Pd(dm-TSC)_2_** | 1.4201(16) | 1.3573(17) | 1.7491(12) | 1.3074(16) | 1.3905(15) | 1.3001(17) | 1.4564(17) |
| **Pd(dm-TSC-oCl)_2_** | 1.413(3) | 1.361(3) | 1.750(2) | 1.305(3) | 1.392(3) | 1.287(3) | 1.458(3) |
| **Pd(dm-TSC-pCl)_2_** | 1.4233(17) | 1.3536(18) | 1.7468(13) | 1.3126(18) | 1.3914(16) | 1.3035(18) | 1.4558(17) |
| **Pd(dc-TSC)_2_**∙2DMF | 1.4082(19) | 1.3143(19) | 1.7487(15) | 1.3143(19 | 1.3930(16) | 1.3005(19) | 1.464(2) |
| **Pd(b-TSC)_2_**∙2DMF | 1.405(4) | 1.372(4) | 1.744(3) | 1.303(4) | 1.390(3) | 1.302(4) | 1.452(4) |
| **Pd(b-TSC-pCl)_2_**∙2DMF | 1.410(3) | 1.360(3) | 1.751(3) | 1.307(3) | 1.383(3) | 1.297(3) | 1.464(3) |

* Compound containing more than one symmetrically independent organic ligand molecules in the asymmetric unit. The atom numbering in the second, third and fourth molecules corresponds to that of the first molecule as follows: N1→N21→N41→N61, C1→C21→C41→C61, etc.

Table S2. Selected torsion angles (τ) and dihedral angles between mean planes of benzene rings (φ) of thiosemicarbazone molecules in the studied compounds.

| Compound | τ (°) | | | | φ (°) |
| --- | --- | --- | --- | --- | --- |
|  | C1—N1—C7—N2 | N1—C7—N2—N3 | C7—N2—N3—C8 | N2—N3—C8—C9 |  |
| **dm-HTSC*** 1^st^ mol. | -172.72(17) | 2.1(2) | 178.86(16) | -179.14(15) | 14.1 |
| **dm-HTSC*** 2^nd^ mol. | 169.78(16) | -4.2(2) | -175.05(16) | 179.12(15) | 62.5 |
| **dm-HTSC*** 3^rd^ mol. | 174.35(16) | -4.7(2) | -176.36(16) | 178.61(15) | 66.0 |
| **dm-HTSC*** 4^th^ mol. | -174.85(18) | 3.5(2) | -179.93(17) | -178.51(16) | 11.9 |
| **dm-HTSC-oCl** | -175.83(14) | 0.33(18) | 177.54(13) | 179.06(12) | 6.4 |
| **dm-HTSC-mCl** | 173.9(4) | -9.7(5) | 178.6(4) | 174.6(4) | 35.7 |
| **dm-HTSC-pCl***  1^st^ mol. | -179.57(17) | -0.5(2) | 172.30(16) | 177.44(15) | 76.8 |
| **dm-HTSC-pCl***  2^nd^ mol. | 170.05(16) | -2.0(2) | -175.01(16) | -178.80(15) | 45.5 |
| **b-HTSC** | -176.3(2) | 2.7(3) | 176.46(19) | -176.93(18) | 58.8 |
| **Cu(dm-TSC)_2_** | 11.5(2) | 178.82(13) | -163.83(13) | 3.1(2) | 61.7 |
| **Cu(dm-TSC-oCl)_2_** | 10.7(3) | -179.91(16) | -162.06(17) | 1.5(3) | 62.9 |
| **Cu(b-TSC)_2_**∙2DMSO | 2.1(3) | 179.39(16) | -174.05(17) | 3.1(3) | 23.8 |
| **Pd(dm-TSC)_2_** | -13.1(2) | -177.03(11) | 163.70(12) | -3.1(2) | 63.6 |
| **Pd(dm-TSC-oCl)_2_** | -13.2(3) | -177.95(19) | 162.5(2) | -1.5(4) | 61.3 |
| **Pd(dm-TSC-pCl)_2_** | -11.3(2) | 179.73(12) | 167.64(13) | -2.2(2) | 67.9 |
| **Pd(dc-TSC)_2_**∙2DMF | -3.4(3) | -179.35(13) | -173.21(14) | 1.8(3) | 6.4 |
| **Pd(b-TSC)_2_**∙2DMF | -2.3(4) | -175.8(2) | 171.8(2) | 2.1(4) | 46.6 |
| **Pd(b-TSC-pCl)_2_**∙2DMF | -5.3(4) | 180.0(2) | 170.9(2) | 4.6(4) | ** |

* Compound containing more than one symmetrically independent organic ligand molecules in the asymmetric unit. The atom numbering in the second, third and fourth molecules corresponds to that of the first molecule as follows: N1→N21→N41→N61, C1→C21→C41→C61, etc. ** both benzene rings are disordered over two position.

Table S3. Hydrogen bonds in studied compounds [G_d_^a^(n) – unitary graph set].

| D-H•••A | d(D—H) (Å) | d(H•••A) (Å) | d(D•••A) (Å) | <(DHA) (°) | G_d_^a^(n) |
| --- | --- | --- | --- | --- | --- |
| **dm-HTSC** | | | | | |
| N1—H1•••N3 | 0.87(2) | 2.12(3) | 2.597(2) | 115(2) | S(5) |
| N2—H2•••S21[i] | 0.79(3) | 2.67(3) | 3.4503(18) | 170(2) | D(2) |
| N21—H21•••N23 | 0.78(2) | 2.22(3) | 2.632(2) | 113(2) | S(5) |
| N22—H22•••S1[i] | 0.85(3) | 2.51(3) | 3.3470(17) | 167(2) | D(2) |
| N41—H41•••N43 | 0.82(3) | 2.18(3) | 2.608(2) | 113(2) | S(5) |
| N42—H42•••S41[ii] | 0.78(3) | 2.65(3) | 3.4153(19) | 169(2) | R_2_^2^(8) |
| N61—H61•••N63 | 0.84(3) | 2.14(3) | 2.604(2) | 114(2) | S(5) |
| N62—H62•••S61[iii] | 0.79(3) | 2.64(3) | 3.410(2) | 167(2) | R_2_^2^(8) |
| **dm-HTSC-oCl** | | | | | |
| N1—H1•••N3 | 0.822(19) | 2.057(19) | 2.5555(17) | 118.7(16) | S(5) |
| N1—H1•••Cl1 | 0.822(19) | 2.418(18) | 2.9058(13) | 118.9(15) | S(5) |
| N2—H2•••S1[iv] | 0.86(2) | 2.515(19) | 3.3566(13) | 165.4(16) | R_2_^2^(8) |
| **dm-HTSC-mCl** | | | | | |
| N1—H1•••N3 | 0.88(6) | 2.15(5) | 2.590(5) | 110(4) | S(5) |
| N1—H1•••Cl1[v] | 0.88(6) | 3.00(5) | 3.614(5) | 128(4) | C(6) |
| N2—H2•••S1[iii] | 0.93(5) | 2.45(5) | 3.276(4) | 148(3) | R_2_^2^(8) |
| **dm-HTSC-pCl** | | | | | |
| N1—H1•••N3 | 0.80(3) | 2.22(2) | 2.607(2) | 110(2) | S(5) |
| N2—H2•••S21[vi] | 0.81(2) | 2.55(2) | 3.3424(16) | 167(2) | D(2) |
| N21—H21•••N23 | 0.86(2) | 2.13(2) | 2.595(2) | 113.3(19) | S(5) |
| N22—H22•••S1[vii] | 0.77(2) | 2.58(2) | 3.3278(16) | 162(2) | D(2) |
| **b-HTSC** | | | | | |
| N1—H1•••N3 | 0.84(3) | 2.17(3) | 2.599(3) | 112(3) | S(5) |
| N1—H1•••Br1[viii] | 0.84(3) | 2.94(3) | 3.526(3) | 128(3) | C(11) |
| N2—H2•••S1[ix] | 0.82(3) | 2.59(3) | 3.379(2) | 163(3) | R_2_^2^(8) |
| **Cu(dm-TSC)_2_** | | | | | |
| N1—H1•••O1[x] | 0.78(2) | 2.19(2) | 2.9646(17) | 177(2) | C(10) |
| N1—H1•••O2[x] | 0.78(2) | 2.596(19) | 3.0452(18) | 118.6(17) | C(11) |
| **Cu(dm-TSC-oCl)_2_** | | | | | |
| N1—H1•••O1[xi] | 0.79(2) | 2.23(2) | 3.022(2) | 176(3) | C(10) |
| N1—H1•••O2[xi] | 0.79(2) | 2.67(2) | 3.13 | 120(3) | C(11) |
| **Cu(b-TSC)_2_**∙2DMSO | | | | | |
| N1—H1•••O21 | 0.78(2) | 2.17(2) | 2.950(2) | 172(2) | D(2) |
| **Pd(dm-TSC)_2_** | | | | | |
| N1—H1•••O1[xii] | 0.824(18) | 2.128(18) | 2.9494(15) | 174.9(19) | C(10) |
| N1—H1•••O2[xii] | 0.824(18) | 2.559(19) | 3.0378(16) | 118.3(15) | C(11) |
| **Pd(dm-TSC-oCl)_2_** | | | | | |
| N1—H1•••O1[xiii] | 0.81(3) | 2.21(3) | 3.017(3) | 174(3) | C(10) |
| N1—H1•••O2[xiii] | 0.81(3) | 2.68(3) | 3.122(3) | 116(3) | C(11) |
| **Pd(dm-TSC-pCl)_2_** | | | | | |
| N1—H1•••O1[xiv] | 0.82(2) | 2.20(2) | 3.0050(16) | 172.9(18) | C(10) |
| N1—H1•••O2[xiv] | 0.82(2) | 2.588(19) | 3.0942(17) | 121.7(16) | C(11) |
| **Pd(dc-TSC)_2_**∙2DMF | | | | | |
| N1—H1•••O21 | 0.82(2) | 2.01(2) | 2.8276(19) | 175.7(18) | D(2) |
| **Pd(b-TSC)_2_**∙2DMF | | | | | |
| N1—H1•••O21 | 0.82(3) | 2.15(3) | 2.950(4) | 166(3) | D(2) |
| **Pd(b-TSC-pCl)_2_**∙2DMF | | | | | |
| N1—H1•••O21 | 0.83(3) | 1.92(3) | 2.723(9) | 166(3) | D(2) |
| N1—H1•••O21B | 0.83(3) | 2.05(3) | 2.861(9) | 166(3) | D(2) |

Symmetry transformations used to generate equivalent atoms: [i] 2-x,-y,1-z; [ii] 1-x,1-y,2-z; [iii] 1-x,1-y,1-z; [iv] -x,2-y,1-z; [v] x,-1+y,z; [vi] x,y,-1+z [vii] x,y,1+z; [viii] 1/2-x,1/2+y,1/2-z; [ix] 1-x,2-y,1-z; [x] x,1/2-y,-1/2+z; [xi] -1/2+x,1.5-y,-1/2+z; [xii] x,1.5-y,-1/2+z; [xiii] -1/2+x,1/2-y,-1/2+z; [xiv] x,1.5-y,1/2+z.

Figure S1. Hydrogen bond synthon R_2_^2^(8) in the crystal structure of **dm-HTSC-oCl**.

Table S4. Selected structural data (d – bond length, α – angle) of coordination polyhedra in the studied coordination compounds.

| Compound | d(M1—S1) (Å) | d(M1—N3) (Å) | α(S1—M1—N3) (°) | α(S1—M1—N3[i]) (°) |
| --- | --- | --- | --- | --- |
| **Cu(dm-TSC)_2_** | 2.2410(4) | 2.0217(12) | 84.32(4) | 95.68(4) |
| **Cu(dm-TSC-oCl)_2_** | 2.2565(5) | 2.0117(15) | 83.94(4) | 96.06(4) |
| **Cu(b-TSC)_2_**∙2DMSO | 2.2428(4) | 2.0306(14) | 83.60(4) | 96.40(4) |
| **Pd(dm-TSC)_2_** | 2.2880(3) | 2.0341(10) | 82.99(3) | 97.01(3) |
| **Pd(dm-TSC-oCl)_2_** | 2.2976(6) | 2.0385(18) | 82.64(5) | 97.36(5) |
| **Pd(dm-TSC-pCl)_2_** | 2.2882(4) | 2.0386(11) | 83.02(4) | 96.98(4) |
| **Pd(dc-TSC)_2_**∙2DMF | 2.2832(4) | 2.0418(12) | 82.59(4) | 97.41(4) |
| **Pd(b-TSC)_2_**∙2DMF | 2.2920(7) | 2.040(2) | 82.97(7) | 97.03(7) |
| **Pd(b-TSC-pCl)_2_**∙2DMF | 2.2991(7) | 2.031(2) | 82.87(6) | 97.13(7) |

Abbreviation: M – respective metal cation (Cu or Pd). [i] - symmetry transformations used to generate equivalent atoms: -x, 1-y, -z for **Cu(dm-TSC)_2_**, **Pd(dm-TSC)_2_**, ; 1-x, 1-y, -z for **Cu(dm-TSC-oCl)_2_**, **Pd(TSC-oCl)_2_**; 1-x, 1-y, 1-z for **Cu(b-TSC)_2_**∙2DMSO, **Pd(b-TSC)_2_**∙2DMF, **Pd(b-TSC-pCl)_2_**∙2DMF 1-x, 1-y, 2-z for **Pd(dm-TSC-pCl)_2_**; 2-x, -y, 1-z for **Pd(dc-TSC)_2_**∙2DMF.

Table S5. Continuous Shape Measures values S_Q_(P), where Q is a considered polyhedron and P is an ideal polyhedron, for the coordination polyhedra in the studied coordination compounds.

| Compound | Q | S_Q_(*SP*-4) | S_Q_(*T*-4) | S_Q_(*SS*-4) |
| --- | --- | --- | --- | --- |
| **Cu(dm-TSC)_2_** | Cu1 | 0.508 | 33.672 | 18.023 |
| **Cu(dm-TSC-oCl)_2_** | Cu1 | 0.605 | 33.737 | 17.938 |
| **Cu(b-TSC)_2_**∙2DMSO | Cu1 | 0.556 | 33.704 | 18.110 |
| **Pd(dm-TSC)_2_** | Pd1 | 0.715 | 33.810 | 17.985 |
| **Pd(dm-TSC-oCl)_2_** | Pd1 | 0.765 | 33.843 | 17.997 |
| **Pd(dm-TSC-pCl)_2_** | Pd1 | 0.700 | 33.800 | 18.002 |
| **Pd(dc-TSC)_2_**∙2DMF | Pd1 | 0.725 | 33.817 | 18.074 |
| **Pd(b-TSC)_2_**∙2DMF | Pd1 | 0.711 | 33.806 | 17.996 |
| **Pd(b-TSC-pCl)_2_**∙2DMF | Pd1 | 0.765 | 33.843 | 17.939 |

Table S6. Crystal data and structure refinement details for studied compounds.

| Compound | **dm-HTSC** | **dm-HTSC-oCl** | **dm-HTSC-mCl** | **dm-HTSC-pCl** |
| --- | --- | --- | --- | --- |
| CCDC number | 2446226 | 2446230 | 2446228 | 2446229 |
| Empirical formula | C_16_H_17_N_3_O_2_S | C_16_H_16_N_3_O_2_SCl | C_16_H_16_N_3_O_2_SCl | C_16_H_16_ClN_3_O_2_S |
| Formula weight | 315.38 | 349.83 | 349.83 | 349.83 |
| Temperature (K) | 99.9(5) | 99.9(4) | 100.00(10) | 99.9(6) |
| Crystal system | triclinic | monoclinic | monoclinic | triclinic |
| Space group | *P*-1 | *P*2_1_/*c* | *P*2_1_/*n* | *P*-1 |
| *a* (Å) | 12.41600(15) | 17.0722(3) | 15.4307(9) | 9.35070(10) |
| *b* (Å) | 16.31854(20) | 4.90310(10) | 5.3267(3) | 11.3242(2) |
| *c* (Å) | 16.71163(15) | 20.7177(3) | 21.3570(14) | 15.2803(2) |
| *α* (°) | 105.2224(9) | 90 | 90 | 94.4000(10) |
| *β* (°) | 108.8256(9) | 110.231(2) | 109.295(7) | 92.1530(10) |
| *γ* (°) | 90.9400(10) | 90 | 90 | 90.4480(10) |
| Volume (Å^3^) | 3073.37(6) | 1627.22(5) | 1656.83(19) | 1612.02(4) |
| Z | 8 | 4 | 4 | 4 |
| ρ_calc_ (g/cm^3^) | 1.363 | 1.428 | 1.402 | 1.441 |
| μ (mm^‑1^) | 1.964 | 3.389 | 3.328 | 3.421 |
| *F(000)* | 1328.0 | 728.0 | 728.0 | 728.0 |
| Crystal size (*mm^3^)* | 0.19 × 0.136 × 0.034 | 0.351 × 0.038 × 0.022 | 0.19 × 0.05 × 0.02 | 0.236 × 0.039 × 0.031 |
| Radiation | Cu Kα (λ = 1.54184) | Cu Kα (λ = 1.54184) | Cu Kα (λ = 1.54184) | Cu Kα (λ = 1.54184) |
| 2*θ* range for data collect. (°) | 6.872 to 159.438 | 8.86 to 157.416 | 6.202 to 158.864 | 7.83 to 159.262 |
| Index ranges | -15 ≤ h ≤ 15,  -20 ≤ k ≤ 20,  -20 ≤ l ≤ 21 | -21 ≤ h ≤ 20,  -6 ≤ k ≤ 5,  -22 ≤ l ≤ 25 | -14 ≤ h ≤ 19,  -6 ≤ k ≤ 6,  -27 ≤ l ≤ 26 | -11 ≤ h ≤ 11,  -14 ≤ k ≤ 14,  -19 ≤ l ≤ 19 |
| Reflections collected | 24415 | 16863 | 15726 | 7940 |
| Independent reflections | 24415 [R_int_ = 0.0661] | 3225 [R_int_ = 0.0389] | 3425 [R_int_ = 0.1077] | 7940 [R_int_ = 0.0255] |
| Data/restraints/parameters | 24415/0/826 | 3225/0/216 | 3425/0/216 | 7940/0/432 |
| Goodness-of-fit on *F*^2^ | 1.073 | 1.052 | 1.029 | 1.137 |
| Final *R* indexes [*I*>2s(*I*)] | R_1_ = 0.0473,  wR_2_ = 0.1388 | R_1_ = 0.0278,  wR_2_ = 0.0712 | R_1_ = 0.0672,  wR_2_ = 0.1690 | R_1_ = 0.0463,  wR_2_ = 0.1084 |
| R indexes (all data) | R_1_ = 0.0547,  wR_2_ = 0.1444 | R_1_ = 0.0305,  wR_2_ = 0.0728 | R_1_ = 0.1146,  wR_2_ = 0.2019 | R_1_ = 0.0494,  wR_2_ = 0.1104 |
| Largest diff. peak and hole (e•Å^-3^) | 0.91/-0.36 | 0.27/-0.24 | 0.62/-0.50 | 0.98/-0.89 |

Table S6. *Continued*.

| Compound | **b-HTSC** | **Cu(dm-TSC)_2_** | **Cu(dm-TSC-oCl)_2_** | **Cu(b-TSC)_2_∙**2DMSO |
| --- | --- | --- | --- | --- |
| CCDC number | 2446227 | 2446231 | 2446232 | 2446233 |
| Empirical formula | C_14_H_12_N_3_SBr | C_32_H_32_N_6_O_4_S_2_Cu | C_32_H_30_N_6_O_4_S_2_Cl_2_Cu | C_32_H_34_N_6_O_2_S_4_CuBr_2_ |
| Formula weight | 334.24 | 692.29 | 761.18 | 886.25 |
| Temperature (K) | 100.00(10) | 100.00(10) | 100.00(10) | 100.00(10) |
| Crystal system | monoclinic | monoclinic | monoclinic | orthorhombic |
| Space group | *P*2_1_/*n* | *P*2_1_/*c* | *P*2_1_/*n* | *Pbcn* |
| *a* (Å) | 14.8848(3) | 11.09320(10) | 11.51830(10) | 16.3382(2) |
| *b* (Å) | 6.70130(10) | 9.26920(10) | 9.57390(10) | 27.0246(3) |
| *c* (Å) | 15.0459(3) | 16.5271(2) | 15.9017(2) | 8.02530(10) |
| *α* (°) | 90 | 90 | 90 | 90 |
| *β* (°) | 111.775(2) | 108.2550(10) | 107.6720(10) | 90 |
| *γ* (°) | 90 | 90 | 90 | 90 |
| Volume (Å^3^) | 1393.71(5) | 1613.87(3) | 1670.81(3) | 3543.44(7) |
| Z | 4 | 2 | 2 | 4 |
| ρ_calc_ (g/cm^3^) | 1.593 | 1.425 | 1.513 | 1.661 |
| μ (mm^‑1^) | 5.326 | 2.548 | 3.955 | 6.039 |
| *F(000)* | 672.0 | 718.0 | 782.0 | 1788.0 |
| Crystal size (*mm^3^)* | 0.20 × 0.04 × 0.02 | 0.09 × 0.08 × 0.02 | 0.22 × 0.12 × 0.06 | 0.53 × 0.03 × 0.01 |
| Radiation | Cu Kα (λ = 1.54184) | Cu Kα (λ = 1.54184) | Cu Kα (λ = 1.54184) | Cu Kα (λ = 1.54184) |
| 2*θ* range for data collect. (°) | 7.134 to 158.632 | 8.394 to 159.19 | 8.392 to 158.522 | 6.322 to 158.734 |
| Index ranges | -18 ≤ h ≤ 15,  -8 ≤ k ≤ 8,  -19 ≤ l ≤ 18 | -13 ≤ h ≤ 13,  -11 ≤ k ≤ 10,  -20 ≤ l ≤ 21 | -14 ≤ h ≤ 11,  -12 ≤ k ≤ 12,  -19 ≤ l ≤ 19 | -20 ≤ h ≤ 19,  -29 ≤ k ≤ 34,  -10 ≤ l ≤ 7 |
| Reflections collected | 14324 | 26718 | 23813 | 26466 |
| Independent reflections | 2871 [R_int_ = 0.0370] | 3340 [R_int_ = 0.0340] | 3390 [R_int_ = 0.0319] | 3700 [R_int_ = 0.0315] |
| Data/restraints/parameters | 2871/0/178 | 3340/0/210 | 3390/0/219 | 3700/0/219 |
| Goodness-of-fit on *F*^2^ | 1.101 | 1.062 | 1.189 | 1.058 |
| Final *R* indexes [*I*>2s(*I*)] | R_1_ = 0.0269,  wR_2_ = 0.0683 | R_1_ = 0.0254,  wR_2_ = 0.0597 | R_1_ = 0.0273,  wR_2_ = 0.0714 | R_1_ = 0.0253,  wR_2_ = 0.0605 |
| R indexes (all data) | R_1_ = 0.0305,  wR_2_ = 0.0794 | R_1_ = 0.0288,  wR_2_ = 0.0657 | R_1_ = 0.0301,  wR_2_ = 0.0798 | R_1_ = 0.0301,  wR_2_ = 0.0641 |
| Largest diff. peak and hole (e•Å^-3^) | 0.56/-0.53 | 0.32/-0.38 | 0.40/-0.35 | 0.31/-0.40 |

Table S6. *Continued*.

| Compound | **Pd(dm-TSC)_2_** | **Pd(dm-TSC-oCl)_2_** | **Pd(dm-TSC-pCl)_2_** | **Pd(dc-TSC)_2_**∙2DMF |
| --- | --- | --- | --- | --- |
| CCDC number | 2446234 | 2446235 | 2446236 | 2446237 |
| Empirical formula | C_32_H_32_N_6_O_4_S_2_Pd | C_32_H_30_N_6_O_4_S_2_Cl_2_Pd | C_32_H_30_N_6_O_4_S_2_Cl_2_Pd | C_34_H_34_N_8_O_2_S_2_Cl_4_Pd |
| Formula weight | 735.15 | 804.04 | 804.04 | 899.01 |
| Temperature (K) | 100.00(10) | 110.00(10) | 100.00(10) | 100.00(10) |
| Crystal system | monoclinic | monoclinic | monoclinic | triclinic |
| Space group | *P*2_1_/*c* | *P*2_1_/*n* | *P*2_1_/*c* | *P*-1 |
| *a* (Å) | 11.0710(3) | 11.5195(2) | 10.8150(3) | 7.8961(2) |
| *b* (Å) | 9.3454(2) | 9.63960(10) | 9.4643(2) | 9.5257(2) |
| *c* (Å) | 16.4370(4) | 15.9220(2) | 16.5802(4) | 13.2760(3) |
| *α* (°) | 90 | 90 | 90 | 69.407(2) |
| *β* (°) | 107.582(2) | 108.2580(10) | 102.428(2) | 75.989(2) |
| *γ* (°) | 90 | 90 | 90 | 80.568(2) |
| Volume (Å^3^) | 1621.18(7) | 1679.02(4) | 1657.32(7) | 903.54(4) |
| Z | 2 | 2 | 2 | 1 |
| ρ_calc_ (g/cm^3^) | 1.506 | 1.590 | 1.611 | 1.652 |
| μ (mm^‑1^) | 0.748 | 7.477 | 0.895 | 0.971 |
| *F(000)* | 752.0 | 816.0 | 816.0 | 456.0 |
| Crystal size (*mm^3^)* | 0.21 × 0.11 × 0.04 | 0.09 × 0.05 × 0.02 | 0.26 × 0.17 × 0.11 | 0.2 × 0.12 × 0.05 |
| Radiation | Mo Kα (λ = 0.71073) | Cu Kα (λ = 1.54184) | Mo Kα (λ = 0.71073) | Mo Kα (λ = 0.71073) |
| 2*θ* range for data collect. (°) | 5.076 to 63.142 | 8.362 to 157.364 | 4.986 to 62.35 | 5.338 to 63.322 |
| Index ranges | -15 ≤ h ≤ 15,  -13 ≤ k ≤ 13,  -22 ≤ l ≤ 22 | -12 ≤ h ≤ 14,  -11 ≤ k ≤ 11,  -19 ≤ l ≤ 19 | -14 ≤ h ≤ 15,  -12 ≤ k ≤ 13,  -23 ≤ l ≤ 23 | -11 ≤ h ≤ 10,  -13 ≤ k ≤ 12,  -19 ≤ l ≤ 18 |
| Reflections collected | 50154 | 17092 | 22336 | 23930 |
| Independent reflections | 4902 [R_int_ = 0.0415] | 3407 [R_int_ = 0.0305] | 4798 [R_int_ = 0.0359] | 5148 [R_int_ = 0.0379] |
| Data/restraints/parameters | 4902/0/210 | 3407/0/219 | 4798/0/219 | 5148/0/237 |
| Goodness-of-fit on *F*^2^ | 1.046 | 1.046 | 1.082 | 1.036 |
| Final *R* indexes [*I*>2s(*I*)] | R_1_ = 0.0229,  wR_2_ = 0.0541 | R_1_ = 0.0273,  wR_2_ = 0.0694 | R_1_ = 0.0241,  wR_2_ = 0.0593 | R_1_ = 0.0256,  wR_2_ = 0.0575 |
| R indexes (all data) | R_1_ = 0.0297,  wR_2_ = 0.0569 | R_1_ = 0.0306,  wR_2_ = 0.0710 | R_1_ = 0.0283,  wR_2_ = 0.0611 | R_1_ = 0.0310,  wR_2_ = 0.0594 |
| Largest diff. peak and hole (e•Å^-3^) | 0.59/-0.66 | 0.98/-0.56 | 0.47/-0.72 | 0.44/-0.54 |

Table S6. *Continued*.

| Compound | **Pd(b-TSC)_2_**∙2DMF | **Pd(b-TSC-pCl)_2_**∙2DMF |
| --- | --- | --- |
| CCDC number | 2446238 | 2446239 |
| Empirical formula | C_34_H_36_N_8_O_2_S_2_Br_2_Pd | C_35_H_38_Br_2_Cl_2_N_8_O_3_PdS_2_ |
| Formula weight | 919.05 | 1019.97 |
| Temperature (K) | 100.00(10) | 100.00(10) |
| Crystal system | monoclinic | triclinic |
| Space group | *C*2/*c* | *P*-1 |
| *a* (Å) | 26.5119(4) | 5.1134(2) |
| *b* (Å) | 15.8068(2) | 11.1113(4) |
| *c* (Å) | 8.49430(10) | 18.1884(7) |
| *α* (°) | 90 | 84.191(3) |
| *β* (°) | 93.5350(10) | 89.363(3) |
| *γ* (°) | 90 | 78.018(3) |
| Volume (Å^3^) | 3552.92(8) | 1005.64(7) |
| Z | 4 | 1 |
| ρ_calc_ (g/cm^3^) | 1.718 | 1.684 |
| μ (mm^‑1^) | 8.310 | 2.731 |
| *F(000)* | 1840.0 | 510.0 |
| Crystal size (*mm^3^)* | 0.2 × 0.02 × 0.02 | 0.191 × 0.034 × 0.018 |
| Radiation | Cu Kα (λ = 1.54184) | Mo Kα (λ = 0.71073) |
| 2*θ* range for data collect. (°) | 6.514 to 159.428 | 5.572 to 62.414 |
| Index ranges | -33 ≤ h ≤ 26,  -20 ≤ k ≤ 19,  -9 ≤ l ≤ 10 | -7 ≤ h ≤ 7,  -15 ≤ k ≤ 16,  -26 ≤ l ≤ 25 |
| Reflections collected | 18109 | 26255 |
| Independent reflections | 3754 [R_int_ = 0.0354] | 5761 [R_int_ = 0.0604] |
| Data/restraints/parameters | 3754/0/228 | 5761/0/329 |
| Goodness-of-fit on *F*^2^ | 1.149 | 1.054 |
| Final *R* indexes [*I*>2s(*I*)] | R_1_ = 0.0256,  wR_2_ = 0.0585 | R_1_ = 0.0382,  wR_2_ = 0.0782 |
| R indexes (all data) | R_1_ = 0.0301,  wR_2_ = 0.0699 | R_1_ = 0.0556,  wR_2_ = 0.0822 |
| Largest diff. peak and hole (e•Å^-3^) | 0.50/-0.51 | 1.27/-1.22 |

Figure S2. Hydrogen bond synthons C(10) and C(11) in the crystal structure of **Cu(dm-TSC)_2_**.

Figure S3. ESI-MS spectrum of **Cu(dm-TSC)_2_** (left) and an enlarged view of the experimental isotope pattern of the detected [M + H]⁺ ion (right). Peaks marked with an asterisk (*) are assigned to leucine enkephalin and its fragments. Leucine enkephalin was used as a lock-mass reference for internal mass calibration. The peak marked with a number sign (#) is assigned to a fragment derived from the thiosemicarbazone core: [C_5_H_2_N_3_S + H]^+^ (calcd. m/z = 137.0042).

Figure S4. ESI-MS spectrum of **Cu(dm-TSC-oCl)_2_** (left) and an enlarged view of the experimental isotope pattern of the detected [M + H]⁺ ion (right). Peaks marked with an asterisk (*) are assigned to leucine enkephalin and its fragments. Leucine enkephalin was used as a lock-mass reference for internal mass calibration. The peak marked with a number sign (#) is assigned to a fragment derived from the thiosemicarbazone core: [C_5_H_2_N_3_S + H]^+^ (calcd. m/z = 137.0042).

Figure S5. ESI-MS spectrum of **Cu(dm-TSC-mCl)_2_** (left) and an enlarged view of the experimental isotope pattern of the detected [M + H]⁺ ion (right). Peaks marked with an asterisk (*) are assigned to leucine enkephalin and its fragments. Leucine enkephalin was used as a lock-mass reference for internal mass calibration. The peak marked with a number sign (#) is assigned to a fragment derived from the thiosemicarbazone core: [C_5_H_2_N_3_S + H]^+^ (calcd. m/z = 137.0042).

Figure S6. ESI-MS spectrum of **Cu(dm-TSC-pCl)_2_** (left) and an enlarged view of the experimental isotope pattern of the detected [M + H]⁺ ion (right). Peaks marked with an asterisk (*) are assigned to leucine enkephalin and its fragments. Leucine enkephalin was used as a lock-mass reference for internal mass calibration.

Figure S7. ESI-MS spectrum of **Pd(dm-TSC)_2_** (left) and an enlarged view of the experimental isotope pattern of the detected [M + H]⁺ ion (right). Peaks marked with an asterisk (*) are assigned to leucine enkephalin and its fragments. Leucine enkephalin was used as a lock-mass reference for internal mass calibration. The peak marked with a number sign (#) is assigned to a fragment derived from the thiosemicarbazone core: [C_5_H_2_N_3_S + H]^+^ (calcd. m/z = 137.0042).

 Figure S8. ESI-MS spectrum of **Pd(dm-TSC-oCl)_2_** (left) and an enlarged view of the experimental isotope pattern of the detected [M + H]⁺ ion (right). Peaks marked with an asterisk (*) are assigned to leucine enkephalin and its fragments. Leucine enkephalin was used as a lock-mass reference for internal mass calibration. The peak marked with a number sign (#) is assigned to a fragment derived from the thiosemicarbazone core: [C_5_H_2_N_3_S + H]^+^ (calcd. m/z = 137.0042).

 Figure S9. ESI-MS spectrum of **Pd(dm-TSC-mCl)_2_** (left) and an enlarged view of the experimental isotope pattern of the detected [M + H]⁺ ion (right). Peaks marked with an asterisk (*) are assigned to leucine enkephalin and its fragments. Leucine enkephalin was used as a lock-mass reference for internal mass calibration. The peak marked with a number sign (#) is assigned to a fragment derived from the thiosemicarbazone core: [C_5_H_2_N_3_S + H]^+^ (calcd. m/z = 137.0042).

 Figure S10. ESI-MS spectrum of **Pd(dm-TSC-pCl)_2_** (left) and an enlarged view of the experimental isotope pattern of the detected [M + H]⁺ ion (right). Peaks marked with an asterisk (*) are assigned to leucine enkephalin and its fragments. Leucine enkephalin was used as a lock-mass reference for internal mass calibration. The peak marked with a number sign (#) is assigned to a fragment derived from the thiosemicarbazone core: [C_5_H_2_N_3_S + H]^+^ (calcd. m/z = 137.0042).

 Figure S11. ESI-MS spectrum of **Cu(dc-TSC)_2_** (left) and an enlarged view of the experimental isotope pattern of the detected [M + H]⁺ ion (right). Peaks marked with an asterisk (*) are assigned to leucine enkephalin and its fragments. Leucine enkephalin was used as a lock-mass reference for internal mass calibration. The peak marked with a number sign (#) is assigned to a fragment derived from the thiosemicarbazone core: [C_5_H_2_N_3_S + H]^+^ (calcd. m/z = 137.0042).

 Figure S12. ESI-MS spectrum of **Cu(dc-TSC-oCl)_2_** (left) and an enlarged view of the experimental isotope pattern of the detected [M + H]⁺ ion (right). Peaks marked with an asterisk (*) are assigned to leucine enkephalin and its fragments. Leucine enkephalin was used as a lock-mass reference for internal mass calibration. The peak marked with a number sign (#) is assigned to a fragment derived from the thiosemicarbazone core: [C_5_H_2_N_3_S + H]^+^ (calcd. m/z = 137.0042).

 Figure S13. ESI-MS spectrum of **Cu(dc-TSC-mCl)_2_** (left) and an enlarged view of the experimental isotope pattern of the detected [M + H]⁺ ion (right). Peaks marked with an asterisk (*) are assigned to leucine enkephalin and its fragments. Leucine enkephalin was used as a lock-mass reference for internal mass calibration. The peak marked with a number sign (#) is assigned to a fragment derived from the thiosemicarbazone core: [C_5_H_2_N_3_S + H]^+^ (calcd. m/z = 137.0042).

 Figure S14. ESI-MS spectrum of **Cu(dc-TSC-pCl)_2_** (left) and an enlarged view of the experimental isotope pattern of the detected [M + H]⁺ ion (right). Peaks marked with an asterisk (*) are assigned to leucine enkephalin and its fragments. Leucine enkephalin was used as a lock-mass reference for internal mass calibration.

 Figure S15. ESI-MS spectrum of **Pd(dc-TSC)_2_** (left) and an enlarged view of the experimental isotope pattern of the detected [M + H]⁺ ion (right). Peaks marked with an asterisk (*) are assigned to leucine enkephalin and its fragments. Leucine enkephalin was used as a lock-mass reference for internal mass calibration. The peak marked with a number sign (#) is assigned to a fragment derived from the thiosemicarbazone core: [C_5_H_2_N_3_S + H]^+^ (calcd. m/z = 137.0042).

 Figure S16. ESI-MS spectrum of **Pd(dc-TSC-oCl)_2_** (left) and an enlarged view of the experimental isotope pattern of the detected [M + H]⁺ ion (right). Peaks marked with an asterisk (*) are assigned to leucine enkephalin and its fragments. Leucine enkephalin was used as a lock-mass reference for internal mass calibration. The peak marked with a number sign (#) is assigned to a fragment derived from the thiosemicarbazone core: [C_4_H_4_N_2_S_2_ + H]^+^ (calcd. m/z = 144.9889).

 Figure S17. ESI-MS spectrum of **Pd(dc-TSC-mCl)_2_** (left) and an enlarged view of the experimental isotope pattern of the detected [M + H]⁺ ion (right). Peaks marked with an asterisk (*) are assigned to leucine enkephalin and its fragments. Leucine enkephalin was used as a lock-mass reference for internal mass calibration. The peak marked with a number sign (#) is assigned to a fragment derived from the thiosemicarbazone core: [C_5_H_2_N_3_S + H]^+^ (calcd. m/z = 137.0042).

 Figure S18. ESI-MS spectrum of **Pd(dc-TSC-pCl)_2_** (left) and an enlarged view of the experimental isotope pattern of the detected [M + H]⁺ ion (right). Peaks marked with an asterisk (*) are assigned to leucine enkephalin and its fragments. Leucine enkephalin was used as a lock-mass reference for internal mass calibration. The peak marked with a number sign (#) is assigned to a fragment derived from the thiosemicarbazone core: [C_5_H_2_N_3_S + H]^+^ (calcd. m/z = 137.0042).

 Figure S19. ESI-MS spectrum of **Cu(b-TSC)_2_** (left) and an enlarged view of the experimental isotope pattern of the detected [M + H]⁺ ion (right). Peaks marked with an asterisk (*) are assigned to leucine enkephalin and its fragments. Leucine enkephalin was used as a lock-mass reference for internal mass calibration.

 Figure S20. ESI-MS spectrum of **Cu(b-TSC-oCl)_2_** (left) and an enlarged view of the experimental isotope pattern of the detected [M + H]⁺ ion (right). Peaks marked with an asterisk (*) are assigned to leucine enkephalin and its fragments. Leucine enkephalin was used as a lock-mass reference for internal mass calibration.

 Figure S21. ESI-MS spectrum of **Cu(b-TSC-mCl)_2_** (left) and an enlarged view of the experimental isotope pattern of the detected [M + H]⁺ ion (right). Peaks marked with an asterisk (*) are assigned to leucine enkephalin and its fragments. Leucine enkephalin was used as a lock-mass reference for internal mass calibration.

 Figure S22. ESI-MS spectrum of **Cu(b-TSC-pCl)_2_** (left) and an enlarged view of the experimental isotope pattern of the detected [M + H]⁺ ion (right). Peaks marked with an asterisk (*) are assigned to leucine enkephalin and its fragments. Leucine enkephalin was used as a lock-mass reference for internal mass calibration. The peak marked with a number sign (#) is assigned to a fragment derived from the thiosemicarbazone core: [C_5_H_2_N_3_S + H]^+^ (calcd. m/z = 137.0042).

 Figure S23. ESI-MS spectrum of **Pd(b-TSC)_2_** (left) and an enlarged view of the experimental isotope pattern of the detected [M + H]⁺ ion (right). Peaks marked with an asterisk (*) are assigned to leucine enkephalin and its fragments. Leucine enkephalin was used as a lock-mass reference for internal mass calibration. The peak marked with a number sign (#) is assigned to a fragment derived from the thiosemicarbazone core: [C_4_H_4_N_2_S_2_ + H]^+^ (calcd. m/z = 144.9889).

 Figure S24. ESI-MS spectrum of **Pd(b-TSC-oCl)_2_** (left) and an enlarged view of the experimental isotope pattern of the detected [M + H]⁺ ion (right). Peaks marked with an asterisk (*) are assigned to leucine enkephalin and its fragments. Leucine enkephalin was used as a lock-mass reference for internal mass calibration. The peak marked with a number sign (#) is assigned to a fragment derived from the thiosemicarbazone core: [C_5_H_2_N_3_S + H]^+^ (calcd. m/z = 137.0042).

 Figure S25. ESI-MS spectrum of **Pd(b-TSC-mCl)_2_** (left) and an enlarged view of the experimental isotope pattern of the detected [M + H]⁺ ion (right). Peaks marked with an asterisk (*) are assigned to leucine enkephalin and its fragments. Leucine enkephalin was used as a lock-mass reference for internal mass calibration. The peak marked with a number sign (#) is assigned to a fragment derived from the thiosemicarbazone core: [C_5_H_2_N_3_S + H]^+^ (calcd. m/z = 137.0042).

 Figure S26. ESI-MS spectrum of **Pd(b-TSC-pCl)_2_** (left) and an enlarged view of the experimental isotope pattern of the detected [M + H]⁺ ion (right). Peaks marked with an asterisk (*) are assigned to leucine enkephalin and its fragments. Leucine enkephalin was used as a lock-mass reference for internal mass calibration. The peak marked with a number sign (#) is assigned to a fragment derived from the thiosemicarbazone core: [C_5_H_2_N_3_S + H]^+^ (calcd. m/z = 137.0042).

Figure S27. FTIR spectra of the ligands: top left: **dm-HTSC**; top right: **dm-HTSC-oCl**; bottom left: **dm-HTSC-mCl**; bottom right: **dm-HTSC-pCl**.

Figure S28. FTIR spectra of the complexes: top left: **Cu(dm-TSC)_2_**; top right: **Cu(dm-TSC-oCl)_2_**; bottom left: **Cu(dm-TSC-mCl)_2_**; bottom right: **Cu(dm-TSC-pCl)_2_**.

Figure S29. FTIR spectra of the complexes: top left: **Pd(dm-TSC)_2_**; top right: **Pd(dm-TSC-oCl)_2_**; bottom left: **Pd(dm-TSC-mCl)_2_**; bottom right: **Pd(dm-TSC-pCl)_2_**.

Figure S30. FTIR spectra of the ligands: top left: **dc-HTSC**; top right: **dc-HTSC-oCl**; bottom left: **dc-HTSC-mCl**; bottom right: **dc-HTSC-pCl**.

 Figure S31. FTIR spectra of the complexes: top left: **Cu(dc-TSC)_2_**; top right: **Cu(dc-TSC-oCl)_2_**; bottom left: **Cu(dc-TSC-mCl)_2_**; bottom right: **Cu(dc-TSC-pCl)_2_**.

Figure S32. FTIR spectra of the complexes: top left: **Pd(dc-TSC)_2_**; top right: **Pd(dc-TSC-oCl)_2_**; bottom left: **Pd(dc-TSC-mCl)_2_**; bottom right: **Pd(dc-TSC-pCl)_2_**.

Figure S33. FTIR spectra of the ligands: top left: **b-HTSC**; top right: **b-HTSC-oCl**; bottom left: **b-HTSC-mCl**; bottom right: **b-HTSC-pCl**.

Figure S34. FTIR spectra of the complexes: top left: **Cu(b-TSC)_2_**; top right: **Cu(b-TSC-oCl)_2_**; bottom left: **Cu(b-TSC-mCl)_2_**; bottom right: **Cu(b-TSC-pCl)_2_**.

Figure S35. FTIR spectra of the complexes: top left: **Pd(b-TSC)_2_**; top right: **Pd(b-TSC-oCl)_2_**; bottom left: **Pd(b-TSC-mCl)_2_**; bottom right: **Pd(b-TSC-pCl)_2_**.

Figure S36. UV-Vis-monitored stability of the complexes in a 50:50 (v/v) H_2_O/DMSO medium. Top left: **Cu(dm-TSC)_2_**; top right: **Cu(dm-TSC-oCl)_2_**; bottom left: **Cu(dm-TSC-mCl)_2_**; bottom right: **Cu(dm-TSC-pCl)_2_**.

Figure S37. UV-Vis-monitored stability of the complexes in a 50:50 (v/v) H_2_O/DMSO medium. Top left: **Pd(dm-TSC)_2_**; top right: **Pd(dm-TSC-oCl)_2_**; bottom left: **Pd(dm-TSC-mCl)_2_**; bottom right: **Pd(dm-TSC-pCl)_2_**.

Figure S38. UV-Vis-monitored stability of the complexes in a 50:50 (v/v) H_2_O/DMSO medium. Top left: **Cu(dc-TSC)_2_**; top right: **Cu(dc-TSC-oCl)_2_**; bottom left: **Cu(dc-TSC-mCl)_2_**; bottom right: **Cu(dc-TSC-pCl)_2_**.

Figure S39. UV-Vis-monitored stability of the complexes in a 50:50 (v/v) H_2_O/DMSO medium. Top left: **Pd(dc-TSC)_2_**; top right: **Pd(dc-TSC-oCl)_2_**; bottom left: **Pd(dc-TSC-mCl)_2_**; bottom right: **Pd(dc-TSC-pCl)_2_**.

Figure S40. UV-Vis-monitored stability of the complexes in a 50:50 (v/v) H_2_O/DMSO medium. Top left: **Cu(b-TSC)_2_**; top right: **Cu(b-TSC-oCl)_2_**; bottom left: **Cu(b-TSC-mCl)_2_**; bottom right: **Cu(b-TSC-pCl)_2_**.

Figure S41. UV-Vis-monitored stability of the complexes in a 50:50 (v/v) H_2_O/DMSO medium. Top left: **Pd(b-TSC)_2_**; top right: **Pd(b-TSC-oCl)_2_**; bottom left: **Pd(b-TSC-mCl)_2_**; bottom right: **Pd(b-TSC-pCl)_2_**.

Figure S42. UV-Vis-monitored stability of the complexes in a 90:10 (v/v) DCBM/DMSO medium. Top left: **Cu(dm-TSC)_2_**; top right: **Cu(dm-TSC-oCl)_2_**; bottom left: **Cu(dm-TSC-mCl)_2_**; bottom right: **Cu(dm-TSC-pCl)_2_**.

Figure S43. UV-Vis-monitored stability of the complexes in a 90:10 (v/v) DCBM/DMSO medium. Top left: **Pd(dm-TSC)_2_**; top right: **Pd(dm-TSC-oCl)_2_**; bottom left: **Pd(dm-TSC-mCl)_2_**; bottom right: **Pd(dm-TSC-pCl)_2_**.

Figure S44. UV-Vis-monitored stability of the complexes in a 90:10 (v/v) DCBM/DMSO medium. Top left: **Cu(dc-TSC)_2_**; top right: **Cu(dc-TSC-oCl)_2_**; bottom left: **Cu(dc-TSC-mCl)_2_**; bottom right: **Cu(dc-TSC-pCl)_2_**.

Figure S45. UV-Vis-monitored stability of the complexes in a 90:10 (v/v) DCBM/DMSO medium. Top left: **Pd(dc-TSC)_2_**; top right: **Pd(dc-TSC-oCl)_2_**; bottom left: **Pd(dc-TSC-mCl)_2_**; bottom right: **Pd(dc-TSC-pCl)_2_**.

Figure S46. UV-Vis-monitored stability of the complexes in a 90:10 (v/v) DCBM/DMSO medium. Top left: **Cu(b-TSC)_2_**; top right: **Cu(b-TSC-oCl)_2_**; bottom left: **Cu(b-TSC-mCl)_2_**; bottom right: **Cu(b-TSC-pCl)_2_**.

Figure S47. UV-Vis-monitored stability of the complexes in a 90:10 (v/v) DCBM/DMSO medium. Top left: **Pd(b-TSC)_2_**; top right: **Pd(b-TSC-oCl)_2_**; bottom left: **Pd(b-TSC-mCl)_2_**; bottom right: **Pd(b-TSC-pCl)_2_**.

Figure S48. Bioavailability radars generated for ligands containing 3,4-dimethoxybenzylidene substituents (**dm-HTSC-mCl**, **dm-HTSC-pCl**), 3,4-dichlorobenzylidene substituents (**dc-HTSC-mCl**, **dc-HTSC-pCl**), and 4-bromobenzylidene substitutents (**b-HTSC-mCl**, **b-HTSC-pCl**). The shaded region represents the optimal physicochemical range for oral bioavailability according to the SwissADME model. LIPO – lipophilicity (-0.7 *<* XlogP3 *<* +5.0); SIZE – molecular weight (150 g/mol *<* MW *<* 500 g/mol); POLAR – polarity (20 Å^2^ *<* TPSA *<* 130 Å^2^); INSOLU – insolubility (-6 *<* logS *<* 0); INSATU – fraction of sp^3^-hybridized carbon atoms (0.25 *<* fraction Csp^3^ *<* 1); FLEX – flexibility (0 *<* num. of rotatable bonds *<* 9).

Figure S49. Bioavailability radar generated for DpC as benchmark. The shaded region represents the optimal physicochemical range for oral bioavailability according to the SwissADME model. LIPO – lipophilicity (-0.7 *<* XlogP3 *<* +5.0); SIZE – molecular weight (150 g/mol *<* MW *<* 500 g/mol); POLAR – polarity (20 Å^2^ *<* TPSA *<*130 Å^2^); INSOLU – insolubility (0 *<* logS *<*6); INSATU – fraction of sp^3^-hybridized carbon atoms (0.25 *<* fraction Csp 3 *<* 1); FLEX – flexibility (0 *<* num. of rotatable bonds *<*9).

Table S7. Initial screening of all tested compounds at 10 µM on SK-MEL-28, A-375, and G-361 cells. Rows colored in green represent free organic ligands; blue, Cu(II) complexes; and orange, Pd(II) complexes.

| **Compound** | **SK-MEL-28** | **A-375** | **G-361** |
| --- | --- | --- | --- |
|  | **Cell viability (%)** | | |
| **dm-HTSC** | 118.9 | 100.2 | 101.7 |
| **dm-HTSC-oCl** | 81.2 | 71.9 | 75.1 |
| **dm-HTSC-mCl** | 113.2 | 101.2 | 110.5 |
| **dm-HTSC-pCl** | 73.6 | 88.9 | 90.4 |
| **Cu(dm-TSC)_2_** | 3.6 | 3.2 | 15.8 |
| **Cu(dm-TSC-oCl)_2_** | 35.5 | 89.0 | 89.6 |
| **Cu(dm-TSC-mCl)_2_** | 75.2 | 53.6 | 93.0 |
| **Cu(dm-TSC-pCl)_2_** | 37.2 | 4.0 | 87.0 |
| **Pd(dm-TSC)_2_** | 7.4 | 19.8 | 7.3 |
| **Pd(dm-TSC-oCl)_2_** | 8.6 | 13.9 | 8.2 |
| **Pd(dm-TSC-mCl)_2_** | 16.5 | 65.0 | 8.0 |
| **Pd(dm-TSC-pCl)_2_** | 9.3 | 42.4 | 6.1 |
| **dc-HTSC** | 85.2 | 94.8 | 96.2 |
| **dc-HTSC-oCl** | 68.4 | 76.0 | 78.2 |
| **dc-HTSC-mCl** | 59.1 | 72.0 | 67.9 |
| **dc-HTSC-pCl** | 57.0 | 95.8 | 70.7 |
| **Cu(dc-TSC)_2_** | 68.5 | 86.3 | 72.3 |
| **Cu(dc-TSC-oCl)_2_** | 76.5 | 101.3 | 94.0 |
| **Cu(dc-TSC-mCl)_2_** | 82.3 | 80.2 | 89.0 |
| **Cu(dc-TSC-pCl)_2_** | 75.7 | 77.3 | 81.5 |
| **Pd(dc-TSC)_2_** | 19.3 | 89.2 | 12.8 |
| **Pd(dc-TSC-oCl)_2_** | 71.4 | 84.6 | 73.7 |
| **Pd(dc-TSC-mCl)_2_** | 75.3 | 72.8 | 77.0 |
| **Pd(dc-TSC-pCl)_2_** | 76.1 | 82.8 | 82.9 |
| **b-HTSC** | 89.4 | 103.1 | 99.5 |
| **b-HTSC-oCl** | 70.2 | 101.5 | 82.0 |
| **b-HTSC-mCl** | 78.1 | 109.1 | 109.8 |
| **b-HTSC-pCl** | 94.5 | 112.9 | 91.4 |
| **Cu(b-TSC)_2_** | 40.8 | 8.6 | 81.6 |
| **Cu(b-TSC-oCl)_2_** | 69.7 | 83.6 | 74.9 |
| **Cu(b-TSC-mCl)_2_** | 60.9 | 86.9 | 78.1 |
| **Cu(b-TSC-pCl)_2_** | 53.3 | 105.8 | 78.5 |
| **Pd(b-TSC)_2_** | 8.6 | 41.3 | 5.5 |
| **Pd(b-TSC-oCl)_2_** | 69.7 | 99.5 | 81.0 |
| **Pd(b-TSC-mCl)_2_** | 67.1 | 116.7 | 99.1 |
| **Pd(b-TSC-pCl)_2_** | 66.8 | 98.5 | 83.9 |

Figure S50. Cell viability grahps obtained for the tested compounds on SK-MEL-28, A-375, G-361 cancer cells, as well as normal BJ cells, based on the MTT test results after 48 hours of incubation. Data are presented as % of control culture viability (mean±SD).

Table S8. Relative expression of *ATM, ATR, BAX, BCL-2, CAT, SOD-2, CDKN1A,* and *CCND1* genes in SK-MEL-28 cells following 48 h treatment with **Cu(b-TSC)_2_** complexes, determined by RT-qPCR. Expression levels were normalized to the control group, which was set to 1. Data are presented as mean ± SD (N = 3).

| **Gene** | **SK-MEL-28** | | | | | |
| --- | --- | --- | --- | --- | --- | --- |
|  | **Control** | | | **Cu(b-TSC)_2_** | | |
|  | **Mean** | **SD** | **N** | **Mean** | **SD** | **N** |
| ***ATM*** | 1.007 | 0.145 | 3 | 0.708 | 0.030 | 3 |
| ***ATR*** | 1.001 | 0.052 | 3 | 1.023 | 0.105 | 3 |
| ***BAX*** | 1.006 | 0.128 | 3 | 1.258 | 0.159 | 3 |
| ***BCL-2*** | 1.002 | 0.072 | 3 | 0.985 | 0.109 | 3 |
| ***CAT*** | 1.005 | 0.122 | 3 | 0.981 | 0.022 | 3 |
| ***SOD-2*** | 1.004 | 0.119 | 3 | 1.343 | 0.014 | 3 |
| ***CDKN1A*** | 1.001 | 0.060 | 3 | 3.217 | 0.070 | 3 |
| ***CCND1*** | 1.000 | 0.028 | 3 | 0.821 | 0.007 | 3 |

Table S9. Relative expression of *ATM, ATR, BAX, BCL-2, CAT, SOD-2, CDKN1A*, and *CCND1* genes in A375 cells following 48 h treatment with **Cu(b-TSC)_2_** complexes, determined by RT-qPCR. Expression levels were normalized to the control group, which was set to 1. Data are presented as mean ± SD (N = 3).

| **Gene** | **A375** | | | | | |
| --- | --- | --- | --- | --- | --- | --- |
|  | **Control** | | | **Cu(b-TSC)_2_** | | |
|  | **Mean** | **SD** | **N** | **Mean** | **SD** | **N** |
| ***ATM*** | 1.002 | 0.069 | 3 | 2.220 | 0.101 | 3 |
| ***ATR*** | 1.005 | 0.127 | 3 | 2.411 | 0.104 | 3 |
| ***BAX*** | 1.005 | 0.129 | 3 | 1.129 | 0.049 | 3 |
| ***BCL-2*** | 1.004 | 0.109 | 3 | 0.705 | 0.105 | 3 |
| ***CAT*** | 1.001 | 0.059 | 3 | 1.286 | 0.045 | 3 |
| ***SOD-2*** | 1.001 | 0.040 | 3 | 0.145 | 0.001 | 3 |
| ***CDKN1A*** | 1.002 | 0.071 | 3 | 1.264 | 0.026 | 3 |
| ***CCND1*** | 1.004 | 0.105 | 3 | 0.636 | 0.029 | 3 |

Table S10. Relative expression of *ATM, ATR, BAX, BCL-2, CAT, SOD-2, CDKN1A*, and *CCND1* genes in G361 cells following 48 h treatment with **Pd(dm-TSC-pCl)_2_** complexes, determined by RT-qPCR. Expression levels were normalized to the control group, which was set to 1. Data are presented as mean ± SD (N = 3).

| **Gene** | **G361** | | | | | |
| --- | --- | --- | --- | --- | --- | --- |
|  | **Control** | | | **Pd(dm-TSC-pCI)_2_** | | |
|  | **Mean** | **SD** | **N** | **Mean** | **SD** | **N** |
| ***ATM*** | 1.000 | 0.033 | 3 | 0.972 | 0.083 | 3 |
| ***ATR*** | 1.000 | 0.038 | 3 | 1.011 | 0.063 | 3 |
| ***BAX*** | 1.002 | 0.069 | 3 | 1.310 | 0.028 | 3 |
| ***BCL-2*** | 1.001 | 0.058 | 3 | 1.063 | 0.048 | 3 |
| ***CAT*** | 1.002 | 0.074 | 3 | 0.956 | 0.037 | 3 |
| ***SOD-2*** | 1.001 | 0.052 | 3 | 1.166 | 0.047 | 3 |
| ***CDKN1A*** | 1.001 | 0.051 | 3 | 2.145 | 0.125 | 3 |
| ***CCND1*** | 1.002 | 0.069 | 3 | 1.086 | 0.009 | 3 |

Table S11. Calculated descriptors related to the hydrophilic-lipophilic balance for all 24 investigated complexes. Values for the complexes included in the exploratory regression analysis are shown in bold. Cu(II) and Pd(II) complexes are shaded in blue and orange, respectively.

| **Compound** | **Descriptor** | | | |
| --- | --- | --- | --- | --- |
|  | **logPo/w** | **qPSA_NOS_/SASA** | **logS** | **E_solv_ [kcal/mol]** |
| **Cu(dm-TSC)_2_** | **6.099** | **0.04933** | **-9.923** | **-15.599** |
| **Cu(dm-TSC-oCl)_2_** | **7.230** | **0.04634** | **-11.130** | **-15.594** |
| **Cu(dm-TSC-mCl)_2_** | 7.382 | 0.04177 | -11.130 | -12.344 |
| **Cu(dm-TSC-pCl)_2_** | **7.217** | **0.04653** | **-11.130** | **-15.756** |
| **Pd(dm-TSC)_2_** | 6.392 | 0.04553 | -10.037 | -14.203 |
| **Pd(dm-TSC-oCl)_2_** | 7.427 | 0.04321 | -11.247 | -14.061 |
| **Pd(dm-TSC-mCl)_2_** | 7.685 | 0.03840 | -11.247 | -10.731 |
| **Pd(dm-TSC-pCl)_2_** | 7.423 | 0.04349 | -11.247 | -14.230 |
| **Cu(dc-TSC)_2_** | 8.905 | 0.04046 | -11.903 | -10.240 |
| **Cu(dc-TSC-oCl)_2_** | 9.907 | 0.03822 | -13.113 | -10.486 |
| **Cu(dc-TSC-mCl)_2_** | 10.219 | 0.03247 | -13.113 | -6.973 |
| **Cu(dc-TSC-pCl)_2_** | 9.876 | 0.03879 | -13.113 | -10.873 |
| **Pd(dc-TSC)_2_** | **9.226** | **0.03656** | **-12.017** | **-8.186** |
| **Pd(dc-TSC-oCl)_2_** | 10.233 | 0.03451 | -13.227 | -8.368 |
| **Pd(dc-TSC-mCl)_2_** | 10.557 | 0.02854 | -13.227 | -4.629 |
| **Pd(dc-TSC-pCl)_2_** | 10.180 | 0.03509 | -13.227 | -8.722 |
| **Cu(b-TSC)_2_** | **8.548** | **0.04048** | **-11.067** | **-11.382** |
| **Cu(b-TSC-oCl)_2_** | 9.527 | 0.03821 | -12.267 | -11.680 |
| **Cu(b-TSC-mCl)_2_** | 9.844 | 0.03224 | -12.267 | -8.125 |
| **Cu(b-TSC-pCl)_2_** | 9.371 | 0.03960 | -12.267 | -11.876 |
| **Pd(b-TSC)_2_** | **8.836** | **0.03668** | **-11.180** | **-9.463** |
| **Pd(b-TSC-oCl)_2_** | 9.834 | 0.03453 | -12.383 | -9.696 |
| **Pd(b-TSC-mCl)_2_** | 10.115 | 0.02888 | -12.383 | -6.101 |
| **Pd(b-TSC-pCl)_2_** | 9.784 | 0.03482 | -12.383 | -10.046 |

Table S12. The average growth inhibition zones around the wells containing compounds at a concentrations ranging from 100 mg/L to 1.56 mg/L.

| Compound | Concentration [mg/L] | *Escherichia*  *coli*  ATCC 35218 | *Listeria*  *monocytogenes*  ATCC 15313 | | *Listeria*  *monocytogenes*  ATCC 35152 | | *Staphylococcus aureus*  ATCC 25923 | | *Staphylococcus epidermidis*  ATCC 12228 |  |
| --- | --- | --- | --- | --- | --- | --- | --- | --- | --- | --- |
|  |  | Zone of Inhibition ± SD [mm] | | | | | | | | |
| **dm-HTSC** | 100.00 | 16.00±1.00 | | n.d. | 10.67±0.58 | 11.33±0.58 | | 14.67±0.58 | |  |
|  | 50.00 | 15.33±0.58 | | n.d. | 9.67±0.58 | 10.67±0.58 | | 12.33±0.58 | |  |
|  | 25.00 | 14.33±0.58 | | n.d. | n.d. | 9.33±0.58 | | 10.00±0.00 | |  |
|  | 12.50 | 13.00±1.00 | | n.d. | n.d. | n.d. | | n.d. | |  |
|  | 6.25 | 9.67±0.58 | | n.d. | n.d. | n.d. | | n.d. | |  |
|  | 3.125 | n.d. | | n.d. | n.d. | n.d. | | n.d. | |  |
|  | 1.563 | n.d. | | n.d. | n.d. | n.d. | | n.d. | |  |
| **Cu(dm-TSC)_2_** | 100.00 | 13.67±0.58 | | n.d. | 10.33±0.58 | 14.33±0.58 | | 13.33±0.58 | |  |
|  | 50.00 | 13.00±0.00 | | n.d. | n.d. | 13.67±0.58 | | 12.67±0.58 | |  |
|  | 25.00 | 12.33±0.58 | | n.d. | n.d. | 11.00±0.00 | | 11.33±0.58 | |  |
|  | 12.50 | 12.00±1.00 | | n.d. | n.d. | 9.33±0.58 | | 9.00±0.00 | |  |
|  | 6.25 | 9.67±0.58 | | n.d. | n.d. | n.d. | | n.d. | |  |
|  | 3.125 | n.d. | | n.d. | n.d. | n.d. | | n.d. | |  |
|  | 1.563 | n.d. | | n.d. | n.d. | n.d. | | n.d. | |  |
| **Pd(dm-TSC)_2_** | 100.00 | 13.67±0.58 | | n.d. | 9.33±0.58 | 11.67±0.58 | | 14.33±0.58 | |  |
|  | 50.00 | 12.00±1.00 | | n.d. | n.d. | 10.00±0.00 | | 13.67±0.58 | |  |
|  | 25.00 | 12.33±0.58 | | n.d. | n.d. | 9.67±0.58 | | 12.00±1.00 | |  |
|  | 12.50 | 11.67±0.58 | | n.d. | n.d. | n.d. | | 10.33±0.58 | |  |
|  | 6.25 | 9.67±0.58 | | n.d. | n.d. | n.d. | | n.d. | |  |
|  | 3.125 | n.d. | | n.d. | n.d. | n.d. | | n.d. | |  |
|  | 1.563 | n.d. | | n.d. | n.d. | n.d. | | n.d. | |  |
| **dm-HTSC-oCl** | 100.00 | 15.33±0.58 | | n.d. | n.d. | 12.33±0.58 | | 11 | |  |
|  | 50.00 | 14.33±0.58 | | n.d. | n.d. | 11.67±0.58 | | n.d. | |  |
|  | 25.00 | 13.00±0.00 | | n.d. | n.d. | 10.33±0.58 | | n.d. | |  |
|  | 12.50 | 11.00±1.00 | | n.d. | n.d. | 9.00±0.00 | | n.d. | |  |
|  | 6.25 | n.d. | | n.d. | n.d. | n.d. | | n.d. | |  |
|  | 3.125 | n.d. | | n.d. | n.d. | n.d. | | n.d. | |  |
|  | 1.563 | n.d. | | n.d. | n.d. | n.d. | | n.d. | |  |
| **Cu(dm-TSC-oCl)_2_** | 100.00 | 14.67±0.58 | | n.d. | n.d. | 12.67±0.58 | | 11.33±0.58 | |  |
|  | 50.00 | 13.67±0.58 | | n.d. | n.d. | 11.67±0.58 | | 10.67±0.58 | |  |
|  | 25.00 | 13.00±0.00 | | n.d. | n.d. | 10.67±0.58 | | 9.33±0.58 | |  |
|  | 12.50 | 12.00±1.00 | | n.d. | n.d. | 9.67±0.58 | | n.d. | |  |
|  | 6.25 | n.d. | | n.d. | n.d. | n.d. | | n.d. | |  |
|  | 3.125 | n.d. | | n.d. | n.d. | n.d. | | n.d. | |  |
|  | 1.563 | n.d. | | n.d. | n.d. | n.d. | | n.d. | |  |
| **Pd(dm-TSC-oCl)_2_** | 100.00 | 14.67±0.58 | | n.d. | 10.33±0.58 | 12.67±0.58 | | 12.00±0.00 | |  |
|  | 50.00 | 14.33±0.58 | | n.d. | 9.00±0.00 | 11.67±0.58 | | 10.33±0.58 | |  |
|  | 25.00 | 13.67±0.58 | | n.d. | n.d. | 11.33.±0.58 | | 9.67±0.58 | |  |
|  | 12.50 | 12.67±0.58 | | n.d. | n.d. | 9.67±0.58 | | n.d. | |  |
|  | 6.25 | 9.67±0.58 | | n.d. | n.d. | n.d. | | n.d. | |  |
|  | 3.125 | n.d. | | n.d. | n.d. | n.d. | | n.d. | |  |
|  | 1.563 | n.d. | | n.d. | n.d. | n.d. | | n.d. | |  |
| **dm-HTSC-mCl** | 100.00 | 14.67±0.58 | | n.d. | n.d. | 13.33±0.58 | | 11.33±0.58 | |  |
|  | 50.00 | 13.67±0.58 | | n.d. | n.d. | 12.00±0.00 | | 9.67±0.58 | |  |
|  | 25.00 | 13.67±0.58 | | n.d. | n.d. | 9.67±0.58 | | n.d. | |  |
|  | 12.50 | 12.67±0.58 | | n.d. | n.d. | n.d. | | n.d. | |  |
|  | 6.25 | 9.33±0.58 | | n.d. | n.d. | n.d. | | n.d. | |  |
|  | 3.125 | n.d. | | n.d. | n.d. | n.d. | | n.d. | |  |
|  | 1.563 | n.d. | | n.d. | n.d. | n.d. | | n.d. | |  |
| **Cu(dm-TSC-mCl)_2_** | 100.00 | 13.67±0.58 | | n.d. | 9.33±0.58 | 16.33±0.58 | | 10.67±0.58 | |  |
|  | 50.00 | 13.33±0.58 | | n.d. | n.d. | 14.67±0.58 | | 9.67±0.58 | |  |
|  | 25.00 | 12.00±0.00 | | n.d. | n.d. | 13.00±0.00 | | n.d. | |  |
|  | 12.50 | 11.33±0.58 | | n.d. | n.d. | 12.33±0.58 | | n.d. | |  |
|  | 6.25 | 9.67±0.58 | | n.d. | n.d. | 9.00±0.00 | | n.d. | |  |
|  | 3.125 | n.d. | | n.d. | n.d. | n.d. | | n.d. | |  |
|  | 1.563 | n.d. | | n.d. | n.d. | n.d. | | n.d. | |  |
| **Pd(dm-TSC-mCl)_2_** | 100.00 | 16.00±0.00 | | n.d. | n.d. | 11.00±0.00 | | 10.67±0.58 | |  |
|  | 50.00 | 15.33±0.58 | | n.d. | n.d. | 10.33±0.58 | | 9.33±0.58 | |  |
|  | 25.00 | 14.33±0.58 | | n.d. | n.d. | 9.00±0.00 | | n.d. | |  |
|  | 12.50 | 13.33±0.58 | | n.d. | n.d. | n.d. | | n.d. | |  |
|  | 6.25 | 11.67±0.58 | | n.d. | n.d. | n.d. | | n.d. | |  |
|  | 3.125 | 9.00±0.00 | | n.d. | n.d. | n.d. | | n.d. | |  |
|  | 1.563 | n.d. | | n.d. | n.d. | n.d. | | n.d. | |  |
| **dm-HTSC-pCl** | 100.00 | 13.67±0.58 | | n.d. | n.d. | 14.33±0.58 | | 11.33±0.58 | |  |
|  | 50.00 | 13.33±0.58 | | n.d. | n.d. | 13.00±1.00 | | 9.67±0.58 | |  |
|  | 25.00 | 12.67±0.58 | | n.d. | n.d. | 12.33±0.58 | | n.d. | |  |
|  | 12.50 | 12.00±0.00 | | n.d. | n.d. | 9.33±0.58 | | n.d. | |  |
|  | 6.25 | 11.33±0.58 | | n.d. | n.d. | n.d. | | n.d. | |  |
|  | 3.125 | 9.00±0.00 | | n.d. | n.d. | n.d. | | n.d. | |  |
|  | 1.563 | n.d. | | n.d. | n.d. | n.d. | | n.d. | |  |
| **Cu(dm-TSC-pCl)_2_** | 100.00 | 13.00±1.00 | | n.d. | 9.67±0.58 | 15.00±0.00 | | 14.00±0.00 | |  |
|  | 50.00 | 12.33±0.58 | | n.d. | n.d. | 13.67±0.58 | | 12.00±1.00 | |  |
|  | 25.00 | 11.67±0.58 | | n.d. | n.d. | 12.33±0.58 | | 11.33±0.58 | |  |
|  | 12.50 | 11.33±0.58 | | n.d. | n.d. | 10.00±1.00 | | 9.33±0.58 | |  |
|  | 6.25 | 10.67±0.58 | | n.d. | n.d. | 9.33±0.58 | | n.d. | |  |
|  | 3.125 | 9.33±0.58 | | n.d. | n.d. |  | | n.d. | |  |
|  | 1.563 | n.d. | | n.d. | n.d. | n.d. | | n.d. | |  |
| **Pd(dm-TSC-pCl)_2_** | 100.00 | 14.33±0.58 | | n.d. | n.d. | 11.67±0.58 | | 13.00±1.00 | |  |
|  | 50.00 | 13.67±0.58 | | n.d. | n.d. | 11.00±1.00 | | 12.00±0.00 | |  |
|  | 25.00 | 12.33±0.58 | | n.d. | n.d. | 9.67±0.58 | | 10.33±0.58 | |  |
|  | 12.50 | 11.67±0.58 | | n.d. | n.d. | 9.00±0.00 | | 9.33±0.58 | |  |
|  | 6.25 | 11.00±0.00 | | n.d. | n.d. | n.d. | | n.d. | |  |
|  | 3.125 | 9.33±0.58 | | n.d. | n.d. | n.d. | | n.d. | |  |
|  | 1.563 | n.d. | | n.d. | n.d. | n.d. | | n.d. | |  |
| **dc-HTSC** | 100.00 | 15.33±0.58 | | n.d. | n.d. | 11.33±0.58 | | 12.00±0.00 | |  |
|  | 50.00 | 13.67±0.58 | | n.d. | n.d. | 9.33±0.58 | | 9.33±0.58 | |  |
|  | 25.00 | 12.67±0.58 | | n.d. | n.d. | n.d. | | n.d. | |  |
|  | 12.50 | 12.00±1.00 | | n.d. | n.d. | n.d. | | n.d. | |  |
|  | 6.25 | 9.33±0.58 | | n.d. | n.d. | n.d. | | n.d. | |  |
|  | 3.125 | n.d. | | n.d. | n.d. | n.d. | | n.d. | |  |
|  | 1.563 | n.d. | | n.d. | n.d. | n.d. | | n.d. | |  |
| **Cu(dc-TSC)_2_** | 100.00 | 14.33±0.58 | | n.d. | n.d. | 13.67±0.58 | | 11.33±0.58 | |  |
|  | 50.00 | 14.00±0.00 | | n.d. | n.d. | 12.33±0.58 | | 10.33±0.58 | |  |
|  | 25.00 | 13.33±0.58 | | n.d. | n.d. | 10.00±0.00 | | 9.33±0.58 | |  |
|  | 12.50 | 13.00±1.00 | | n.d. | n.d. | 9.00±0.00 | | n.d. | |  |
|  | 6.25 | 11.67±0.58 | | n.d. | n.d. | n.d. | | n.d. | |  |
|  | 3.125 | 9.33±0.58 | | n.d. | n.d. | n.d. | | n.d. | |  |
|  | 1.563 | n.d. | | n.d. | n.d. | n.d. | | n.d. | |  |
| **Pd(dc-TSC)_2_** | 100.00 | 14.67±0.58 | | n.d. | 9.00±0.00 | 13.00±0.00 | | 11.67±0.58 | |  |
|  | 50.00 | 13.67±0.58 | | n.d. | n.d. | 12.33±0.58 | | 10.33±0.58 | |  |
|  | 25.00 | 13.33±0.58 | | n.d. | n.d. | 11.00±0.00 | | 9.00±0.00 | |  |
|  | 12.50 | 13.33±0.58 | | n.d. | n.d. | 9.33±0.58 | | n.d. | |  |
|  | 6.25 | 12.00±1.00 | | n.d. | n.d. | n.d. | | n.d. | |  |
|  | 3.125 | 9.67±0.58 | | n.d. | n.d. | n.d. | | n.d. | |  |
|  | 1.563 | n.d. | | n.d. | n.d. | n.d. | | n.d. | |  |
| **dc-HTSC-oCl** | 100.00 | 15.67±0.58 | | n.d. | 9.00±0.00 | 10.33±0.58 | | 11.33±0.58 | |  |
|  | 50.00 | 14.33±0.58 | | n.d. | n.d. | 9.00±0.00 | | 9.33±0.58 | |  |
|  | 25.00 | 13.67±0.58 | | n.d. | n.d. | n.d. | | n.d. | |  |
|  | 12.50 | 12.33±0.58 | | n.d. | n.d. | n.d. | | n.d. | |  |
|  | 6.25 | 11.67±0.58 | | n.d. | n.d. | n.d. | | n.d. | |  |
|  | 3.125 | 9.00±0.00 | | n.d. | n.d. | n.d. | | n.d. | |  |
|  | 1.563 | n.d. | | n.d. | n.d. | n.d. | | n.d. | |  |
| **Cu(dc-TSC-oCl)_2_** | 100.00 | 13.00±0.00 | | n.d. | 10.33±0.58 | 10.67±0.58 | | 12 | |  |
|  | 50.00 | 12.67±0.58 | | n.d. | 9.33±0.58 | 9.33±0.58 | | 11.33±0.58 | |  |
|  | 25.00 | 12.33±0.58 | | n.d. | n.d. | n.d. | | 9.00±0.00 | |  |
|  | 12.50 | 11.67±0.58 | | n.d. | n.d. | n.d. | | n.d. | |  |
|  | 6.25 | 11.00±0.00 | | n.d. | n.d. | n.d. | | n.d. | |  |
|  | 3.125 | 9.33±0.58 | | n.d. | n.d. | n.d. | | n.d. | |  |
|  | 1.563 | n.d. | | n.d. | n.d. | n.d. | | n.d. | |  |
| **Pd(dc-TSC-oCl)_2_** | 100.00 | 14.00±0.00 | | n.d. | n.d. | 11.00±0.00 | | 12.33±0.58 | |  |
|  | 50.00 | 13.67±0.58 | | n.d. | n.d. | 9.67±0.58 | | 11.00±0.00 | |  |
|  | 25.00 | 13.33±0.58 | | n.d. | n.d. | n.d. | | 10.33±0.58 | |  |
|  | 12.50 | 12.67±0.58 | | n.d. | n.d. | n.d. | | 9.00±0.00 | |  |
|  | 6.25 | 10.33±0.58 | | n.d. | n.d. | n.d. | | n.d. | |  |
|  | 3.125 | n.d. | | n.d. | n.d. | n.d. | | n.d. | |  |
|  | 1.563 | n.d. | | n.d. | n.d. | n.d. | | n.d. | |  |
| **dc-HTSC-mCl** | 100.00 | 12.00±1.00 | | n.d. | n.d. | 10.67±0.58 | | 11.67±1.00 | |  |
|  | 50.00 | 11.00±0.00 | | n.d. | n.d. | 9.00±0.00 | | 11.00±1.00 | |  |
|  | 25.00 | 10.67±0.58 | | n.d. | n.d. | n.d. | | 10.00±0.00 | |  |
|  | 12.50 | 10.00±0.00 | | n.d. | n.d. | n.d. | | 9.00±0.00 | |  |
|  | 6.25 | 9.67±0.58 | | n.d. | n.d. | n.d. | | n.d. | |  |
|  | 3.125 | n.d. | | n.d. | n.d. | n.d. | | n.d. | |  |
|  | 1.563 | n.d. | | n.d. | n.d. | n.d. | | n.d. | |  |
| **Cu(dc-TSC-mCl)_2_** | 100.00 | 15.33±0.58 | | n.d. | 12.67±0.58 | 11.67±0.58 | | 12.00±0.00 | |  |
|  | 50.00 | 13.67±0.58 | | n.d. | 10.67±0.58 | 10.00±0.00 | | 11.33±0.58 | |  |
|  | 25.00 | 12.67±0.58 | | n.d. | 9.33±0.58 | 9.33±0.58 | | 10.00±0.00 | |  |
|  | 12.50 | 12.00±0.00 | | n.d. | n.d. | n.d. | | 9.00±0.00 | |  |
|  | 6.25 | 10.67±0.58 | | n.d. | n.d. | n.d. | | n.d. | |  |
|  | 3.125 | 10.33±0.58 | | n.d. | n.d. | n.d. | | n.d. | |  |
|  | 1.563 | n.d. | | n.d. | n.d. | n.d. | | n.d. | |  |
| **Pd(dc-TSC-mCl)_2_** | 100.00 | 13.67±0.58 | | n.d. | n.d. | 12.00±1.00 | | 14 | |  |
|  | 50.00 | 12.33±0.58 | | n.d. | n.d. | 9.67±0.58 | | 12 | |  |
|  | 25.00 | 11.67±0.58 | | n.d. | n.d. | n.d. | | 11 | |  |
|  | 12.50 | 11.33±0.58 | | n.d. | n.d. | n.d. | | 9 | |  |
|  | 6.25 | 10.00±1.00 | | n.d. | n.d. | n.d. | | n.d. | |  |
|  | 3.125 | 9.33±0.58 | | n.d. | n.d. | n.d. | | n.d. | |  |
|  | 1.563 | n.d. | | n.d. | n.d. | n.d. | | n.d. | |  |
| **dc-HTSC-pCl** | 100.00 | 12.67±0.58 | | n.d. | n.d. | 12.00±1.00 | | 11.67±0.58 | |  |
|  | 50.00 | 12.33±0.58 | | n.d. | n.d. | 9.33±0.58 | | 10.00±1.00 | |  |
|  | 25.00 | 11.67±0.58 | | n.d. | n.d. | n.d. | | 9.33±0.58 | |  |
|  | 12.50 | 10.00±0.00 | | n.d. | n.d. | n.d. | | 9.00±0.00 | |  |
|  | 6.25 | n.d. | | n.d. | n.d. | n.d. | | n.d. | |  |
|  | 3.125 | n.d. | | n.d. | n.d. | n.d. | | n.d. | |  |
|  | 1.563 | n.d. | | n.d. | n.d. | n.d. | | n.d. | |  |
| **Cu(dc-TSC-pCl)_2_** | 100.00 | 14.00±1.00 | | n.d. | 10.33±0.58 | 11.00±1.00 | | 12.67±0.58 | |  |
|  | 50.00 | 13.33±0.58 | | n.d. | n.d. | 9.67±0.58 | | 11.33±0.58 | |  |
|  | 25.00 | 12.00±0.00 | | n.d. | n.d. | 9.00±0.00 | | 10.00±0.00 | |  |
|  | 12.50 | 12.33±0.58 | | n.d. | n.d. | n.d. | | 9.00±0.00 | |  |
|  | 6.25 | 9.33±0.58 | | n.d. | n.d. | n.d. | | n.d. | |  |
|  | 3.125 | n.d. | | n.d. | n.d. | n.d. | | n.d. | |  |
|  | 1.563 | n.d. | | n.d. | n.d. | n.d. | | n.d. | |  |
| **Pd(dc-TSC-pCl)_2_** | 100.00 | 14.67±0.58 | | n.d. | 9.00±0.00 | 11.33±0.58 | | 12.33±0.58 | |  |
|  | 50.00 | 13.00±0.00 | | n.d. | n.d. | 10.33±0.58 | | 10.67±0.58 | |  |
|  | 25.00 | 12.67±0.58 | | n.d. | n.d. | 9.67±0.58 | | 9.33±0.58 | |  |
|  | 12.50 | 11.33±0.58 | | n.d. | n.d. | n.d. | | n.d. | |  |
|  | 6.25 | 9.00±0.00 | | n.d. | n.d. | n.d. | | n.d. | |  |
|  | 3.125 | n.d. | | n.d. | n.d. | n.d. | | n.d. | |  |
|  | 1.563 | n.d. | | n.d. | n.d. | n.d. | | n.d. | |  |
| **b-HTSC** | 100.00 | 15.00±0.00 | | 9.00±0.00 | 9.33±0.58 | 11.33±0.58 | | 11.33±0.58 | |  |
|  | 50.00 | 14.33±0.58 | | n.d. | n.d. | 10.33±0.58 | | 9.00±0.00 | |  |
|  | 25.00 | 12.33±0.58 | | n.d. | n.d. | 9.00±0.00 | | n.d. | |  |
|  | 12.50 | 11 | | n.d. | n.d. | n.d. | | n.d. | |  |
|  | 6.25 | n.d. | | n.d. | n.d. | n.d. | | n.d. | |  |
|  | 3.125 | n.d. | | n.d. | n.d. | n.d. | | n.d. | |  |
|  | 1.563 | n.d. | | n.d. | n.d. | n.d. | | n.d. | |  |
| **Cu(b-TSC)_2_** | 100.00 | 14.67±0.58 | | 12.67±0.58 | 12.67±0.58 | 15.33±0.58 | | 12.33±0.58 | |  |
|  | 50.00 | 13.00±0.00 | | 11.00±0.00 | 12.00±0.00 | 13.00±1.00 | | 10.67±0.58 | |  |
|  | 25.00 | 11.33±0.58 | | 9.67±0.58 | 11.00±0.00 | 12.00±0.00 | | 9.67.33±0.58 | |  |
|  | 12.50 | 10.33±0.58 | | n.d. | 9.33±0.58 | 11.33±0.58 | | 9.33±0.58 | |  |
|  | 6.25 | 9.33±0.58 | | n.d. | n.d. | 9.33±0.58 | | n.d. | |  |
|  | 3.125 | n.d. | | n.d. | n.d. | n.d. | | n.d. | |  |
|  | 1.563 | n.d. | | n.d. | n.d. | n.d. | | n.d. | |  |
| **Pd(b-TSC)_2_** | 100.00 | 12.67±0.58 | | 9.67±0.58 | 9.00±0.00 | 12.33±0.58 | | 13.33±0.58 | |  |
|  | 50.00 | 11.33±0.58 | | n.d. | n.d. | 11.00±0.00 | | 10.67±0.58 | |  |
|  | 25.00 | 10.00±0.00 | | n.d. | n.d. | 9.33±0.58 | | 9.67±0.58 | |  |
|  | 12.50 | 9.33±0.58 | | n.d. | n.d. | n.d. | | 9.00±0.00 | |  |
|  | 6.25 | n.d. | | n.d. | n.d. | n.d. | | n.d. | |  |
|  | 3.125 | n.d. | | n.d. | n.d. | n.d. | | n.d. | |  |
|  | 1.563 | n.d. | | n.d. | n.d. | n.d. | | n.d. | |  |
| **b-HTSC-oCl** | 100.00 | 15.00±1.00 | | n.d. | 9.00±0.00 | 11.33±0.58 | | 11.67±0.58 | |  |
|  | 50.00 | 13.67±0.58 | | n.d. | n.d. | 10.33±0.58 | | 10.33±0.58 | |  |
|  | 25.00 | 12.33±0.58 | | n.d. | n.d. | 9.00±0.00 | | 9.00±0.00 | |  |
|  | 12.50 | 9.67±0.58 | | n.d. | n.d. | n.d. | | n.d. | |  |
|  | 6.25 | n.d. | | n.d. | n.d. | n.d. | | n.d. | |  |
|  | 3.125 | n.d. | | n.d. | n.d. | n.d. | | n.d. | |  |
|  | 1.563 | n.d. | | n.d. | n.d. | n.d. | | n.d. | |  |
| **Cu(b-TSC-oCl)_2_** | 100.00 | 12.33±0.58 | | n.d. | 9.33±0.58 | 12.67±0.58 | | 13.67±0.58 | |  |
|  | 50.00 | 11.00±0.00 | | n.d. | n.d. | 11.33±0.58 | | 12.33±0.58 | |  |
|  | 25.00 | 9.33±0.58 | | n.d. | n.d. | 10.00±1.00 | | 12.00±0.00 | |  |
|  | 12.50 | n.d. | | n.d. | n.d. | 9.33±0.58 | | 10.33±0.58 | |  |
|  | 6.25 | n.d. | | n.d. | n.d. | n.d. | | 9.00±0.00 | |  |
|  | 3.125 | n.d. | | n.d. | n.d. | n.d. | | n.d. | |  |
|  | 1.563 | n.d. | | n.d. | n.d. | n.d. | | n.d. | |  |
| **Pd(b-TSC-oCl)_2_** | 100.00 | 12.67±0.58 | | n.d. | 10.33±0.58 | 11.33±0.58 | | 12.67±0.58 | |  |
|  | 50.00 | 11.00±0.00 | | n.d. | 9.67±0.58 | 10.33±0.58 | | 11.00±0.00 | |  |
|  | 25.00 | 9.33±0.58 | | n.d. | n.d. | 9.33±0.58 | | 10.67±0.58 | |  |
|  | 12.50 | n.d. | | n.d. | n.d. | n.d. | | 9.00±0.00 | |  |
|  | 6.25 | n.d. | | n.d. | n.d. | n.d. | | n.d. | |  |
|  | 3.125 | n.d. | | n.d. | n.d. | n.d. | | n.d. | |  |
|  | 1.563 | n.d. | | n.d. | n.d. | n.d. | | n.d. | |  |
| **b-HTSC-mCl** | 100.00 | 13.00±1.00 | | n.d. | 9.33±0.58 | 11.33±0.58 | | 11.67±0.58 | |  |
|  | 50.00 | 13.33±0.58 | | n.d. | n.d. | 9.67±0.58 | | 11.33±0.58 | |  |
|  | 25.00 | 12.67±0.58 | | n.d. | n.d. | n.d. | | 10.67±1.00 | |  |
|  | 12.50 | 11.33±0.58 | | n.d. | n.d. | n.d. | | 9.33±0.58 | |  |
|  | 6.25 | 9.00±0.00 | | n.d. | n.d. | n.d. | | n.d. | |  |
|  | 3.125 | n.d. | | n.d. | n.d. | n.d. | | n.d. | |  |
|  | 1.563 | n.d. | | n.d. | n.d. | n.d. | | n.d. | |  |
| **Cu(b-TSC-mCl)_2_** | 100.00 | 12.00±1.00 | | n.d. | 9.67±0.58 | 13.33±0.58 | | 14.33±0.58 | |  |
|  | 50.00 | 11.33±0.58 | | n.d. | n.d. | 11.00±0.00 | | 13.67±0.58 | |  |
|  | 25.00 | 9.67±0.58 | | n.d. | n.d. | 9.67±0.58 | | 11.00±0.00 | |  |
|  | 12.50 | n.d. | | n.d. | n.d. | n.d. | | 9.33±0.58 | |  |
|  | 6.25 | n.d. | | n.d. | n.d. | n.d. | | n.d. | |  |
|  | 3.125 | n.d. | | n.d. | n.d. | n.d. | | n.d. | |  |
|  | 1.563 | n.d. | | n.d. | n.d. | n.d. | | n.d. | |  |
| **Pd(b-TSC-mCl)_2_** | 100.00 | 14.67±0.58 | | n.d. | 10 | 10.33±0.58 | | 10.00±0.00 | |  |
|  | 50.00 | 13.33±0.58 | | n.d. | 9.33±0.58 | 9.00±0.00 | | n.d. | |  |
|  | 25.00 | 12.33±0.58 | | n.d. | n.d. | n.d. | | n.d. | |  |
|  | 12.50 | 11.00±0.00 | | n.d. | n.d. | n.d. | | n.d. | |  |
|  | 6.25 | 9.33±0.58 | | n.d. | n.d. | n.d. | | n.d. | |  |
|  | 3.125 | n.d. | | n.d. | n.d. | n.d. | | n.d. | |  |
|  | 1.563 | n.d. | | n.d. | n.d. | n.d. | | n.d. | |  |
| **b-HTSC-pCl** | 100.00 | 12.00±1.00 | | n.d. | n.d. | 11.33±0.58 | | 11.00±0.00 | |  |
|  | 50.00 | 11.00±0.00 | | n.d. | n.d. | 10.00±0.00 | | 10.67±0.58 | |  |
|  | 25.00 | 10.67±0.58 | | n.d. | n.d. | n.d. | | 9.33±0.58 | |  |
|  | 12.50 | 9.00±0.00 | | n.d. | n.d. | n.d. | | n.d. | |  |
|  | 6.25 | n.d. | | n.d. | n.d. | n.d. | | n.d. | |  |
|  | 3.125 | n.d. | | n.d. | n.d. | n.d. | | n.d. | |  |
|  | 1.563 | n.d. | | n.d. | n.d. | n.d. | | n.d. | |  |
| **Cu(b-TSC-pCl)_2_** | 100.00 | 13.00±1.00 | | n.d. | n.d. | 12.33±0.58 | | 12.00±1.00 | |  |
|  | 50.00 | 12.00±0.00 | | n.d. | n.d. | 11.67±0.58 | | 11.67±0.58 | |  |
|  | 25.00 | 11.67±0.58 | | n.d. | n.d. | 10.67±0.58 | | 10.33±0.58 | |  |
|  | 12.50 | 10.00±0.00 | | n.d. | n.d. | 9.67±0.58 | | 9.67±0.58 | |  |
|  | 6.25 | n.d. | | n.d. | n.d. | n.d. | | n.d. | |  |
|  | 3.125 | n.d. | | n.d. | n.d. | n.d. | | n.d. | |  |
|  | 1.563 | n.d. | | n.d. | n.d. | n.d. | | n.d. | |  |
| **Pd(b-TSC-pCl)_2_** | 100.00 | 12.33±0.58 | | n.d. | n.d. | 10.67±0.58 | | 12.33±0.58 | |  |
|  | 50.00 | 10.00±0.00 | | n.d. | n.d. | 9.33±0.58 | | 11.00±0.00 | |  |
|  | 25.00 | 9.33±0.58 | | n.d. | n.d. | n.d. | | 10.67±0.58 | |  |
|  | 12.50 | n.d. | | n.d. | n.d. | n.d. | | 9.33±0.58 | |  |
|  | 6.25 | n.d. | | n.d. | n.d. | n.d. | | n.d. | |  |
|  | 3.125 | n.d. | | n.d. | n.d. | n.d. | | n.d. | |  |
|  | 1.563 | n.d. | | n.d. | n.d. | n.d. | | n.d. | |  |

n.d.- not detected

Table S13. Primers applied in gene expression evaluation.

| **Target** | **Gene Name** | **Forward sequence** (5′ → 3′) | **Reverse sequence** (5′ → 3′) |
| --- | --- | --- | --- |
| ***ATM*** | ATM Serine/Threonine Kinase | GCCGCGGTTGATACTACTTTG | GCAGCAGGGTGACAATAAACA |
| ***ATR*** | Ataxia telangiectasia and Rad3 related | TGCCATTTCGCCTGACTCAT | ACATGGGTCTTGGCCTTTTCA |
| ***SOD2*** | Superoxide dismutase 2 | CTGGACAAACCTCAGCCCTAAC | AACCTGAGCCTTGGACACCAAC |
| ***CAT*** | Catalase | GTGCGGAGATTCAACACTGCCA | CGGCAATGTTCTCACACAGACG |
| ***CCND1*** | Cyclin D1 | CCGTCCATGCGGAAGATC | GAAGACCTCCTCCTCGCACT |
| ***CDKN1A*** | Cyclin-dependent kinase inhibitor 1A | CCTCATCCCGTGTTCTCCTTT | GTACCACCCAGCGGACAAGT |
| ***BAX*** | BCL2 Associated X, Apoptosis Regulator | TCAGGATGCGTCCACCAAGAAG | TGTGTCCACGGCGGCAATCATC |
| ***BCL-2*** | BCL2 Apoptosis Regulator | ACTGAGTACCTGAACCGGCA | TACAGTTCCACAAAGGCATCCCAG |
| ***RNA18SN5*** | 18S ribosomal N5 | GAAACTGCGAATGGCTCATTAAA | CACAGTTATCCAAGTGGGAGAGG |
| ***B-ACT*** | Actin Beta | AGAGCTACGAGCTGCCTGAC | AGCACTGTGTTGGCGTACAG |
